# Evaluation of Anticancer Activity of 1,3‐Oxazol‐4‐ylphosphonium Salts *in Vitro*

**DOI:** 10.1002/cmdc.202200319

**Published:** 2022-09-15

**Authors:** Mykhailo Brusnakov, Olexandr Golovchenko, Yevheniia Velihina, Oleksandr Liavynets, Victor Zhirnov, Volodymyr Brovarets

**Affiliations:** ^1^ Department of Chemistry of Bioactive Nitrogen-Containing Heterocyclic Bases V. P. Kukhar Institute of Bioorganic Chemistry and Petrochemistry National Academy of Sciences of Ukraine Murmanska st. 1 02094 Kyiv Ukraine; ^2^ Laboratoire COBRA INSA Rouen Normandie Bâtiment IRCOF, rue Tesnière 1 76821 Mont Saint-Aignan Cedex France; ^3^ Department of General Chemistry and Chemistry of Materials Yuriy Fedkovych Chernivtsi National University Kotsyubynsky st. 2 58012 Chernivtsi Ukraine

**Keywords:** Antiproliferation, Bioorganic chemistry, COMPARE correlations, 1,3-Oxazol-4-yltriphenylphosphonium salts, Synthesis

## Abstract

A novel series of 1,3‐oxazol‐4‐yltriphenylphosphonium salts has been synthesized and functionalized. Oxazole derivatives were subjected to NCI *in vitro* assessment. Seven most active derivatives have been selected for five‐dose assay. Among them, compounds **9** ([2‐(4‐methylphenyl)‐5‐[(4‐methylphenyl)sulfanyl]‐1,3‐oxazol‐4‐yl]triphenylphosphonium perchlorate), **1** ([5‐(4‐methylphenyl)amino]‐2‐phenyl‐1,3‐oxazol‐4‐yl]triphenylphosphonium perchlorate) and **4** ([5‐phenyl‐2‐[(4‐methylphenyl)amino]‐1,3‐oxazol‐4‐yl]triphenylphosphonium perchlorate) were the most active against all tested cancer subpanels. Statistical analysis of the total panel data showed average values of parameters of anticancer activity in the range of 0.3–1.1 μM (GI_50_), 1.2–2.5 μM (TGI) and 5–6 μM (LC_50_). It was found that the presence of phenyl or 4‐methylphenyl groups at C(2) and C(5) in the oxazole ring is of critical importance for the manifestation of the anticancer activity. Matrix COMPARE analysis using LC_50_ vector showed a high positive correlation of compound **9** with standard anticancer agents that can directly disrupt mitochondrial function, causing programmed death of cancer cells. The obtained results indicate the anticancer activity of 1,3‐oxazol‐4‐ylphosphonium salts, which could be useful for developing new anticancer drugs. The most active of them can be recommended for further in‐depth studies and synthesis of new derivatives with antitumor activity on their basis.

## Graphical abstract

**Excellent anticancer activity** of 1,3‐oxazol‐4‐ylphosphonium salts has been demonstrated in a total panel of NCI tumor cell lines. The average values of the antitumor activity parameters of the most potent derivatives ranged from 0.3–1.1 μM (GI_50_), 1.2–2.5 μM (TGI), and 5–6 μM (LC_50_). The cytotoxicity vector of the compound **9** showed a high positive correlation with standard antitumor agents that induce apoptosis via a caspase‐dependent intrinsic mitochondrial pathway. The most active compounds (**9**, **1** and **4**) can be recommended for further in‐depth study and synthesis on their basis of new even more effective derivatives with antitumor activity.

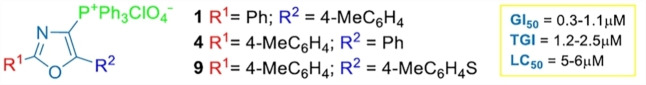

## Introduction

Chemotherapy of malignant neoplasms often leads to drug‐induced secondary tumorigenesis due to nuclear DNA damage caused by traditional genotoxic chemotherapeutic agents, as well as the development of drug resistance due to adaptive genetic mechanisms of cancer cells (drug inactivation, altered drug targets, adaptive responses, and dysfunctional apoptosis). Therefore, the search for compounds with an extragenomic mechanism of antitumor action is of paramount importance in order to avoid possible recurrence of cancer. Among subcellular structures, mitochondria are the most promising target due to the decisive role of these organelles in energy production, induction of apoptosis, and generation of reactive oxygen species.^[1]^ Mitochondria with their own DNA are independent organelles that are not associated with the genetic mechanisms of host cells. Therefore, mitochondrial exposure suggests the ability to bypass the genetic mechanisms underlying tumor recurrence and resistance. This is used as a basis for looking for compounds that may preferentially accumulate in the mitochondria of cancer cells. Targeting this subcellular structure suggests the development of less toxic anticancer drugs compared to genotoxic chemotherapeutic agents, which kill most of the rapidly proliferating tumor and normal cells. Indeed, selective damage to the mitochondria of tumor cells by such agents not only helps to reduce tumor growth, but also minimizes damage to nuclear DNA.^[2]^ Thus, such compounds are able of selectively inducing the death of cancer cells with minimal toxic effects in relation to healthy body cells. Therefore, mitochondriotropic anticancer agents, known as mitokans (short for mitochondria and cancer), which act by destabilizing mitochondria, have good promise in cancer therapy.[^3^, ^4^, ^5^] As you know, the mitochondria of transformed cells show significantly increased transmembrane potentials compared to normal cells.^[6]^ As a consequence, lipophilic phosphonium cations are effective chemical transporters capable of delivering small organic molecules to the mitochondria of tumor cells. Compounds conjugated with phosphonium are small non‐toxic molecules that accumulate in the mitochondria of tumor cells, damaging them, followed by signal induction of cell death processes (apoptosis, necroptosis, or autophagy), which may be useful for their application as antitumor agents.[^7^, ^8^, ^9^, ^10^, ^11^] Another positive property of phosphonium compounds is that they have a prolonged action due to their low biodegradability.^[12]^

This work presents the results of an *in vitro* study of antitumor activity of synthesized phosphonium salts based on the 1,3‐oxazole framework.

## Results and Discussion

### Synthesis of target 1,3‐oxazol‐4‐yltriphenylphosphonium salts

**Scheme 1:**
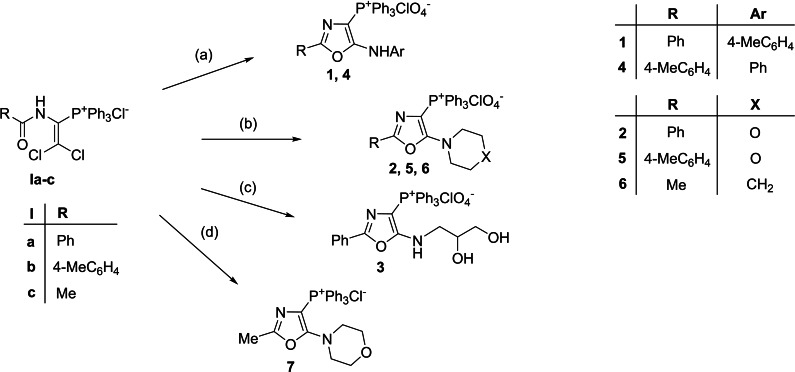
Synthesis of 5‐amino‐1,3‐oxazol‐4‐yltriphenylphosphonium salts **1**–**7**. Reagents and conditions: (a) 1) ArNH_2_, Et_3_N, MeOH, 5 h, 30–40°C 2) NaClO_4_, H_2_O, rt. (b) 1) morpholine or piperidine, MeOH, 8 h, rt. 2) NaClO_4_, H_2_O, rt. (c) 1) 2,3‐dihydroxyaminopropane, MeOH, 10 h, rt. 2) NaClO_4_, H_2_O, rt. (d) morpholine, MeOH, 8 h, rt.

The synthesis of compounds **1**–**14** was accomplished by known approaches.[^13^, ^14^, ^15^, ^16^, ^17^, ^18^, ^19^] 5‐Amino‐1,3‐oxazol‐4‐yltriphenylphosphonium salts **1**–**7** were obtained starting from 2,2‐dichloroethenyl‐1‐acylaminotriphenylphosphonium chlorides (**Ia**–**c**) and aromatic or aliphatic amines (Scheme 1).

**Scheme 2:**
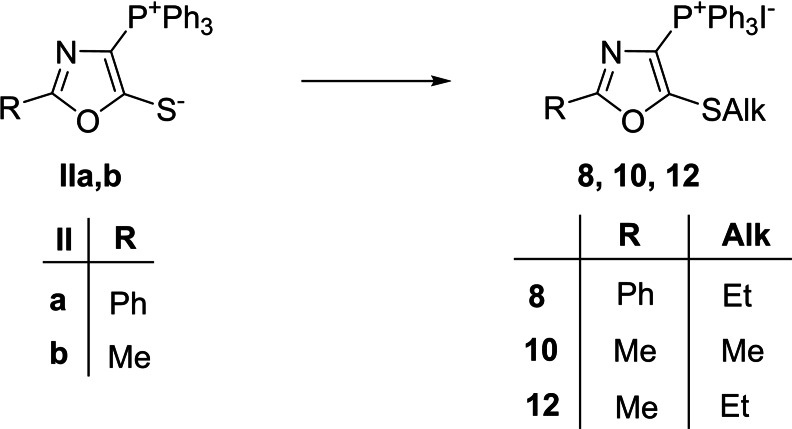
Synthesis of 5‐alkylsulfanyl‐1,3‐oxazol‐4‐ylphosphonium iodides. Reagents and conditions: iodomethane or iodoethane MeOH, 24 h, rt.

To introduce arylamino group into position 5 of oxazole ring, the reaction of compounds **Ia**–**c** with arylamines was performed at 30–40 °C in methanol using triethylamine as a base. The synthesis of 1,3‐oxazol‐4‐yltriphenylphosphonium chlorides with aliphatic amino fragment at C(5) was carried out using an excess of aliphatic amines without the presence of a base and heating. Some of the obtained chlorides were converted into perchlorates **1**–**6** by treating of the reaction mixture with a NaClO_4_‐saturated aqueous solution. 5‐Mercapto‐1,3‐oxazol‐4‐ylphosphonium iodide derivatives (**8**, **10**, **12**) were synthesized by the reaction of betaines **II** with a small excess of methyl or ethyl iodide in methanol at 20–25 °C (Scheme 2).

**Scheme 3:**
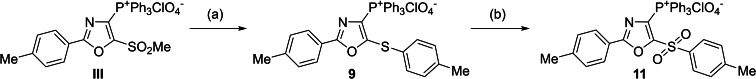
Synthesis of 1,3‐oxazol‐4‐yltriphenylphosphonium perchlorates **9**, **11**. Reagents and conditions: (a) 4‐MeC_6_H_4_S^−^Na^+^, MeOH, 48 h, rt. (b) 1) H_2_O_2_ (30 %), AcOH, 2 h, reflux. 2) NaClO_4_, H_2_O, rt.

The reaction of the mesyl derivative **III** with sodium 4‐methylbenzenethiolate proceeded with the formation of [2‐(4‐methylphenyl)‐5‐[(4‐methylphenyl)sulfanyl]‐1,3‐oxazol‐4‐yl]triphenylphosphonium perchlorate **9**. This transformation took place by boiling the reagents in methanol for 48 h. The oxidation of compound **9** under the action of perhydrol in boiling acetic acid followed by treatment of the reaction mixture with a NaClO_4_‐saturated aqueous solution resulted in a sulfo derivative **11** (Scheme 3).

**Scheme 4:**
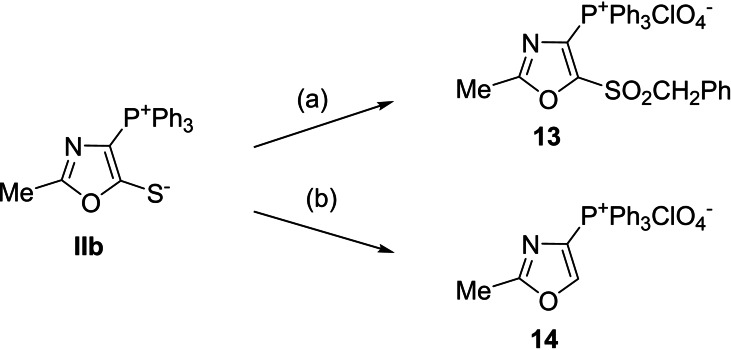
Synthesis of 1,3‐oxazol‐4‐yltriphenylphosphonium perchlorates **13**, **14**. Reagents and conditions: (a) 1) PhCH_2_Cl, MeOH, 16 h, rt. 2) H_2_O_2_ (30 %), AcOH, 2 h, reflux. 3) NaClO_4_, H_2_O, rt. (b) 1) H_2_O_2_ (30 %), AcOH, 0.5 h, reflux. 2) NaClO_4_, H_2_O, 90 °C.

Sulfone **13** was obtained starting from betaine **IIb**. First, alkylation of betaine **IIb** with benzyl chloride was performed at 20–25 °C in methanol, followed by oxidation of the obtained 5‐benzylthio derivative using 30 % hydrogen peroxide in boiling acetic acid. Product **13** was isolated as a perchlorate (Scheme 4).

The 5‐unsubstituted oxazole **14** was synthesized by treating betaine **IIb** with 30 % hydrogen peroxide in boiling glacial acetic acid. For identification, the product was isolated from the reaction mixture in the form of perchlorate by adding a NaClO_4_‐saturated aqueous solution (Scheme 4).

The structures of all obtained compounds were confirmed by elemental analysis, ^1^H‐, ^13^C‐, ^31^P‐NMR spectroscopy and mass spectrometry. Thus, the peaks of the proton signals in the ^1^H NMR spectra of compounds **1**–**14** correspond to these structures. Particular attention is paid to the ^13^C NMR spectroscopy data, since the signals of 1,3‐oxazole carbon nucleus and the phenyl rings of the triphenylphosphonium group appear as doublets due to their interaction with the phosphorus nucleus. Thus, in the 5‐amino‐1,3‐oxazole derivatives **1**–**7**, the carbon nucleus signals at position 2 are manifested in the range of 154.2–156.2 ppm with the coupling constant (*J*) value of 20.5–21.4 Hz, for C(5) – in the range of 160.1–165.4 ppm with the *J* value of 27.2–28.3 Hz, and for C(4) are in the range of 86.7–92.8 ppm with the *J* value of 147.7–150.6 Hz.

In 5‐thio‐1,3‐oxazole derivatives (**8**, **10**, **12**), the doublet signals of C(2) and C(4) are shifted to the weak‐field region and appear at 165.0–165.6 ppm (*J*=19.5–20.2 Hz) and 115.7–122.8 ppm (*J*=135.6–139.4 Hz), respectively, and the doublets of C(5) are at 156.3–160.6 ppm (*J*=28.4–28.9 Hz).

In compounds **II** and **13**, due to the presence of 5‐alkyl(aryl)sulfonyl group, the doublets of C(4) of the oxazole ring are also shifted to the weak‐field region and placed at 126.7 ppm (*J*=128.7 Hz), the doublets of C(2) and C(5) are at 165.0–166.9 ppm (*J*=20.9–21.2 Hz) and 154.7 ppm (*J*=24.7–25.4 Hz), respectively. In 1,3‐oxazole **14**, which does not contain a substituent at position 5 of the heterocyclic fragment, the C(2) doublet is located at 166.1 ppm (19.8 Hz), C5 at 153.7 ppm (31.4 Hz), and C(4) at 121 ppm (135.1 Hz).

The phenyl peaks of the triphenylphosphonium residue of oxazoles **1**–**14** in ^13^C NMR spectra are in the region: C(1) at 116.3–119.2 ppm (93.0–94.0 Hz), C(2) at 130.2–130.7 ppm (13.0–13.7 Hz), C(3) at 134.2–135.0 ppm (11.0–11.2 Hz), and C(4) at 135.0–135.7 ppm (2.5–3.0 Hz).

Signals of phosphorus nucleus in the ^31^P NMR spectra of compounds **1**–**14** were detected at 10.2–15.6 ppm.

In the IR spectra of compounds **1**, **2**, **5**, **6**, **9**, **11**, **13**, **14**, a strong absorption band was found at 1086–1095 cm^−1^, which corresponds to the perchlorate anion.

### In vitro anticancer activity of test compounds

#### One‐dose assay

The synthesized compounds showed a distinctive sensitivity against total NCI panel. According to the degree of growth inhibition, the synthesized compounds were arranged in the following order (the mean percentage of inhibition on 60 cancer cell lines is indicated in parentheses): **1** (138)>**4** (119)>**9** (113)>**8** (103)>**5** (102)>**2** (93)=**11** (93)>**6** (69)>**12** (67)>**10** (44)>**14** (17)>**7** (8)>**13** (6)>**3** (4). As follows from the given sequence, compounds **3**, **7** and **13** practically did not inhibit cell growth. Compounds **6** and **12** displayed moderate growth inhibition of 67–69 %, compounds **2** and **11** had high antiproliferative activity (93 %), while compounds **5** and **8** have shown complete growth reduction of cancer cells. The greatest activity has been demonstrated by compounds **1**, **4** and **9**, which not only stop the growth of cancer cells, but also cause their death. The results of screening for compounds that showed ≥50 % cell growth inhibition tested against panels of 60 cancer cell lines are listed in Table S1. The number reported for the one‐dose assay is growth inhibition (%) relative to the no‐drug control, and relative to the time zero number of cells. This allows the detection of both growth inhibition (values from 0 to 100) and lethality (values greater than 100). A value of 200 means all cells are dead. Percent inhibition data for the tested compounds are given in parentheses.

Among 5‐amino‐substituted 1,3‐oxazoles, derivatives with an aryl amino group (compounds **1** and **4**) turned out to be the most active. Oxazoles containing a morpholine group at C5 were also quite active (compounds **2** and **5**). Replacing the morpholine group with a piperidine substituent leads to a decrease in the activity of oxazole **6**. The substituent in position 2 also plays an important role in the manifestation of activity. Thus, 2‐aryl substituted derivatives were more active than oxazoles with an alkyl group at C2 (compounds **6** and **7**).

Among sulfur‐containing oxazoles, 5‐sulfanyl‐substituted derivatives (compounds **8** and **9**) were the most active. As in the case of 5‐amino substituted oxazoles, the presence of aryl groups in position 2 and 5 significantly increased the activity of these compounds. This applies to both sulfanyl‐ and sulfonyl‐substituted oxazoles.

It can be seen that the most active compound (**1**) at 10 μM does not exhibit cytotoxic effect only against 10 cell lines, 4 of which belonged to the leukemic subpanel. The first 7 compounds from the above sequence, as the most active antitumor agents, were selected for five‐dose assay.

#### Five‐dose assay

The results of the five‐dose assay for compounds that showed high antitumor activity in the primary analysis are presented in the Supporting Information (Table S2).

The obtained data indicate a high antitumor activity of the studied 1,3‐oxazol‐4‐ylphosphonium salts against most cell lines of the total panel. Their statistical analysis made it possible to show that, depending on the analyzed parameter, the compounds form the following series of activity:

50 % Growth inhibition (GI_50_, μM): **9** (0,34±0,02)=**8** (0,39±0,06)>**5** (0,60±0,07)=**1** (0,62±0,05)>**4** (0,77±0,06)>**11** (1,41±0,10)=**2** (2,35±1,04)

Cytostatic action (TGI, μM): **9** (1,34±0,06)>**1** (1,80±0,09)>**4** (2,12±0,10)>**8** (4,04±0,64)=**11** (4,47±0,51)>**5** (6,21±0,92)>**2** (11,29±1,82)

50 % Cytotoxic action (LC_50_, μM): **9** (4,90±0,73)=**1** (5,38±0,38)=**4** (5,68±0,28)>**11** (11,91±1,43)>**8** (24,68±2,25)>**5** (34,79±2,90)=**2** (42.00±4.54)

It should be noted that lines with not precisely defined data with a parameter value of >100 μM were excluded from the statistical analysis, and experimental data for some lines were not obtained (see Table S2).

The rank of antitumor activity of the compounds according to all parameters, calculated as the ratio of the sum of the occupied places in the rows of activity to their number (shown in parentheses), is as follows: **9** (1)>**1** (2.3)>**8** (3.3)>**4** (3.7)>**11** (4.7)>**5** (5.7)>**2** (7).

For a comparative assessment of the antitumor activity of these compounds in relation to individual subpanels, a statistical analysis of their effect on the GI_50_ and TGI parameters was also carried out (Table 1). Statistical data on cytotoxicity due to a significant difference in the sensitivity of cell lines within individual panels were used only for a comparative evaluation of the antitumor activity of the compounds.

**Table 1:** Data of statistical analysis of the antiproliferative activity of the synthesized compounds in terms of 50 % growth inhibition and cytostatic action for individual subpanels.

Subpanel	Values of the anticancer activity parameters of compound (M±m, μM)	
	GI_50_	TGI
Leukemia	**9** (0.29±0.02), **8** (0.30±0.04), **1** (0.33±0.04)**, 4** (0.34±0.05)**, 5** (0.36±0.05), **2** (0.80±0.29), **11** (0.91±0.30)	**9** (1.21±0.24), **1** (1.36±0.29), **4** (1.39±0.28), **5** (3.87±1.36), **11** (4.79±0.78), **8** (5.47±3.77), **2** (27.43±15.39)
	Experimentally not determined for compounds **9** and **11** relative to RPMI‐8226 and SR lines.	
NSLC	**8** (0.28±0.02), **9** (0.31±0.04), **5** (0.58±0.11) **1** (0.59±0.14), **4** (0.79±0.17), **2** (1.72±0.31), **11** (2.14±0.41)	**9** (1.23±0.17)**, 1** (1.82±0.24)**, 4** (2.18±0.31) **8** (3.08±1.18), **11** (7.07±2.41)**, 5** (7.12±1.09), **2** (16.73±5.53)
	Experimentally not determined for compounds **1**, **2**, **5** and **8** relative to NCI−H226 line.	
Colon Cancer	**9** (0.32±0.02), **1** (0.44±0.07), **8** (0.45±0.13)**, 4** (0.51±0.05), **5** (0.79±0.40)**, 11** (1.48±0.11)**, 2** (1.82±1.21)	**9** (1.23±0.11),**1** (1.49±0.14), **4** (1.92±0.16)>**11** (3.39±0.27**)>8** (6.43±1.78)**, 5** (8.23±1.72)**, 2** (10.31±2.87)
	Experimentally not determined for compounds **1**, **2** and **5** relative to COLO 205 line, and **8** to SW‐620 line.	
CNS Cancer	**8** (0.28±0.03), **9** (0.32±0.04), **5** (0.47±0.13), **1** (0.98±0.19), **4** (1.12±0.12), **11** (1.67±0.26)	**9** (1.32±0.13), **1** (2.23±0.26), **4** (2.47±0.10), **11** (4.45±1.28), **5** (6.18±1.91), **8** (6.27±2.11), **2** (9.19±1.93)
Melanoma	**8** (0.29±0.03), **5** (0.40±0.04. **9** (0.45±0.10)=**1** (0.47±0.08), **2** (0.68±0.11), **4** (0.71±0.13), **11** (1.48±0.18)	**8** (1.28±0.31), **9** (1.42±0.21), **1** (1.52±0.14), **4** (2.12±0.25. **11** (3.03±0.30), **5** (3.28±1.12), **2** (4.88±1.24)
	Experimentally not determined for compounds **4** and **8** relative to M14 and UACC‐62 lines, respectively.	
Ovarian Cancer	**9** (0.41±0.05), **5** (0.63±0.24), **1** (0.79±0.19), **4** (0.81±0.19), **8** (1.08±0.49), **11** (1.60±0.30), **2** (2.88±1.34)	**9** (1.75±0.29), **1** (2.02±0.33), **4** (2.13±0.39), **11** (5.57±2.50), **5** (5.84±1.53), **2** (7.20±1.23), **8** (7.86±2.27)
	Experimentally not determined for compounds **1**, **5**, **2** and **8** relative to SK‐OV‐3 line.	
Renal Cancer	**9** (0.31±0.03), **8** (0.60±0.31), **1** (0.74±0.09), **4** (0.92±0.11), **5** (1.36±0.22), **11** (1.43±0.07), **2** (2.33±0.39)	**9** (1.34±0.10), **1** (2.07±0.12), **4** (2.25±0.13), **8** (2.93±1.14), **11** (3.01±0.27), **5** (11.58±5.41), **2** (12.12±2.21)
	Experimentally not determined for compounds **8** relative to UO‐31 line.	
Prostate Cancer	**8** (0,31±0,04), **5** (0,40±0,07), **2** (0,62±0,17), **1** (0,72±0,31), **4** (0,89±0,40), **11** (1,35±0,08), **9** (3,64±0,65)	**9** (1,45±0,08), **1** (2,00±0,42), **4** (2,32±0,31), **8** (2,43±1,14), **11** (2,90±0,38), **5** (6,97±3,42), **2** (9,12±2,69)
Breast Cancer	**8** (0.24±0.03), **9** (0.29±0.02), **5** (0.32±0.04), **2** (0.52±0.13), **1** (0.61±0.17) **4** (0.79±0.24), **11** (1.56±0.13)	**9** (1.22±0.11), **8** (1.40±0.19), **1** (1.83±0.40), **4** (2.22±0.43), **5** (2.68±0.80), **11** (3.84±0.48), **2** (7.74±1.83)

The obtained data make it possible to draw the following conclusions regarding to the antitumor activity of the studied compounds for individual subpanels (the order of the compounds arrangement will further correspond to their average activity in terms of the discussed indicator).

**Leukemia**. All compounds showed high antiproliferative activity in terms of the GI_50_ parameter for this subpanel, with the average values lying in the submicromolar region, which did not differ significantly from each other. According to the TGI parameter, the activity of the compounds **9** and **1** significantly exceeded those of **8** and **11**, and **4** was more active than **2**, **5** and **11**. The activity of compounds **9**, **1** and **4** did not differ significantly among themselves, due to a considerable spread in the sensitivity of individual lines to these compounds. The cytotoxicity of compounds **9** and **8** (LC_50_=23,07±13,73 and >100 μM, respectively) was lower than that of **4** and **1** (8,18±1,25 and 9,05±3,56 μM, respectively), and for other compounds it was significantly lower (LC_50_>41 μM). In addition, the SR line was insensitive to **1** (LC_50_>100 μM). Therefore, compound **4** can be considered the most active antitumor compound in relation to this subpanel.

**NSLC**. Compounds **8**, **9**, **5**, **4** and **1** displayed the highest antiproliferative activity in at least one of the parameters. However, the antitumor activity of compounds **8** and **5** was still inferior to that of compounds **9**, **4** and **1** due to significantly lower cytotoxicity (LC_50_=41.20±4.02 and 28.26±6.64 μM, respectively), which was in the range of 4–5 μM for the last ones.

**Colon Cancer** Compounds **9**, **1**, **8**, **4** and **5** were the most active in terms of the GI_50_ parameter within this subpanel. However, as in the previous case, in terms of cytotoxicity, compounds **8** and **5** (LC_50_=32.58±0.91 and 46.88±11.69 μM, respectively) were significantly inferior to compounds **9**, **1**, and **4**. This allows us to consider compounds **9**, **4** and **1** as equally effective antitumor inhibitors of this subpanel, since their cytotoxicity was also the same (LC_50_=5 μM).

**CNS Cancer**. Oxazoles **8**, **9**, and **5** are among the most active compounds in terms of the GI_50_ parameter. However, the cytostatic action of compounds **8** and **5** was significantly inferior to that of **9**, since the cytotoxic concentration of **9** (LC_50_=4.12±0.32 μM) is much lower than that of compounds **5** and **8** (about 30 μM). This gives reason to consider oxazole **9** as the most active in relation to this subpanel.

**Melanoma**. Compounds **8**, **9**, **1**, and **5** demonstrated the greatest antiproliferative activity. However, the cytotoxic concentration of compounds **5** and **8** was substantially higher (LC_50_=20.60±6.23 and 13.16±4.20 μM, respectively) than that of **9** and **1** (around 4 μM). Consequently, compounds **9** and **1** exhibit the highest antitumor activity against this subpanel.

**Ovarian Cancer**. Oxazoles **9**, **5**, **1**, and **4** showed higher antiproliferative activity than others in at least one parameter. However, compounds **1** and **4** showed lower GI_50_ activity than **9** and **5**, and oxazole **5** displayed a lower TGI effect than compound **9**. In addition, the cytotoxic activity of compound **5** (LC_50_=34.16±9.95 μM) was inferior to that of compound **9**, amounting to 2.02±1.17 μM. This allows us to consider phosphonium salt **9** the most active in relation to the studied subpanel.

**Renal Cancer**. Compound **9** was appreciably superior to the others in both antiproliferative activity parameters (LC_50_ and TGI), and only oxazoles **1** and **11** did not vary considerably in cytotoxicity. Although compound **8** did not significantly differ in the average antiproliferative activity from **9**, the sensitivity of individual lines to it notably (7–12 times) varied among themselves in the value of this indicator. In addition, the cytotoxic concentration of **8** (LC_50_=20.43±3.94 μM) was much lower than that of **9** (3.55±0.61 μM). Therefore, compound **9** is the leader in antitumor activity with respect to considered subpanel.

**Prostate Cancer**. The activity of compounds **9**, **1**, **4**, and **8** did not significantly differ in terms of the GI_50_ parameter and was higher than that of the other oxazoles, but in regards of TGI and LC_50_ activities, compound **8** was considerably inferior to **9**, **1**, and **4**, which thus showed the greatest antitumor activity against this subpanel.

**Breast Cancer**. According to the GI_50_ parameter, the activity of all compounds lying in the submicromolar region was equivalent, with the exception of **11** whose the GI_50_ concentration was significantly higher than that of other compounds. But the cytostatic activity of compounds **9**, **8**, **1** and **4** was notably higher than the activity of other compounds. Since among them the cytotoxicity of **8** was lower than that of other compounds, oxazoles **9**, **1**, and **4** can be considered the most active with respect to studied subpanel.

From the analysis of the dependence of the antitumor effect of 1,3‐oxazole phosphonium salts on their substituents at positions 2 and 5 it can be seen that the presence of a phenyl group or a 4‐methylphenyl substituent at C(2) plays a critical role in the activity of these compounds. Derivatives methylated at this position or containing dihydroxypropylamine group were inactive. Among active compounds, the replacement of the 4‐methylphenyl substituent by the phenyl group at position 2 of compound **9**, and the 4‐methylphenylsulfanyl group by 4‐methylphenylamine at C(5) (compound **1**) somewhat reduced the overall antitumor activity, while the substitution of the 4‐methylphenylsulfanyl group by phenylamine at C(5) with the retention of the 4‐methylphenyl group at position 2 (compound **4**) only aggravated this effect. The same relationship applies to all tested parameters of antitumor activity of these compounds, taken separately.

Based on the obtained data, it can be concluded that among the synthesized derivatives of 1,3‐oxazole phosphonium salts, perchlorates **9**, **1** and **4** ([2‐(4‐methylphenyl)‐5‐[(4‐methylphenyl)sulfanyl]‐1,3‐oxazol‐4‐yl]triphenylphosphonium perchlorate, [5‐(4‐methylphenyl)amino]‐2‐phenyl‐1,3‐oxazol‐4‐yl]triphenylphosphonium perchlorate and [5‐phenyl‐2‐[(4‐methylphenyl)amino]‐1,3‐oxazol‐4‐yl]triphenylphosphonium perchlorate, respectively) are the most promising compounds for further study of their antitumor activity on animal models. For them, the selectivity index was calculated as the ratio of the average value of the corresponding parameter (GI_50_, TGI and LC_50_) for an individual subpanel to that of the total NCI panel (see Table S2). These compounds were found to be non‐selective, showing a broad spectrum of antitumor activity against all tumor subpanels tested, with a selectivity index ranging from 0.6 to 2.4 for all parameters tested (see Table S3).

At the same time, it should be noted that although the assessment of anticancer activity of compounds using only lines of malignant cells without determining the selectivity for normal cells is generally insufficient,^[20]^ but due to the inherent ability of mitocans to accumulate predominantly in the mitochondria of tumor cells, such an assessment is unlikely to be required for them.

### NCI 60 cell panel COMPARE correlations

Standard COMPARE analyses were performed for all parameters using antineoplastic agents of known mode of action as a standard. The following scale of interpretation of pair correlation coefficients was used: insignificant (0.00–0.30), weak (0.30–0.50), moderate (0.50–0.70), high (0.70–0.90) and a very high (0.9–1.0) connection with a standard drug.^[21]^

The COMPARE matrix using the GI_50_ vector showed a moderate positive correlation of compound **9** with Lomustine, which alkylates DNA with subsequent inhibition of DNA and RNA synthesis, and with Actinomycin D, which binds DNA in the transcription initiation complex and prevents RNA strand elongation by the action of RNA polymerase processing (Table 2). For the cytostatic vector, a similar correlation was obtained with 6‐Mercaptopurine and L‐buthionine Sulfoximine (drugs with metabolic mechanism of action). Mercaptopurine inhibits de novo purine synthesis and acts as an antiproliferative agent by interfering with protein, DNA and RNA synthesis and promoting apoptosis.^[47]^ L‐Buthionine‐(S,R)‐Sulfoximine is an inhibitor of gamma‐glutamylcysteine synthetase, glutamate cysteine ligase and reduces glutathione biosynthesis. It has a role as a ferroptosis inducer.^[48]^ Therefore, the data obtained from the COMPARE analysis do not provide sufficient justification to consider these mechanisms as the main ones in the antiproliferative activity of compound **9**.

**Table 2:** Standard agent COMPARE correlations for compounds **9**, **1** and **4**.

Compound	Correlating Drug	Correlation coefficient	Reported Mechanism(s)
**9**	**GI_50_**		
	Lomustine (CCNU)	0.63	Performs major‐groove‐directed DNA alkylation at guanine residues. It also carbamoylates DNA and proteins, resulting in inhibition of DNA and RNA synthesis and disruption of RNA processing.^[22]^
	Actinomycin D	0.57	Inhibits transcription by binding DNA at the transcription initiation complex and preventing elongation of the RNA chain by RNA polymerase.^[23]^
	**TGI**		
	6‐Mercaptopurine	0.63	Inhibit nucleotide interconversions and *de novo* purine synthesis, thereby blocking the formation of purine nucleotides and inhibiting DNA synthesis. This agent is also incorporated into DNA in the form of deoxythioguanosine, which results in the disruption of DNA replication.^[24]^
	L‐Buthionine Sulfoximine	0.56	Suppresses the biosynthesis of gamma‐glutamylcysteine synthetase and glutathione, induces ferroptosis by increasing the level of reactive oxygen species.^[25]^
	**LC_50_**		
	Pyrazoloacridine	0.83	Inhibits the activity of topoisomerases 1 and 2.^[26]^ It also capable of disrupting mitochondrial functions in cancer cells, which induces apoptosis through the caspase‐dependent internal mitochondrial pathway.^[27]^
	Teroxirone	0.79	Activates reactive oxygen species, disrupts mitochondrial function, causing a cytotoxic effect.^[28]^
	Mitotane	0.76	Acts on the energy metabolism of mitochondria with the subsequent initiation of apoptosis.[^29^, ^30^]
	Tamoxifen	0.76	Disrupts the bioenergetic functions of mitochondria, causing changes in respiration, phosphorylation efficiency and membrane structure, causing cell death.^[31]^
	Acodazole Hydrochloride	0.76	Intercalates into DNA, resulting in disruption of DNA replication.^[32]^
	Rapamycin	0.76	Binds to and inhibits the activation of mammalian target rapamycin (mTOR).^[33]^ The drug also induces ultrastructure changes of mitochondria, that results in excessive production of reactive oxygen species, release of cytochrome C, and inhibition of cell proliferation,^[34]^ and induces p53‐independent apoptosis through the mitochondrial pathway.^[35]^
	Cytembena (MBBA)	0.76	Inhibits purine biosynthesis blocking de novo pyrimidine biosynthesis,^[36]^ and disrupted mitochondrial functions.^[37]^

**1**	**GI_50_**		
	Rifamycin SV	0.49	Suppresses RNA and DNA synthesis, tRNA processing and nucleoside uptake.^[38]^
	**TGI**		
	Dichloroallyl Lawsone	0.50	Inhibits dihydroorotate dehydrogenase, which leads to blockade of pyrimidine nucleotide biosynthesis.^[39]^
	**LC_50_**		
	Cytembena	0.65	See above.
	Tetrocarcin‐A	0.59	Inhibits the expression of JAM−A protein and epidermal growth factor receptor‐2 (HER2) and suppresses the expression of an inhibitor of apoptosis proteins (IAP),^[40]^ and suppresses mitochondrial functions, which is accompanied by activation of the caspase‐dependent intrinsic apoptosis pathway.^[41]^
	Lomustine	0.57	See above.
	DUP785 (Brequinar)	0.56	Inhibits dihydroorotate dehydrogenase, thereby blocking de novo pyrimidine biosynthesis,^[42]^ and disrupt mitochondria bioenergetics.^[43]^
	Rapamycin	0.56	See above.

**4**	**GI_50_**		
	Rifamycin SV	0.57	See above.
	Diglycoaldehyde	0.57	Inhibits ribonucleotide reductase, which inhibits DNA synthesis.^[44]^
	**TGI**		
	Pibenzimol Hydrochloride	0.47	Specifically interacts with adenine and thymine of the minor groove of DNA, disrupting its synthesis.^[45]^
	**LC_50_**		
	Hycanthone	0.60	Intercalates into DNA and also targets topoisomerases and apurinic endonuclease 1, disrupting the nucleic acid synthesis.^[46]^
	Pyrazoloacridine	0.59	See above.
	Rapamycin	0.59	See above.

Compound **9** demonstrated high correlation levels at LC_50_ vector with seven standard agents (Pyrazoloacridine, Teroxirone, Mitotane, Tamoxifen, Acodazole Hydrochloride, Rapamycin and Cytembena). Their correlation coefficient values lay in a narrow range (from 0.83 to 0.76). Apparently, the high correlation of cytotoxic mechanisms found between these agents and **9** is largely due to the fact that they are capable to directly disrupt mitochondrial functions in cancer cells by inducing programmed death pathway via apoptosis or ferroptosis.

None of the standard compounds correlated more than “weak” with antiproliferative activity of **1**, indicating the presence of a specific target. Unlike compound **9**, the LC_50_ vector showed only a moderate positive correlation of **1** with standard mitochondrial‐disrupting agents. The effect on mitochondrial activity exhibited by standard DNA‐binding compounds,[^49^, ^50^] apparently, is secondary, caused by damage to mitochondrial DNA.^[51]^

Compound **4** displayed only weak correlation with Pibenzimol Hydrochloride at the TGI vector and moderate correlation with Diglycoaldehyde at that of GI_50_. Its cytotoxic activity was moderately and equally correlated with three standards, two of which also target mitochondria (Pyrazoloacridine and Rapamycin). We have found no studies in the available literature regarding to the effect of Hycanthone on mitochondrial function.

Thus, the antiproliferative activity of the analyzed compounds correlates only weakly (**1**) or moderately (**9** and **4**) with standard drugs, the mechanism of action of which is based on inhibition of nucleic acid replication in the cell nucleus. This does not give grounds to consider this mechanism as the main one for these compounds. Compound **9**, in contrast to **1** and **4**, showed high positive correlations with standard anticancer agents, that can directly disrupt mitochondrial function, causing programmed death of cancer cells. This suggests that its cytotoxicity is directly related to mitochondrial molecular targets that are critical for their normal functioning.

The obtained results provided evidence for the anticancer activities of 1,3‐oxazol‐4‐ylphosphonium salts, which may be useful for the development of new anticancer drugs. The most active of them (**9**, **1** and **4**) can be recommended for further in‐depth studies and synthesis of new derivatives with antitumor activity on their basis.

## Conclusion

This study highlighted the anticancer potential of a new set of 1,3‐oxazol‐4‐ylphosphonium salts. We demonstrated the potent antiproliferative effect of 1,3‐oxazol‐4‐ylphosphonium salts on a panel of tumor cell lines. The greatest activity was shown by compounds **1**, **4** and **9**, which not only stop the growth of cancer cells, but also cause their death. It has been shown that compounds containing phenyl or 4‐methylphenyl radicals in positions 2 and 5 of the oxazole ring as substituents have the highest antitumor activity. Substitution of these functional groups even in one of the indicated positions reduced the activity of the obtained derivatives. Compound **9**, in contrast to **1** and **4**, showed high positive correlations with standard anticancer agents that can directly disrupt mitochondrial function, causing programmed death of cancer cells. This suggests that its cytotoxicity is directly related to mitochondrial molecular targets that are critical for their normal function. Based on the obtained data, it can be concluded that among the synthesized derivatives of phosphonium salts of 1,3‐oxazole, perchlorates **9**, **1** and **4** are the most promising compounds for further study of their antitumor activity.

## Experimental Section

### Chemistry: General Methods

^1^H, ^13^C, ^31^P NMR spectra were obtained on a Bruker AVANCE DRX‐500 or Varian Mercury (400, 125, 151, 162 MHz, respectively) spectrometer (TMS as internal reference or 85 % phosphoric acid as external reference) in DMSO‐d_6_. IR spectra were recorded on a Vertex 70 spectrometer in KBr pellets. Mass spectra were recorded on an Agilent 1100 Series LC‐MS system equipped with a diode array detector Agilent LC\MSD SL (atmospheric pressure chemical ionization). Elemental analysis was carried out in the Analytical Laboratory of the Institute of Bioorganic and Petrochemistry of the National Academy of Sciences of Ukraine by manual methods. The carbon and hydrogen contents were determined using the Pregl gravimetric method, while nitrogen was determined using the Duma's gasometrical micromethod. Chlorine content was determined by the mercurometric method, phosphorus content was determined by the colorimetric method and sulfur content by the Scheininger titrimetric method. M. p. was determined on a Fisher–Johns apparatus and are uncorrected. All reagents and solvents were purchased from commercial sources were used.

***1‐Acylamino‐2**,**2‐dichloroethenyltriphenylphosphonium chlorides I*** were synthesized according to the method described in the article.^[13]^

#### [[5‐(4‐Methylphenyl)amino]‐2‐phenyl‐1,3‐oxazol‐4‐yl]triphenylphosphonium perchlorate (1)

To a solution of 1‐benzoylamino‐2,2‐dichloroethenyltriphenylphosphonium chloride **Ia** (0.01 mol) in 50 mL of methanol triethylamine (0.22 mol) and 4‐methylbenzenamine (0.011 mol) were added. The mixture was stirred at 30–40 °C during 5 hours, then 10 mL of a saturated aqueous NaClO_4_ was added. The formed precipitate was filtered off and the compound **1** was purified by recrystallization from acetonitrile.

Yield 77 %, 4.70 g. Colorless solid, m.p. 250–252°C, (234–236°C).^[14]^ IR (KBr): 3411, 1629, 1580, 1434, 1089, 750, 723, 692, 623, 560, 526 cm^−1^. ^1^H NMR (400 MHz, DMSO‐d_6_): δ 6.63 (s, 1H, NH), 7.94–7.79 (m, 11H, C_6_H_5_), 7.78–7.70 (m, 6H, C_6_H_5_), 7.57–7.48 (m, 3H, C_6_H_5_), 7.10 (d, J=8.3 Hz, 2H, C_6_H_4_), 7.02 (d, J=8.3 Hz, 2H, C_6_H_4_), 2.25 (s, 3H, CH_3_). ^13^C NMR (150.83 MHz, DMSO‐d_6_): δ 160.1 (d, J=28.3 Hz, C(5) oxazolyl), 155.3 (d, J=20.5 Hz, C(2) oxazolyl), 136.8, 135.4 (d, J=2.6 Hz, C(4) C_6_H_5_), 135.0 (d, J=11.0 Hz, C(3) C_6_H_5_), 132.8, 131.4, 130.4 (d, J=13.3 Hz, C(2) C_6_H_5_), 130.1, 129.7, 126.2, 126.0, 119.3, 118.7 (d, J=93.9 Hz, C(1) C_6_H_5_), 92.8 (d, J=147.7 Hz, C(4) oxazolyl), 21.8 (CH_3_). ^31^P NMR (161.92 MHz, DMSO‐d_6_): δ 10.7. LCMS [M−M(ClO_4_^−^)]^+^: 511.2. Anal. Calcd. for C_34_H_28_ClN_2_O_5_P (611.02): C, 66.83; H, 4.62; Cl, 5.80; N, 4.58; P, 5.07. Found: C, 66.92; H, 4.39; Cl, 5.73; N, 4.71; P, 5.11.

#### General procedure for preparing [5‐(2‐alkylamino)‐2‐R‐1,3‐oxazol‐4‐yl]triphenylphosphonium perchlorates (2, 5, 6)

To a solution of one of the compounds **Ia**–**c** (0.01 mol) in methanol (50 mL) was added 0.035 mol of corresponding amine. The mixture was stirred for 8 h at 18–25 °C. The solvent was reduced in vacuo to 1/3 volume and 5 mL of saturated aqueous NaClO_4_ was added to the solution. The precipitate was filtered off and obtained compounds (**2**, **5** and **6**) were purified by recrystallization from methanol.

#### [5‐(Morpholin‐1‐yl)‐2‐phenyl‐1,3‐oxazol‐4‐yl]triphenylphosphonium perchlorate (2)^[15]^

Yield 89 %, 5.26 g. Colorless solid, m.p. 254–256°C, (249–250°C).^[15]^ IR (KBr): 3057, 2964, 2861, 1618, 1603, 1584, 1564, 1438, 1273, 1093 (ClO_4_^−^), 914, 760, 724, 690, 617, 525, 516 cm^−1^. ^1^H NMR (400 MHz, DMSO‐d_6_): δ 7.99–7.84 (m, 11H, C_6_H_5_), 7.83–7.74 (m, 6H, C_6_H_5_), 7.56–7.49 (m, 3H, C_6_H_5_), 3.18–3.12 (m, 4H, 2CH_2_), 3.10–3.03 (m, 4H, 2CH_2_). ^13^C NMR (150.83 MHz, DMSO‐d_6_): δ 165.1 (d, J=27.7 Hz, C(5) oxazolyl), 154.7 (d, J=20.5 Hz, C(2) oxazolyl), 135.6 (d, J=2.9 Hz, C(4) C_6_H_5_), 134.9 (d, J=11.0 Hz, C(3) C_6_H_5_), 131.4, 130.7 (d, J=13.0 Hz, C(2) C_6_H_5_), 129.7, 126.1, 126.0, 119.1 (d, J=93.6 Hz, C(1) C_6_H_5_), 90.9 (d, J=149.1 Hz, C(4) oxazolyl), 65.1 (CH_2_), 50.0 (CH_2_). ^31^P NMR (161.92 MHz, DMSO‐d_6_): δ 12.2. LCMS [M−M(ClO_4_^−^)]^+^: 491.2. Anal. Calcd. for C_31_H_28_ClN_2_O_6_P (590.99): C, 63.00; H, 4.78; Cl, 6.00; N, 4.74; P, 5.24. Found: C, 63.21; H, 4.97; Cl, 5.91; N, 4.85; P, 5.17.

#### [2‐(4‐Methylphenyl)‐5‐(morpholin‐1‐yl)‐1,3‐oxazol‐4‐yl]triphenylphosphonium perchlorate (5)

Yield 92 %, 5.57 g. Colorless solid, m.p. 233–235°C. IR (KBr): 2964, 2923, 2858, 1609, 1583, 1436, 1273, 1086 (ClO_4_^−^), 915, 824, 724, 689, 623, 565, 517 cm^−1^. ^1^H NMR (400 MHz, DMSO‐d_6_): δ 8.02–7.84 (m, 9H, C_6_H_5_), 7.83–7.73 (m, 8H, C_6_H_5_, C_6_H_4_), 7.34 (d, J=8.0 Hz, 2H, C_6_H_4_), 3.19–3.10 (m, 4H, 2CH_2_), 3.08–3.00 (m, 4H, 2CH_2_), 2.36 (s, 3H, CH_3_). ^13^C NMR (125.69 MHz, DMSO‐d_6_): δ 160.1 (d, J=27.2 Hz, C(5) oxazolyl), 155.0 (d, J=20.5 Hz, C(2) oxazolyl), 141.4, 135.6 (d, J=2.9 Hz, C(4) C_6_H_5_), 134.9 (d, J=11.0 Hz, C(3) C_6_H_5_), 130.7 (d, J=13.4 Hz, C(2) C_6_H_5_), 130.2, 126.0, 123.4, 119.1 (d, J=93.5 Hz, C(1) C_6_H_5_), 90.9 (d, J=147.8 Hz, C(4) oxazolyl), 65.1 (CH_2_), 50.1 (CH_2_), 21.5 (CH_3_). ^31^P NMR (161.92 MHz, DMSO‐d_6_): δ 12.1. LCMS [M−M(ClO_4_^−^)]^+^: 505.2. Anal. Calcd. for C_32_H_30_ClN_2_O_6_P (605.02): C, 63.53; H, 5.00; Cl, 5.86; N, 4.63; P, 5.12. Found: C, 63.68; H, 4.81; Cl, 5.81; N, 4.93; P, 5.19.

#### [2‐Methyl‐5‐(piperidin‐1‐yl)‐1,3‐oxazol‐4‐yl]triphenylphosphonium perchlorate (6)

Yield 71 %, 3.74 g. Colorless solid, m.p. 225–227°C. IR (KBr): 2937, 1638, 1581, 1569, 1437, 1086 (ClO_4_^−^), 729, 723, 692, 623, 584, 517 cm^−1^. ^1^H NMR (400 MHz, DMSO‐d_6_): δ 7.95–7.87 (m, 3H, C_6_H_5_), 7.84–7.74 (m, 12H, C_6_H_5_), 2.99–2.88 (m, 4H, 2CH_2_), 2.35 (s, 3H, CH_3_), 1.33–1.23 (m, 2H, CH_2_), 1.08–0.98 (m, 2H, CH_2_). ^13^C NMR (150.83 MHz, DMSO‐d_6_): δ 165.0 (d, J=27.4 Hz, C(5) oxazolyl), 154.2 (d, J=21.4 Hz, C(2) oxazolyl), 135.0 (d, J=2.7 Hz, C(4) C_6_H_5_), 134.2 (d, J=11.5 Hz, C(3) C_6_H_5_), 130.2 (d, J=13.0 Hz, C(2) C_6_H_5_), 119.2 (d, J=93.5 Hz, C(1) C_6_H_5_), 86.7 (d, J=150.6 Hz, C(4) oxazolyl), 50.5 (CH_2_), 24.1 (CH_2_), 22.5 (CH_2_), 13.6 (CH_3_). ^31^P NMR (161.92 MHz, DMSO‐d_6_): δ 12.1. LCMS [M−M(ClO_4_^−^)]^+^: 427.2. Anal. Calcd. for C_27_H_28_ClN_2_O_5_P (526.50): C, 61.54; H, 5.36; Cl, 6.73; N, 5.32; P, 5.88. Found: C, 61.77; H, 5.73; Cl, 6.51; N, 5.65; P, 5.67.

***[5‐[(2,3‐Dihydroxypropyl)amino]‐2‐phenyl‐1,3‐oxazol‐4‐yl]triphenylphosphonium perchlorate (3)*** was synthesized according to the method described in the article.^[16]^

Yield: 69 %, 4.11 g. Colorless solid, m.p. 153–155°C. IR (KBr): 3500, 3281, 1633, 1585, 1439, 1108, 1001, 726, 692, 621, 565, 520 cm^−1^. ^1^H NMR (500 MHz, DMSO‐d_6_): δ 7.94–7.87 (m, 3H, C_6_H_5_), 7.86–7.73 (m, 14H, C_6_H_5_), 7.55–7.47 (m, 3H, C_6_H_5_), 6.79 (t, J=5.5 Hz, 1H, NH), 4.90 (br s, 1H, OH), 4.64 (br s, 1H, OH), 3.60–3.52 (m, 1H, CH), 3.49–3.40 (m, 1H, CH_2_), 3.32–3.20 (m, 3H, 2CH_2_). ^13^C NMR (100.61 MHz, DMSO‐d_6_): δ=164.39 (d, J=29.3 Hz, C(5) oxazolyl), 152.87 (d, J=20.5 Hz, C(2) oxazolyl), 135.42 (d, J=2.2 Hz, C(4) C_6_H_5_), 134.65 (d, J=11.0 Hz, C(3) C_6_H_5_), 130.91, 130.66 (d, J=13.2 Hz, C(2) C_6_H_5_), 129.65, 126.25, 125.75, 119.48 (d, J=93.9 Hz, C(1) C_6_H_5_), 84.01 (d, J=154.1 Hz, C(4) oxazolyl), 70.44, 63.90, 47.21. ^31^P NMR (202.38 MHz, DMSO‐d_6_): δ=10.42. LCMS [M−M(ClO_4_^−^)]^+^: 495.2. Anal. Calcd. for C_30_H_28_ClN_2_O_7_P (594.99): C, 60.56; H, 4.74; Cl, 5.96; N, 4.71; P, 5.21. Found: C, 60.72; H, 5.09; Cl, 6.17; N, 4.98; P, 5.35.

***[5‐Phenyl‐2‐[(4‐methylphenyl)amino]‐1,3‐oxazol‐4‐yl]triphenylphosphonium perchlorate (4)*** was synthesized according to the method described in the article.^[17]^

Yield 79 %, 4.82 g. Light brown powder, m.p. 210–212 °C. ^1^H NMR (500 MHz, DMSO‐d_6_): δ 9.17 (s, 1H, NH), 7.82–7.92 (m, 9H, ArH), 7.69–7.77 (m, 8H, ArH), 7.33 (d, 2H, J=8.0 Hz, ArH), 7.26 (t, 2H, J=6.8 Hz, ArH), 7.07 (d, 2H, J=8.0 Hz, ArH), 7.00 (t, 1H, J=6.8 Hz, ArH), 2.35 (s, 3H, CH_3_). ^13^C NMR (125 MHz, DMSO‐d_6_): δ 159.2 (d, J=28.4 Hz), 155.7 (d, J=20.4 Hz), 141.1, 139.3, 135.0 (d, J=3.0 Hz), 134.6 (d, J=11.0 Hz), 130.0 (d, J=13.8 Hz), 129.7, 129.3, 125.7, 123.1, 122.9, 118.5, 117.8 (d, J=93.8 Hz), 94.4 (d, J=148.1 Hz), 21.1 (CH_3_). ^31^P NMR (162 MHz, DMSO‐d_6_): δ 10.95. LSMS [M−M(ClO_4_^−^)]^+^: 511.3. Anal. Calcd. for C_34_H_28_ClN_2_O_5_P (611.03): C, 66.83; H, 4.62; N, 4.58; P, 5.07. Found: C, 66.55; H, 4.66; N, 4.34; P, 4.87.

#### [2‐Phenyl‐5‐(morpholin‐1‐yl)‐1,3‐oxazol‐4‐yl]triphenylphosphonium chloride (7)

To a solution of 0.01 mol of compound **Ia** in 50 ml of methanol was added 0.035 mol of morpholine, the mixture was stirred for 8 h at 18–25 °C. After reducing the solvent in vacuo to 1/3 volume, 30 mL of methyl *tert*‐butyl ether was added, stirred and left for 0.5 h. The solvents were decanted, residue was kept in vacuo until the solvents were completely removed. 10 mL of cold water was added to the residue and the precipitate was filtered off. The compound **7** was purified by recrystallization from 2‐propanol.

Yield 58 %, 2.69 g. Colorless solid, m.p. 249–251°C. IR (KBr): 3371, 1639, 1579, 1571, 1437, 1358, 1110, 915, 738, 725, 692, 586, 556, 516 cm^−1^. ^1^H NMR (400 MHz, DMSO‐d_6_): δ 7.97–7.88 (m, 3H, C_6_H_5_), 7.87–7.73 (m, 12H, C_6_H_5_), 3.13–3.00 (m, 4H, 2CH_2_), 2.95–2.85 (m, 4H, 2CH_2_), 2.39 (s, 3H, CH_3_). ^13^C NMR (151 MHz, DMSO‐d_6_): δ 165.4 (d, J=27.4 Hz, C(5) oxazolyl), 156.2 (d, J=21.1 Hz, C(2) oxazolyl), 135.6 (d, J=2.9 Hz, C(4) C_6_H_5_), 134.7 (d, J=11.0 Hz, C(3) C_6_H_5_), 130.7 (d, J=13.0 Hz, C(2) C_6_H_5_), 119.0 (d, J=93.3 Hz, C(1) C_6_H_5_), 91.2 (d, J=148.0 Hz, C(4) oxazolyl), 65.1 (CH_2_), 50.2 (CH_2_), 14.3 (CH_3_). ^31^P NMR (161.92 MHz, DMSO‐d_6_): δ 11.4. LCMS [M−M(Cl^−^)]^+^: 429.2. Anal. Calcd. for C_26_H_26_ClN_2_O_2_P (464.47): C, 67.17; H, 5.64; Cl, 7.63; N, 6.03; P, 6.66. Found: C, 67.21; H, 5.93; Cl, 7.41; N, 6.38; P, 6.49.

#### General procedure for preparing [5‐alkylsulfanyl‐2‐R‐1,3‐oxazol‐4‐yl]triphenylphosphonium iodides (8, 10, 12)

To a suspension of betaines **IIa** or **IIb** [6] (0.01 mol) in 30 mL of methanol was added alkyl iodide (0.025 mol) and the mixture was kept at 20–25 °C for 16 h. Solvent was removed in vacuo and a residue was recrystallized from ethanol.

***[5‐Ethylsulfanyl‐2‐phenyl‐1,3‐oxazol‐4‐yl]triphenylphosphonium iodide (8)*** was synthesized according to the method described in the article.^[17]^

Yield 75 %, 4.45 g. Yellow solid, m.p. 202–204°C. ^1^H NMR (500 MHz, DMSO‐d_6_): δ 8.04 (2H, d, J=7.0 Hz, ArH), 7.92–7.96 (3H, m, ArH), 7.78–7.91 (12H, m, ArH), 7.56–7.66 (3H, m, ArH), 3.11 (2H, q, J=7.0 Hz, CH_2_), 1.21 (3H, t, J=7.0 Hz, CH_3_). ^13^C NMR (125 MHz, DMSO‐d_6_): δ 163.9 (d, J=20.1 Hz), 160.4 (d, J=28.6 Hz), 135.7 (d, J=3.0 Hz), 134.6 (d, J=11.0 Hz), 132.2, 130.5 (d, J=13.6 Hz), 129.5, 126.6, 125.2, 119.4, 118.3, 117.4, 116.7, 28.5 (CH_2_), 15.3 (CH_3_). ^31^P NMR (162 MHz, DMSO‐d_6_): δ 10.54. LCMS [M−M(I^−^)]^+^: 466. Anal. Calcd for C_29_H_25_INOPS (593.46): C, 58.69; H, 4.25; N, 2.36; P, 5.22; S, 5.40. Found: C, 58.78; H, 4.33; N, 2.54; P, 5.21; S, 5.34.

#### [2‐Methyl‐5‐methylsulfanyl‐1,3‐oxazol‐4‐yl]triphenylphosphonium iodide (10)

Yield 67 %, 3.47 g. Light yellow solid, m.p. 195–197°C, (200–202 °C).^[14]^ IR (KBr): 1595, 1585, 1489, 1438, 1432, 1107, 755, 722, 688, 560, 539, 515 cm^−1^. ^1^H NMR (400 MHz, DMSO‐d_6_): δ 8.04–7.92 (m, 3H, C_6_H_5_), 7.89–7.75 (m, 12H, C_6_H_5_), 2.58 (s, 3H, CH_3_), 2.47 (s, 3H, CH_3_). ^13^C NMR (125.67 MHz, DMSO‐d_6_): δ 165.1 (d, J=20.2 Hz, C(2) oxazolyl), 160.6 (d, J=28.9 Hz, C(5) oxazolyl), 135.7 (d, J=2.7 Hz, C(4) C_6_H_5_), 134.4 (d, J=11.2 Hz, C(3) C_6_H_5_), 130.5 (d, J=13.2 Hz, C(2) C_6_H_5_), 117.1 (d, J=93.2 Hz, C(1) C_6_H_5_), 115.7 (d, J=139.4 Hz, C(4) oxazolyl), 15.9 (CH_3_), 14.0 (CH_3_). ^31^P NMR (161.92 MHz, DMSO‐d_6_): δ 10.3. LCMS [M−M(I^−^)]^+^: 390.0. Anal. Calcd. for C_23_H_21_INOPS (517.36): C, 53.40; H, 4.09; N, 2.71; P, 5.99; S, 6.20. Found: C, 53.69; H, 4.19; N, 2.85; P, 6.20; S, 6.01.

#### [5‐Ethylsulfanyl‐2‐methyl‐1,3‐oxazol‐4‐yl]triphenylphosphonium iodide (12)

Yield 96 %, 5.10 g. Light yellow solid, m.p. 170–172°C, IR (KBr): 1602, 1587, 1476, 1434, 1110, 760, 727, 689, 563, 539, 519 cm^−1^. ^1^H NMR (400 MHz, DMSO‐d_6_): δ 8.02–7.92 (m, 3H, C_6_H_5_), 7.88–7.74 (m, 12H, C_6_H_5_), 2.94 (q, J=7.1 Hz, 2H, CH_2_), 2.59 (s, 3H, CH_3_), 1.13 (t, J=7.1 Hz, 3H, CH_3_). ^13^C NMR (125.67 MHz, DMSO‐d_6_): δ 165.6 (d, J=20.2 Hz, C(2) oxazolyl), 159.4 (d, J=28.4 Hz, C(5) oxazolyl), 135.6 (d, J=2.5 Hz, C(4) C_6_H_5_), 134.5 (d, J=11.2 Hz, C(3) C_6_H_5_), 130.5 (d, J=13.2 Hz, C(2) C_6_H_5_), 118.0 (d, J=139.4 Hz, C(4) oxazolyl), 117.0 (d, J=93.2 Hz, C(1) C_6_H_5_), 28.4 (CH_2_), 15.2 (CH_3_), 14.1 (CH_3_). ^31^P NMR (161.92 MHz, DMSO‐d_6_): δ 10.5. LCMS [M−M(I^−^)]^+^: 404.1. Anal. Calcd. for C_24_H_23_INOPS (531.39): C, 54.25; H, 4.36; N, 2.64; P, 5.83; S, 6.03. Found: C, 54.31; H, 4.09; N, 2.81; P, 5.72; S, 6.08.

#### [2‐(4‐Methylphenyl)‐5‐[(4‐methylphenyl)sulfanyl]‐1,3‐oxazol‐4‐yl]triphenylphosphonium perchlorate (9)

Sodium 4‐methylbenzenethiolate (0.01 mol) was added to a suspension of [5‐methylsulfanyl‐2‐(4‐methylphenyl)‐1,3‐oxazol‐4‐yl]triphenylphosphonium perchlorate (**III**) (0.011 mol)^[18]^ in dry methanol (100 mL). The resulted mixture was stirred at ambient temperature for 48 h. The formed precipitate of compound **9** was filtered off and crystallized from methanol.

Yield 77 %, 4.94 g. Colorless solid, m.p. 193–195°C, (150–152 °C).^[18]^ IR (KBr): 2962, 2848, 1612, 1553, 1495, 1438, 1091 (ClO_4_^−^), 996, 875, 805, 728, 688, 623, 566, 537, 516 cm^−1^. ^1^H NMR (400 MHz, DMSO‐d_6_): δ 8.02–7.87 (m, 9H, C_6_H_5_), 7.86–7.77 (m, 8H, C_6_H_5_, C_6_H_4_), 7.39 (d, J=8.3 Hz, 2H, C_6_H_4_), 7.26–7.17 (m, 4H, C_6_H_4_), 2.38 (s, 3H, CH_3_), 2.30 (s, 3H, CH_3_). ^13^C NMR (125.67 MHz, DMSO‐d_6_): δ 165.0 (d, J=19.5 Hz, C(2) oxazolyl), 156.3 (d, J=28.4 Hz, C(5) oxazolyl), 142.8, 139.1, 135.7 (d, J=3.0 Hz, C(4) C_6_H_5_), 134.7 (d, J=11.5 Hz, C(3) C_6_H_5_), 131.2, 130.5, 130.4 (d, J=13.2 Hz, C(2) C_6_H_5_), 130.0, 126.7, 125.4, 122.8 (d, J=135.6 Hz, C(4) oxazolyl), 122.3, 116.8 (d, J=93.0 Hz, C(1) C_6_H_5_), 21.2 (CH_3_), 20.7 (CH_3_). ^31^P NMR (161.92 MHz, DMSO‐d_6_): δ 10.9. LCMS [M−M(ClO_4_^−^)]^+^: 542.0. Anal. Calcd. for C_35_H_29_ClNO_5_PS (642.10): C, 65.47; H, 4.55; Cl, 5.52; N, 2.18; P, 4.82; S, 4.99. Found: C, 65.69; H, 4.27; N, 2.37; P, 4.99; S, 4.71.

#### [2‐(4‐Methylphenyl)‐5‐[(4‐methylphenyl)sulfonyl‐1,3‐oxazol‐4‐yl]triphenylphosphonium perchlorate (11)

Compound **9** (0.001 mol) was dissolved in hot (100 °C) glacial acetic acid (20 mL), 30 % of aqueous H_2_O_2_ solution (3×10 mL) was added, heated to reflux and refluxed for a 2 h. The mixture was cooled to ambient temperature, 10 mL of a saturated aqueous NaClO_4_ was added and the formed precipitate of compound **11** was filtered off and crystallized from MeOH.

Yield 71 %, 0.48 g. Colorless solid, m.p. 152–154°C, (150–152 °C).^[18]^ IR (KBr): 3067, 1611, 1593, 1552, 1493, 1439, 1344, 1153, 1094 (ClO_4_^−^), 724, 687, 663, 596, 568, 540, 519 cm^−1^. ^1^H NMR (400 MHz, DMSO‐d_6_): δ 8.03–7.78 (m, 17H, C_6_H_5_, C_6_H_4_), 7.50–7.40 (m, 4H, C_6_H_4_), 7.33 (d, 2H, J=8.3 Hz, C_6_H_4_), 2.42 (s, 3H, CH_3_), 2.40 (s, 3H, CH_3_). ^13^C NMR (125.67 MHz, DMSO‐d_6_): δ 165.0 (d, J=21.2 Hz, C(2) oxazolyl), 154.7 (d, J=25.4 Hz, C(5) oxazolyl), 147.3, 144.0, 135.5 (d, J=2.7 Hz, C(4) C_6_H_5_), 134.8 (d, J=11.2 Hz, C(3) C_6_H_5_), 133.4, 130.6, 130.2 (d, J=13.5 Hz, C(2) C_6_H_5_), 130.1, 128.3, 127.5, 126.7 (d, J=128.7 Hz, C(4) oxazolyl), 121.6, 116.8 (d, J=93.5 Hz, C(1) C_6_H_5_), 21.4 (CH_3_), 21.3 (CH_3_). ^31^P NMR (161.92 MHz, DMSO‐d_6_): δ 15.6. LCMS [M−M(ClO_4_^−^)]^+^: 574.0. Anal. Calcd. for C_35_H_29_ClNO_7_PS (674.10): C, 62.36; H, 4.34; Cl, 5.26; N, 2.08; P, 4.59; S, 4.76. Found: C, 62.58; H, 4.33; N, 2.29; P, 4.63; S, 4.55.

#### [5‐Benzylsulfonyl‐2‐methyl‐1,3‐oxazol‐4‐yl]triphenylphosphonium perchlorate (13)

Benzyl chloride (0.025 mol) was added to a suspension of betaine **IIb** ^[19]^ (0.01 mol) in 100 mL of methanol and the mixture was kept at 20–25 °C for 16 h, then 10 mL of a saturated aqueous NaClO_4_ was added. The formed precipitate was filtered off and crystallized from methanol. Then the precipitate was dissolved in hot (100 °C) glacial acetic acid (20 mL) and 30 % of aqueous H_2_O_2_ solution (3×10 mL) was added, heated to reflux and refluxed for a 2 h. The mixture was cooled to ambient temperature, 10 mL of a saturated aqueous NaClO_4_ was added and the precipitate formed was filtered off. Compound **13** was crystallized from methanol.

Yield 48 %, 2.87 g. Colorless solid, m.p. 120–122°C. IR (KBr): 3061, 1568, 1439, 1344, 1095 (ClO_4_^−^), 731, 688, 636, 623, 555, 529, 520 cm^−1^. ^1^H NMR (400 MHz, DMSO‐d_6_): δ 7.98–7.90 (m, 3H, C_6_H_5_), 7.83–7.69 (m, 12H, C_6_H_5_), 7.49–7.35 (m, 3H, C_6_H_5_), 7.16 (d, J=8.1 Hz, 2H, C_6_H_5_), 4.71 (s, 2H, CH_2_), 2.68 (s, 3H, CH_3_). ^13^C NMR (125.67 MHz, DMSO‐d_6_): δ 166.9 (d, J=20.9 Hz, C(2) oxazolyl), 154.7 (d, J=24.7 Hz, C(5) oxazolyl), 135.6 (d, J=2.7 Hz, C(4) C_6_H_5_), 134.7 (d, J=11.5 Hz, C(3) C_6_H_5_), 131.5, 130.2 (d, J=13.7 Hz, C(2) C_6_H_5_), 129.4, 128.9, 126.7 (d, J=128.7 Hz, C(4) oxazolyl), 125.3, 116.6 (d, J=94.0 Hz, C(1) C_6_H_5_), 60.3 (CH_2_), 14.2 (CH_3_). ^31^P NMR (161.92 MHz, DMSO‐d_6_): δ 15.5. LCMS [M−M(ClO_4_^−^)]^+^: 498.0. Anal. Calcd. for C_29_H_25_ClNO_7_PS (598.00): C, 58.25; H, 4.21; Cl, 5.93; N, 2.34; P, 5.18; S, 5.36. Found: C, 58.48; H, 4.41; N, 2.41; P, 5.04; S, 5.17.

#### [2‐Methyl‐1,3‐oxazol‐4‐yl]triphenylphosphonium perchlorate (14)

Compound (**IV**) (0.01 mol) was dissolved in hot (60 °C) glacial acetic acid (50 mL) and 30 % of aqueous H_2_O_2_ solution (3×40 mL) was added, heated to reflux and refluxed for 0.5 h. To the mixture cooled to ambient temperature, 50 mL of saturated aqueous NaClO_4_ and 50 mL of water was added. The formed precipitate was filtered off and crystallized from methanol yielding compound **14**.

Yield 79 %, 3.51 g. Colorless solid, m.p. 238–240°C, (235–236 °C).^[19]^ IR (KBr): 1587, 1438, 1088 (ClO_4_^−^), 727, 689, 622, 557, 528, 514 cm^−1^. ^1^H NMR (400 MHz, DMSO‐d_6_): δ 8.81 (s, 1H, CH oxazolyl) 8.05–7.93 (m, 3H, C_6_H_5_), 7.87–7.72 (m, 12H, C_6_H_5_), 2.62 (s, 3H, CH_3_). ^13^C NMR (125.67 MHz, DMSO‐d_6_): δ 166.1 (d, J=19.8 Hz, C(2) oxazolyl), 153.7 (d, J=31.4 Hz, C(5) oxazolyl), 135.7 (d, J=2.7 Hz, C(4) C_6_H_5_), 134.2 (d, J=11.2 Hz, C(3) C_6_H_5_), 130.2 (d, J=13.2 Hz, C(2) C_6_H_5_), 121.1 (d, J=135.1 Hz, C(4) oxazolyl), 117.0 (d, J=93.2 Hz, C(1) C_6_H_5_), 13.7 (CH_3_). ^31^P NMR (161.92 MHz, DMSO‐d_6_): δ 10.2. LCMS [M−M(ClO_4_^−^)]^+^: 344.2. Anal. Calcd. for C_22_H_19_ClNO_5_P (443.82): C, 59.54; H, 4.32; Cl, 7.99; N, 3.16; P, 6.98. Found: C, 59.58; H, 4.59; N, 3.33; P, 7.14.

### In Vitro Anticancer Screening of Test Compounds

#### One‐dose full NCI 60 cell panel assay

Synthesized compounds (**1**–**14**) were submitted to National Cancer Institute (NCI), Bethesda, Maryland, U.S.A., under the Developmental Therapeutic Program DTP. The cell line panel engaged a total of 60 different human tumor cell lines derived from nine cancer types, including lung, colon, melanoma, renal, ovarian, brain, leukemia, breast and prostate.

Primary *in vitro* one‐dose anticancer screening was initiated by cell inoculating of each 60 panel lines into a series of standard 96‐well microtiter plates at 5000–40000 cells/well in RPMI 1640 medium containing 5 % fetal bovine serum and 2 mM L‐glutamine (day 0), and then preincubated in absence of drug at 37 °C and 5 % CO_2_ for 24 h. Test compounds were then added into the plates at one concentration of 10^−5^ M (day 1), followed by incubation for a further 48 h at the same conditions. Then the media was removed, the cells were fixed *in situ*, washed, and dried (day 3). The sulforhodamine B assay was used for cell density determination, based on the measurement of cellular protein content. After an incubation period, cell monolayers were fixed with 10 % (wt/vol) trichloroacetic acid and stained for 30 min, after which the excess dye was removed by washing repeatedly with 1 % (vol/vol) acetic acid. The bound stain was resolubilized in 10 mM Tris base solution and measured spectrophotometrically on automated microplate readers for OD determination at 510 nm.^[52]^

#### Five‐dose full NCI 60 cell panel assay

Compounds which exhibit significant growth inhibition in the One‐Dose Screen were evaluated against the 60 cell panel at five concentration levels (0.01, 0.1, 1, 10 and 100 μM). The outcomes were used to create three dose‐response parameters (GI_50_, TGI and LC_50_) calculated for each cell line. The GI_50_ value (drug potency) is measure of the sensitivity of a cell to the effect of the drug, and corresponds to the concentration of the compound causing 50 % decrease in net cell growth. The TGI (drug efficacy) refers to the maximum effect of a drug and is the concentration of the study drug that causes total inhibition of cell growth. The LC_50_ value (cytotoxic activity) is the concentration of the compound causing net 50 % loss of initial cells at the end of the incubation period of 48 h.

The three dose‐response parameters GI_50_, TGI and LC_50_ were calculated for each experimental compound. Data calculations were performed according to the method described by the NCI/NIH Development Therapeutics Program.^[52]^

The GI_50_ are determined as the drug concentrations result in a 50 % growth reduction at 48 h drug exposure. Growth inhibition of 50 % (GI_50_) is calculated from:

$$[\left(T-T{}_{0}\right)/\left(C-T{}_{0}\right)]\times 100=50.$$

The TGI is calculated from:

$$100\times \left(T-T{}_{0}\right)/\left(C-T{}_{0}\right)=0.$$

Thus, the TGI signifies a cytostatic effect.

The LC_50_, which means a cytotoxic effect, is calculated as:

$$\left[\left(T-T{}_{0}\right)/T{}_{0}\right]\times 100=-50{}_{.}$$

### Data Analysis

#### Statistical Data Analysis

Statistical analysis of the results and the curve fitting was performed by means of the program of Statistica v6.0 for Windows. Significant statistical differences between two groups were evaluated using the unpaired Student t‐test (p<0.05). The data are given as means±SEM (standard error of mean); n represents the number of the cell suspensions studied.

#### NCI 60 cell panel COMPARE correlations

The graph of mean values for each of test compounds **9**, **4** and **1** was subsequently used to run the COMPARE algorithm from the Developmental Therapeutics Program (NCI) and calculate the correlation coefficient with respect to compounds from the standard agent database with a known mechanism of action.^[53]^ Pairwise correlation coefficients of greater than 0.5 were used as the cut‐off for assessing whether two agents were likely to share a similar mechanism of action. Briefly, vectors of GI_50_, TGI, and LC_50_ concentrations for tested compound were correlated with the set of average GI_50_, TGI, and LC_50_ vectors for all public NCI‐60 vectors, that had SD exceeding 0.5 for the full public standard agent database.

## Conflict of interest

The authors declare no conflict of interest.

1

## Supporting information

As a service to our authors and readers, this journal provides supporting information supplied by the authors. Such materials are peer reviewed and may be re‐organized for online delivery, but are not copy‐edited or typeset. Technical support issues arising from supporting information (other than missing files) should be addressed to the authors.

Supporting Information

## Data Availability

The data that support the findings of this study are available in the supplementary material of this article.
