# Supplementary material for: Evaluation of Anticancer Activity of 1,3‐Oxazol‐4‐ylphosphonium Salts in Vitro
Source: ChemMedChem. 2022 Sep 15;17(20):e202200319. doi: 10.1002/cmdc.202200319 (PMC9825890; doi:10.1002/cmdc.202200319)
Supplement: Supplementary file 1 — Supporting Information [file CMDC-17-0-s001.pdf]

ChemMedChem

Supporting Information

Evaluation of Anticancer Activity of 1,3-Oxazol-4-ylphosphonium Salts *in Vitro*

Mykhailo Brusnakov, Olexandr Golovchenko, Yevheniia Velihina,* Oleksandr Liavynets, Victor Zhirnov, and Volodymyr Brovarets*

Table of Contents

I. ^{1}H, ^{13}C and ^{31}P NMR, IR and Mass Spectra………………………………………………………… S2-S69

II. Table S1. Data from an one-dose (10^{-5} µM) antitumor screening of synthesized compounds against human tumor cell lines NCI-60, whose growth was inhibited by more than 50%…………………………………………………… S70-S73

III. Table S2. Values of the anticancer activity parameters (GI_{50}, TGI and LC_{50}, µM) for the most active compounds against the NCI 60 human cancer cell lines (five-dose assay)……………………………………………………………………………………… S74-S76

IV. Table S3. Selectivity indices of compounds **1, 4** and **9**…………………………………… S77

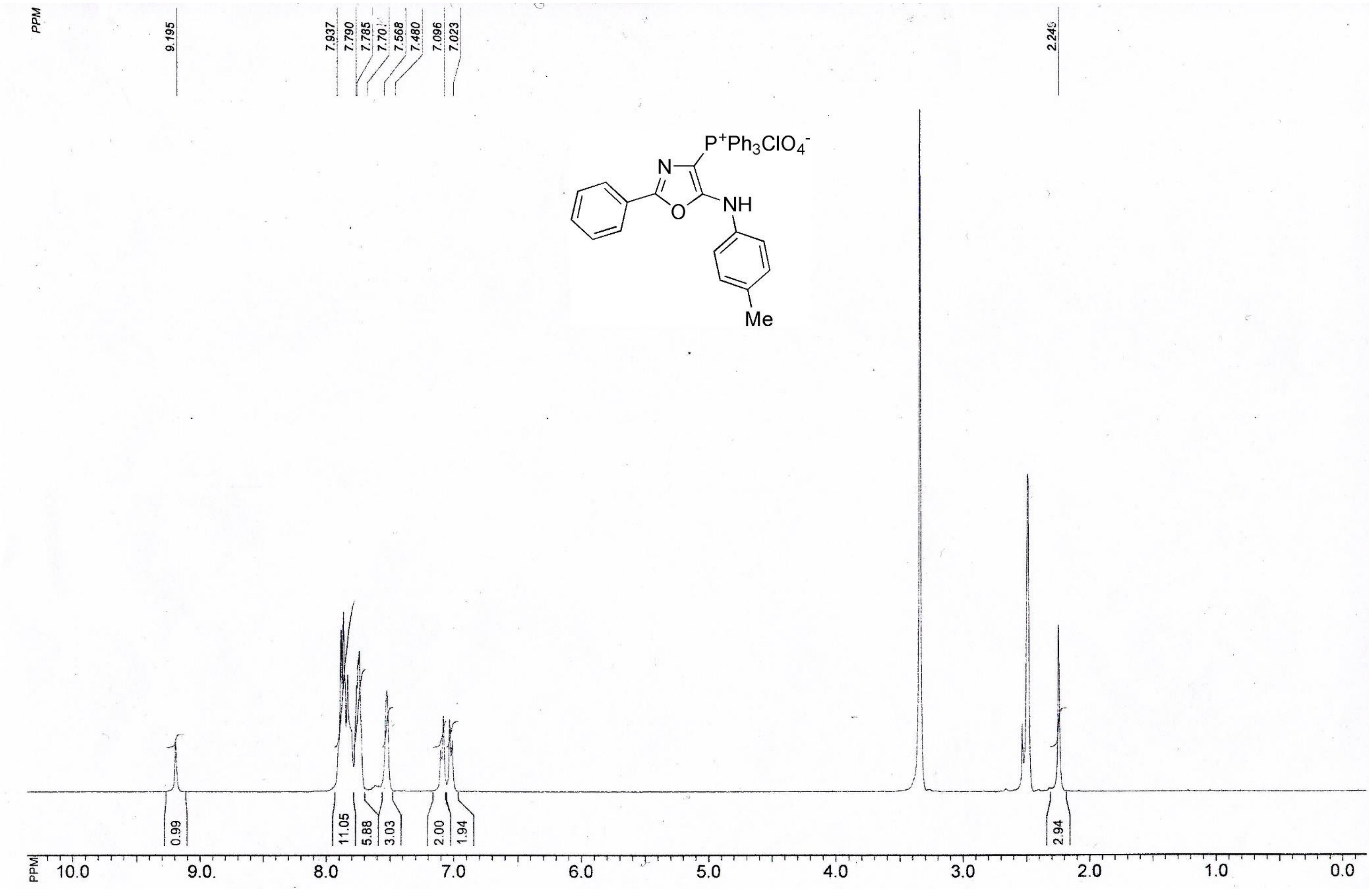

Figure S1. ^{1}H NMR (400 MHz, 294 K, DMSO-d_6) spectrum of compound (**1**).

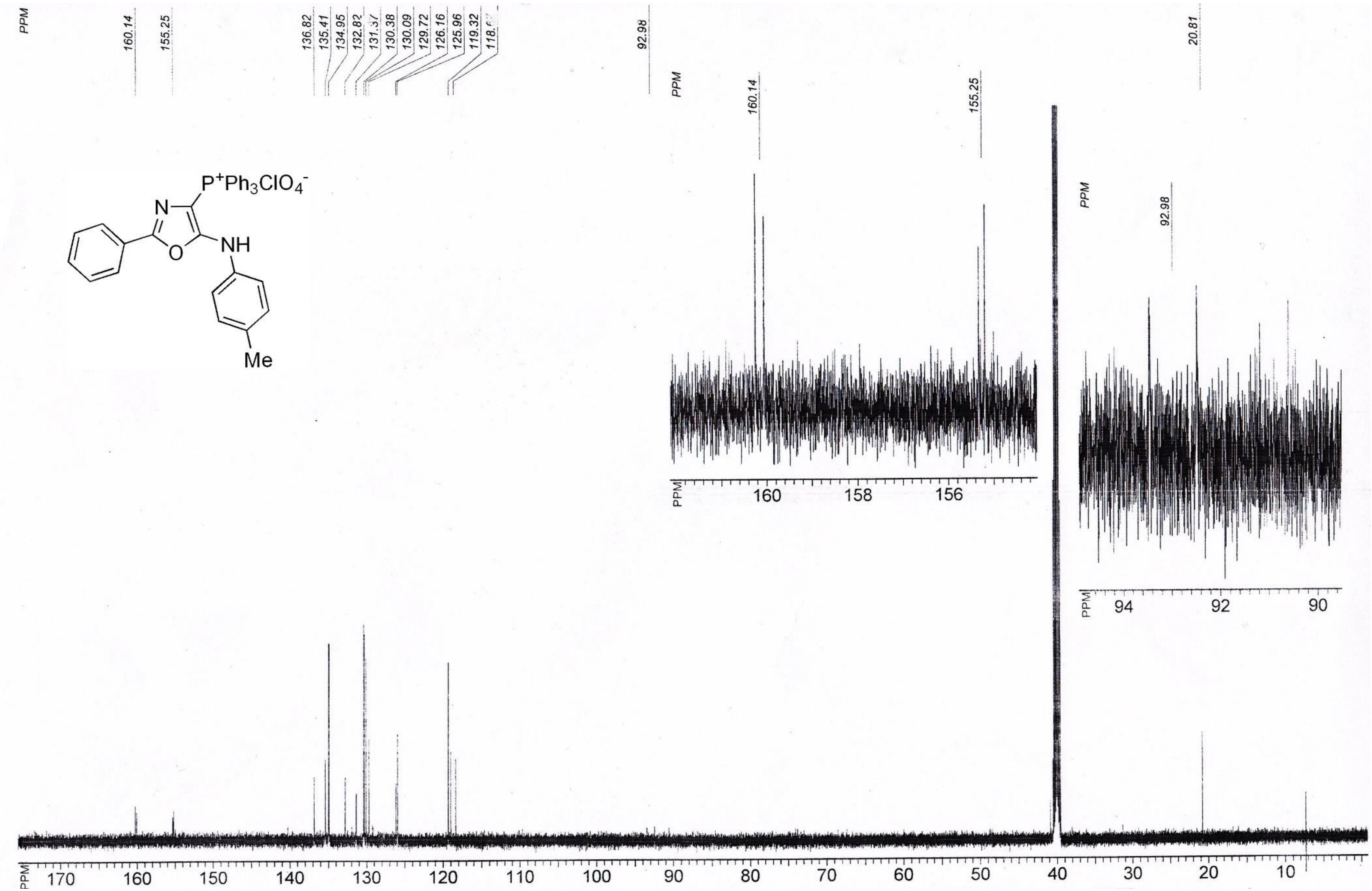

Figure S2. ^{13}C NMR (151 MHz, 298 K, DMSO-d_6) spectrum of compound (**1**).

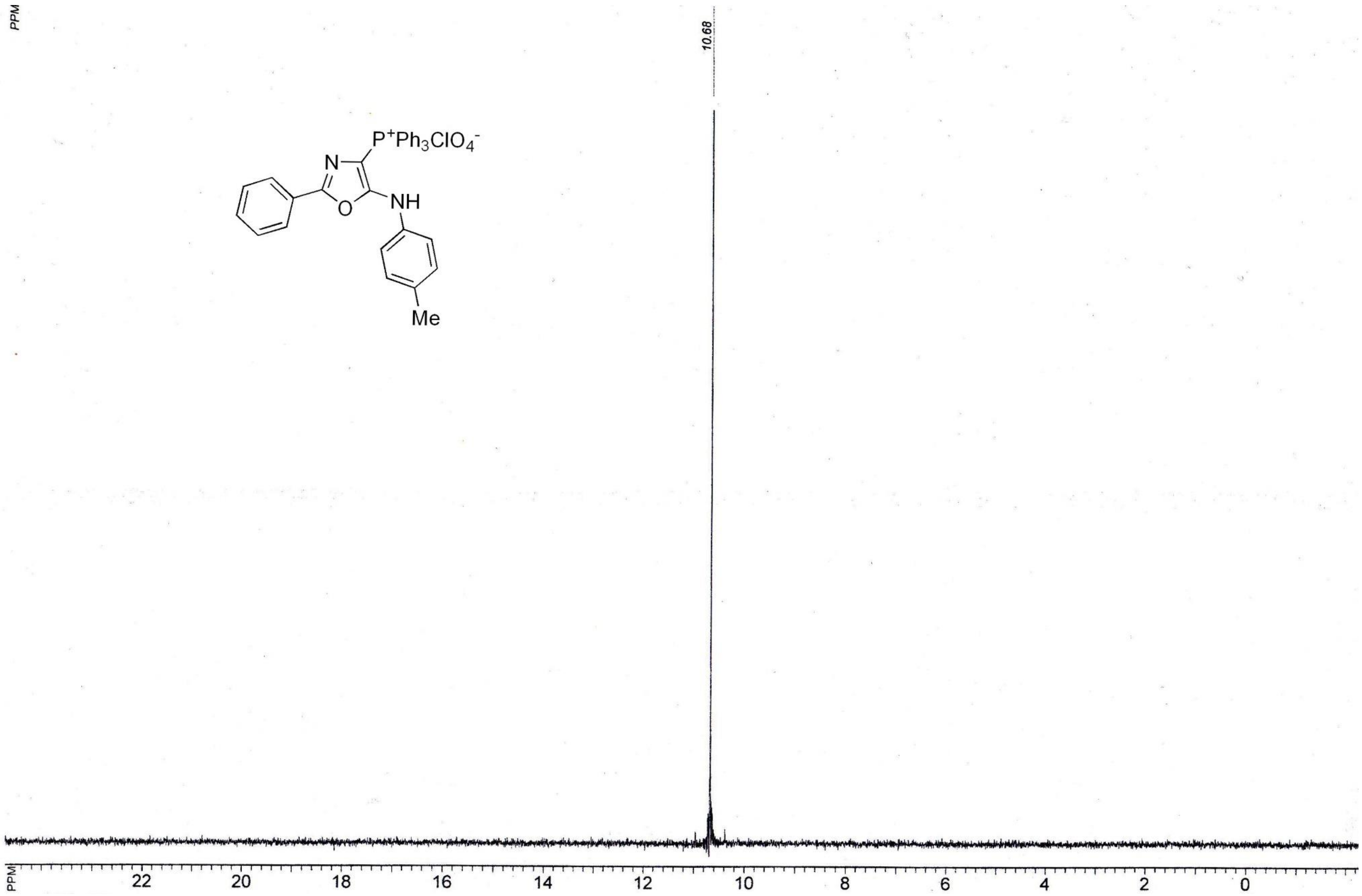

Figure S3. ^{31}P NMR (102 MHz, 294 K, DMSO-d_6) spectrum of compound (**1**).

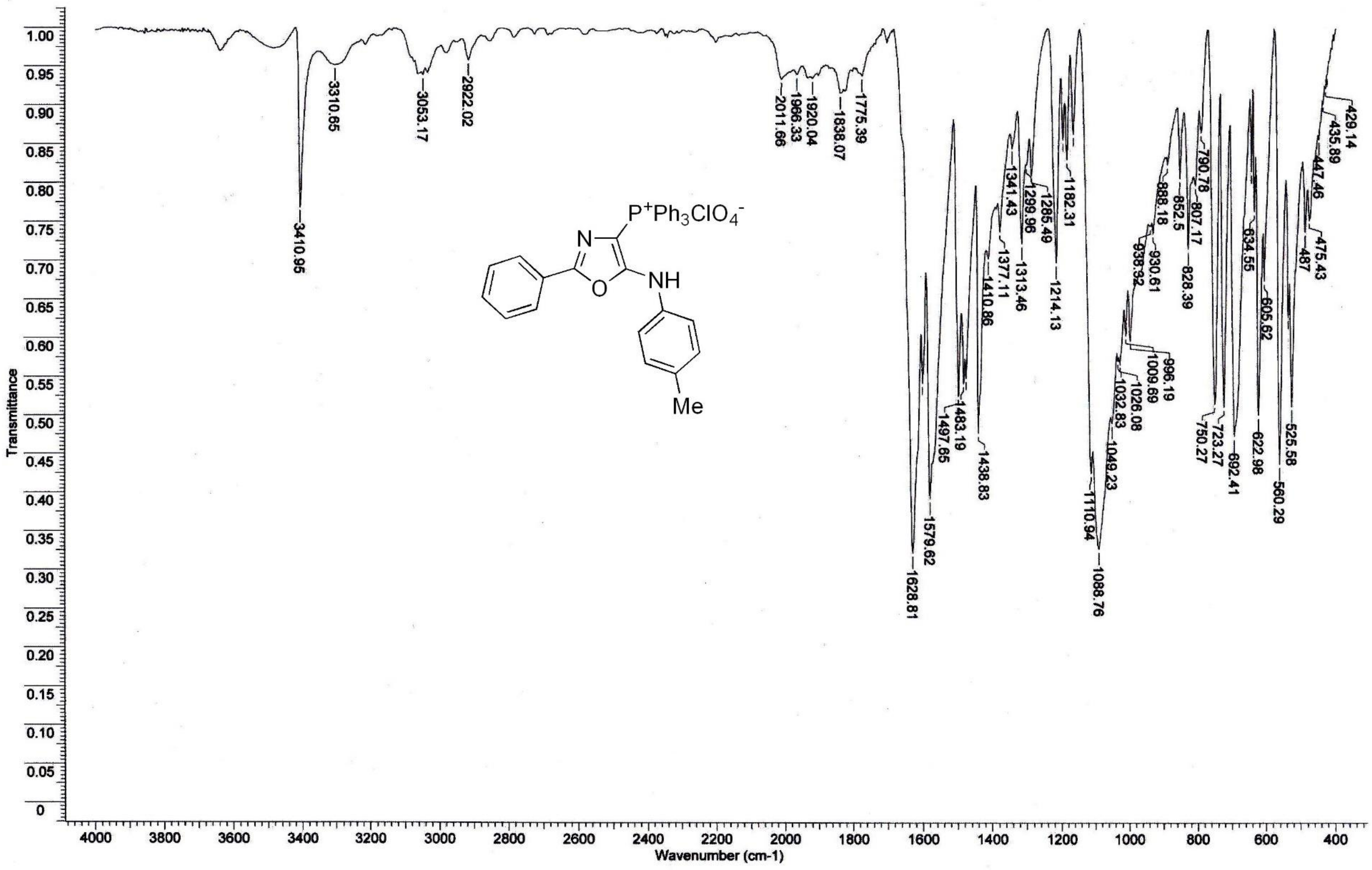

Figure S4. IR spectrum of compound (**1**).

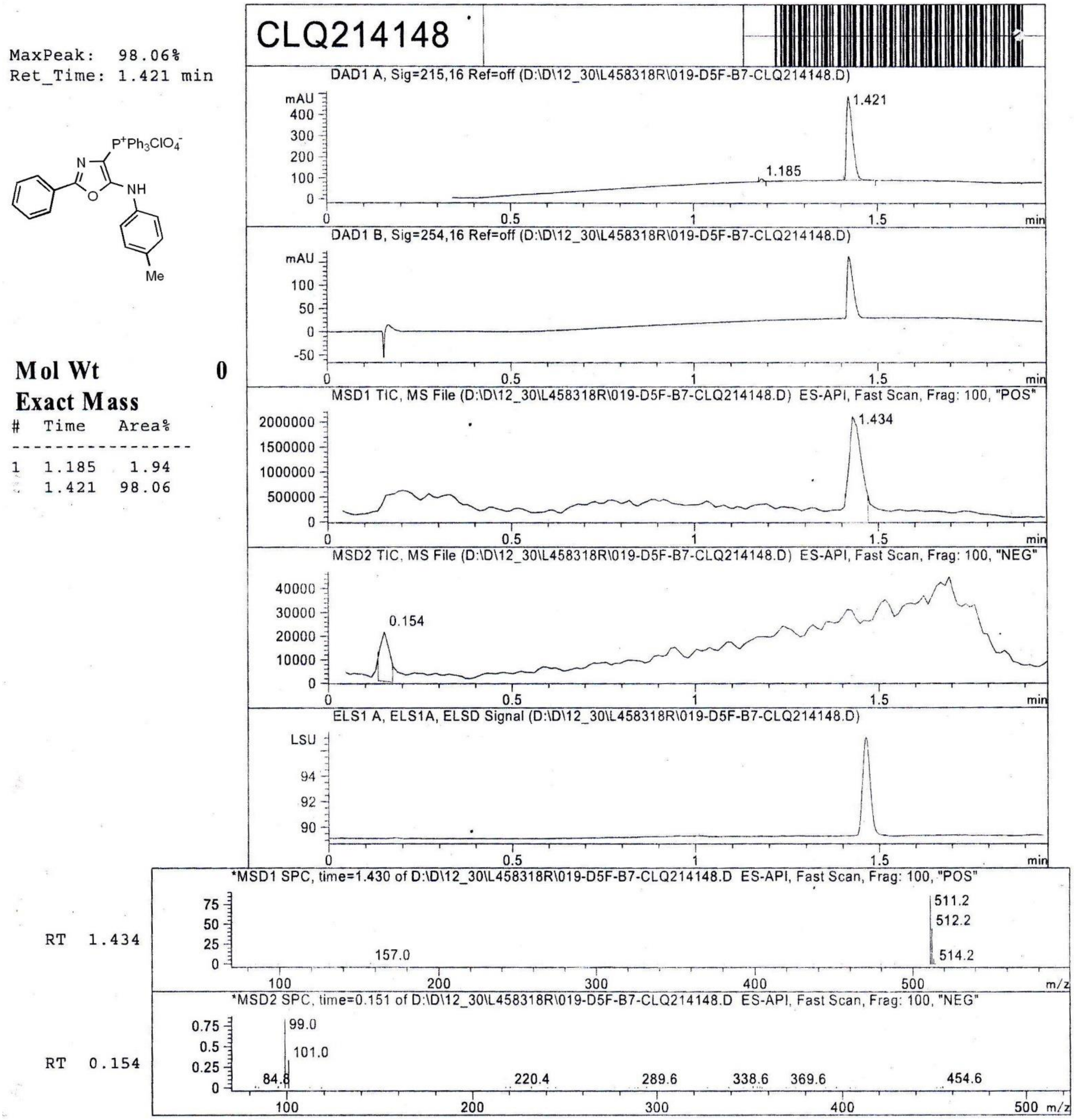

Figure S5. LCMS spectrum of compound (**1**).

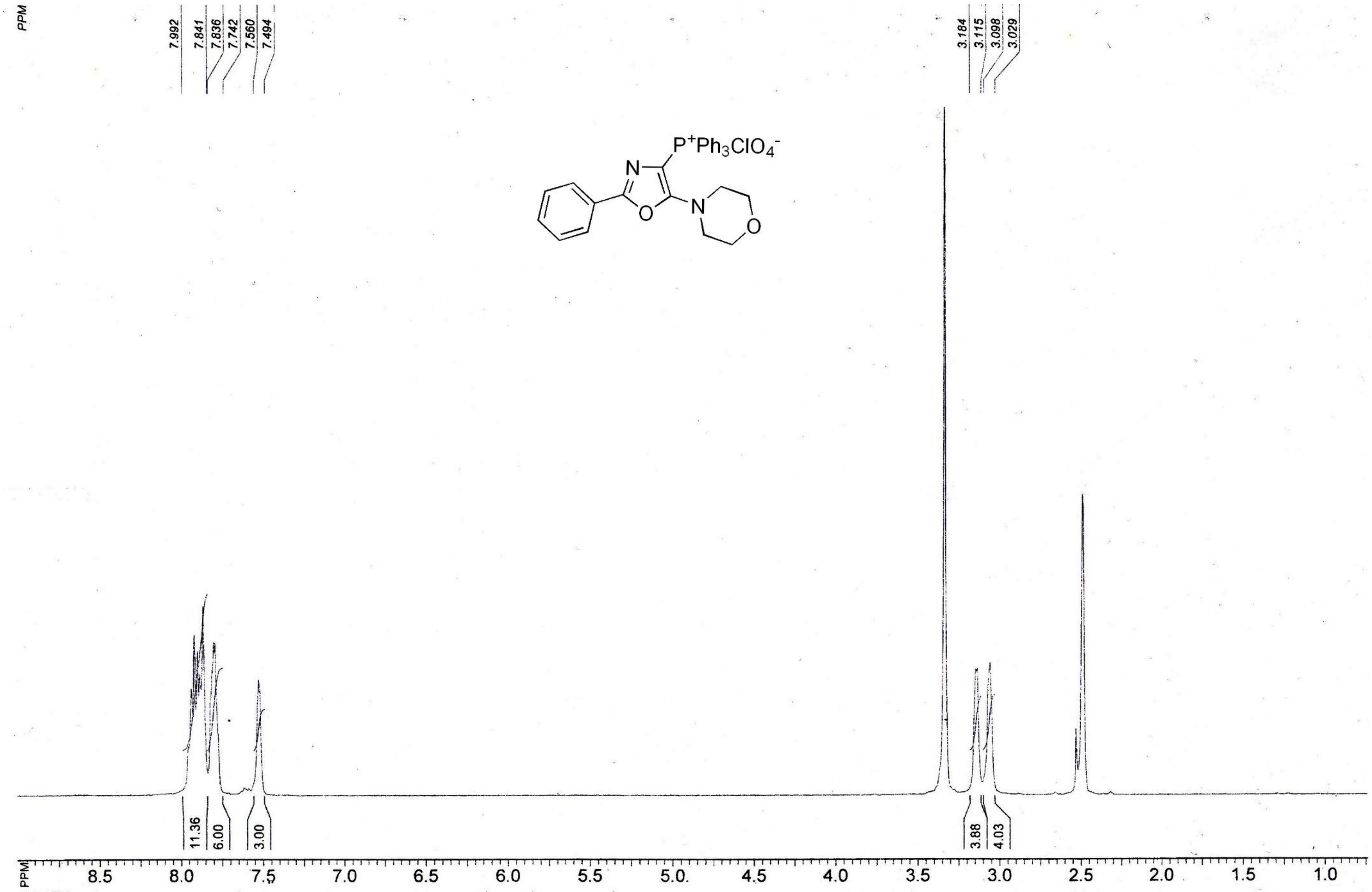

Figure S6. ^{1}H NMR (400 MHz, 294 K, DMSO-d_6) spectrum of compound (**2**).

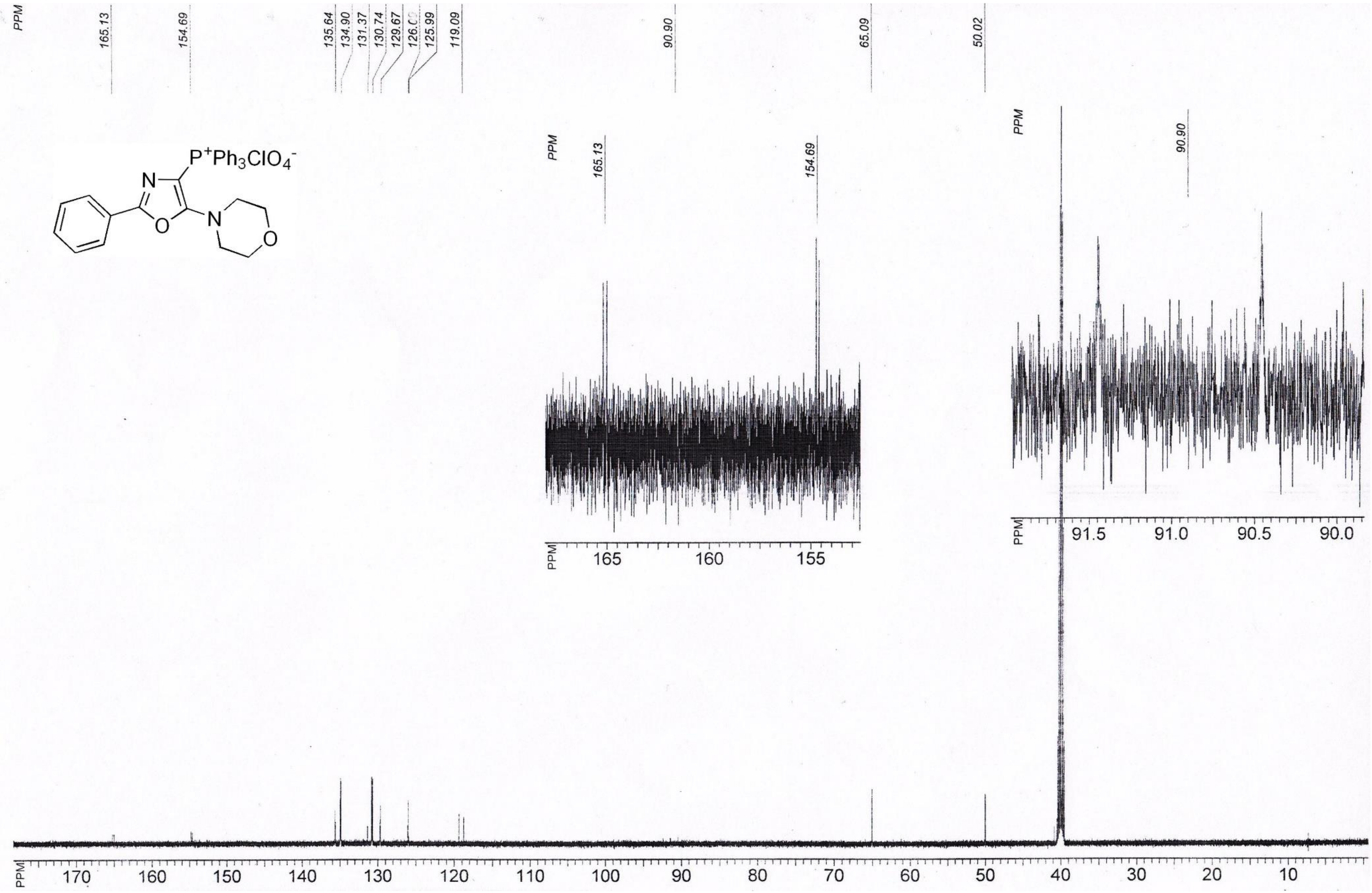

Figure S7. ^{13}C NMR (151 MHz, 298 K, DMSO-d_6) spectrum of compound (**2**).

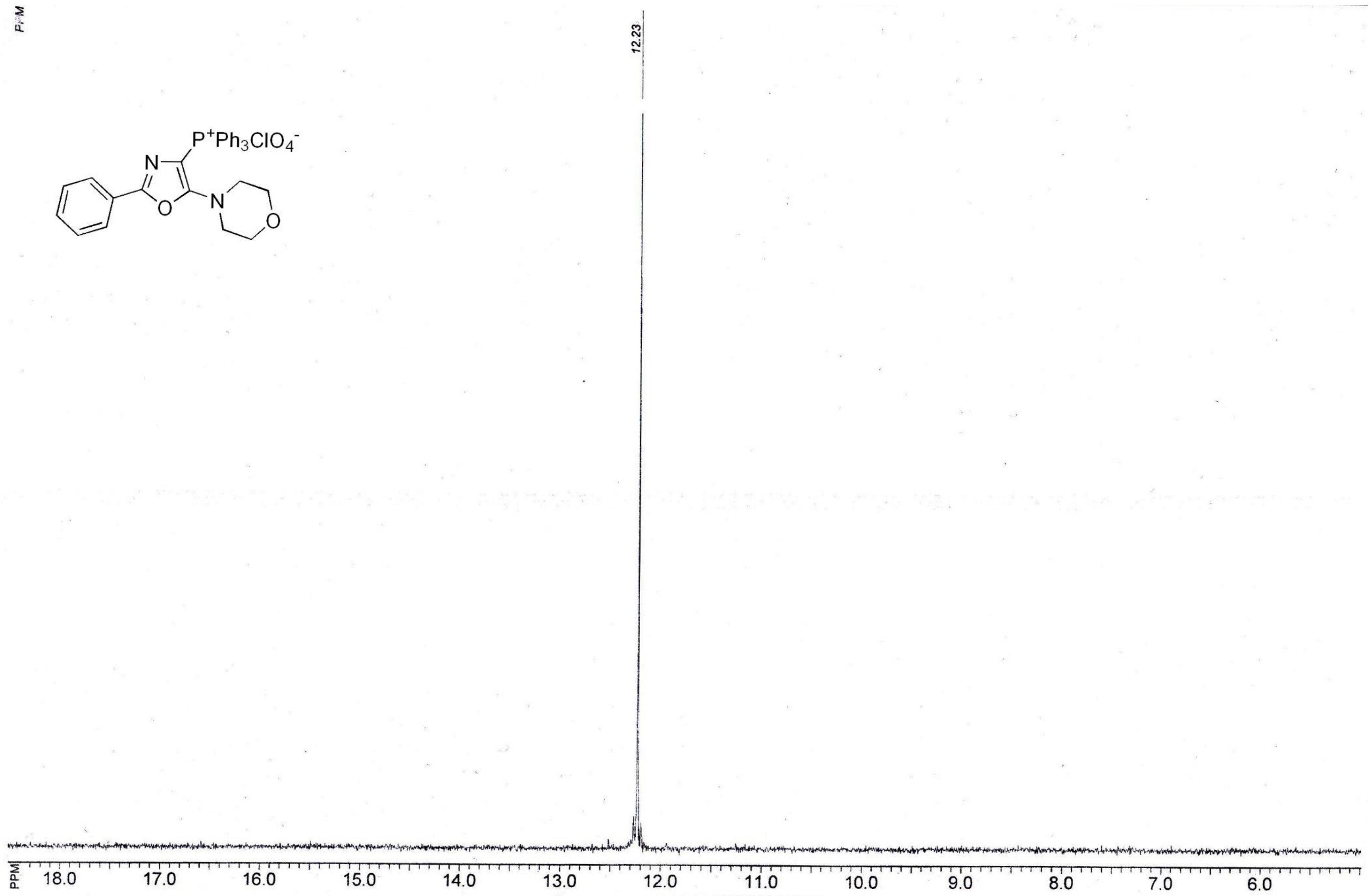

Figure S8. ^{31}P NMR (162 MHz, 294 K, DMSO-d_6) spectrum of compound (**2**).

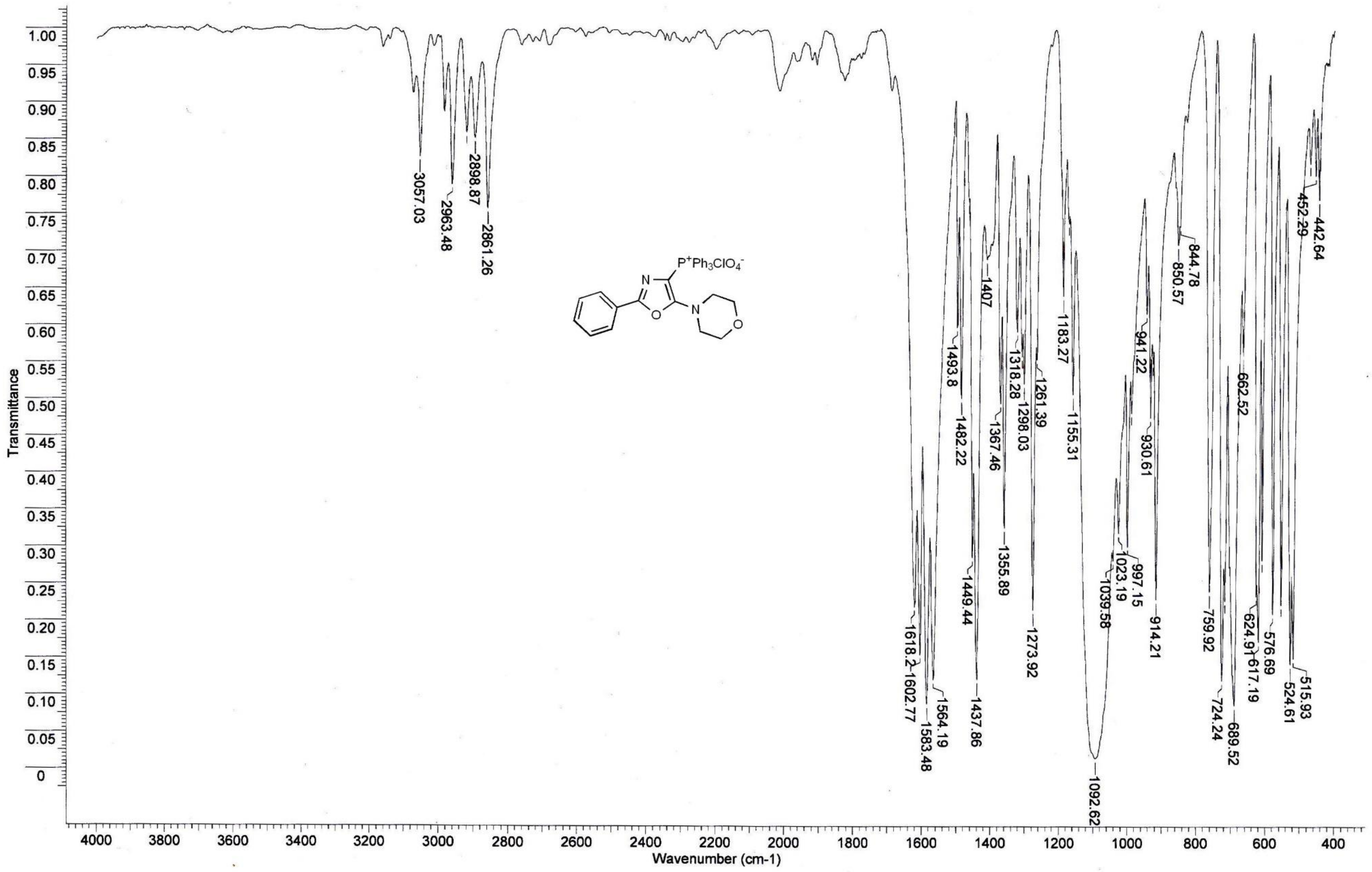

Figure S9. IR spectrum of compound (**2**).

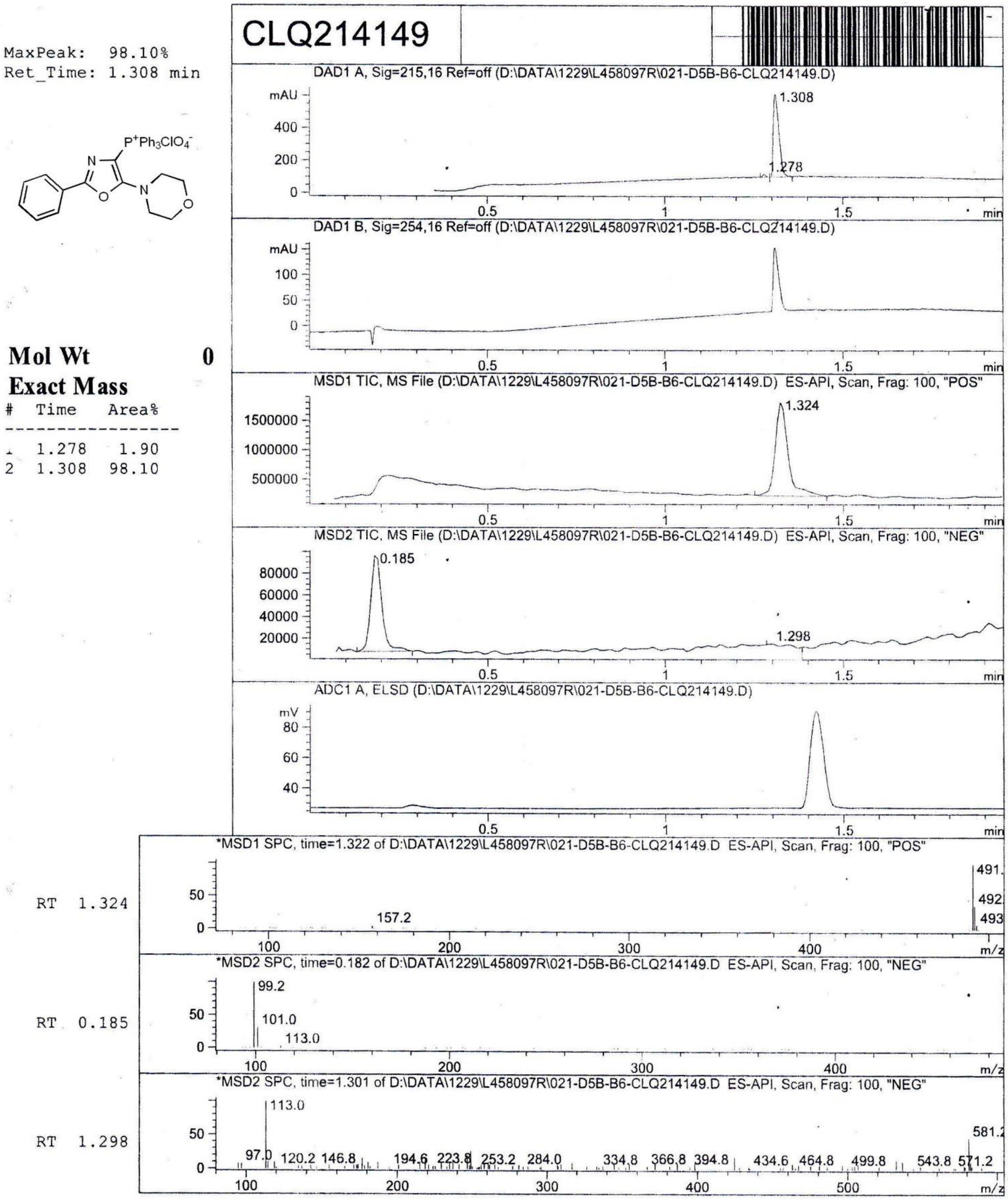

Figure S10. LCMS spectrum of compound (**2**).

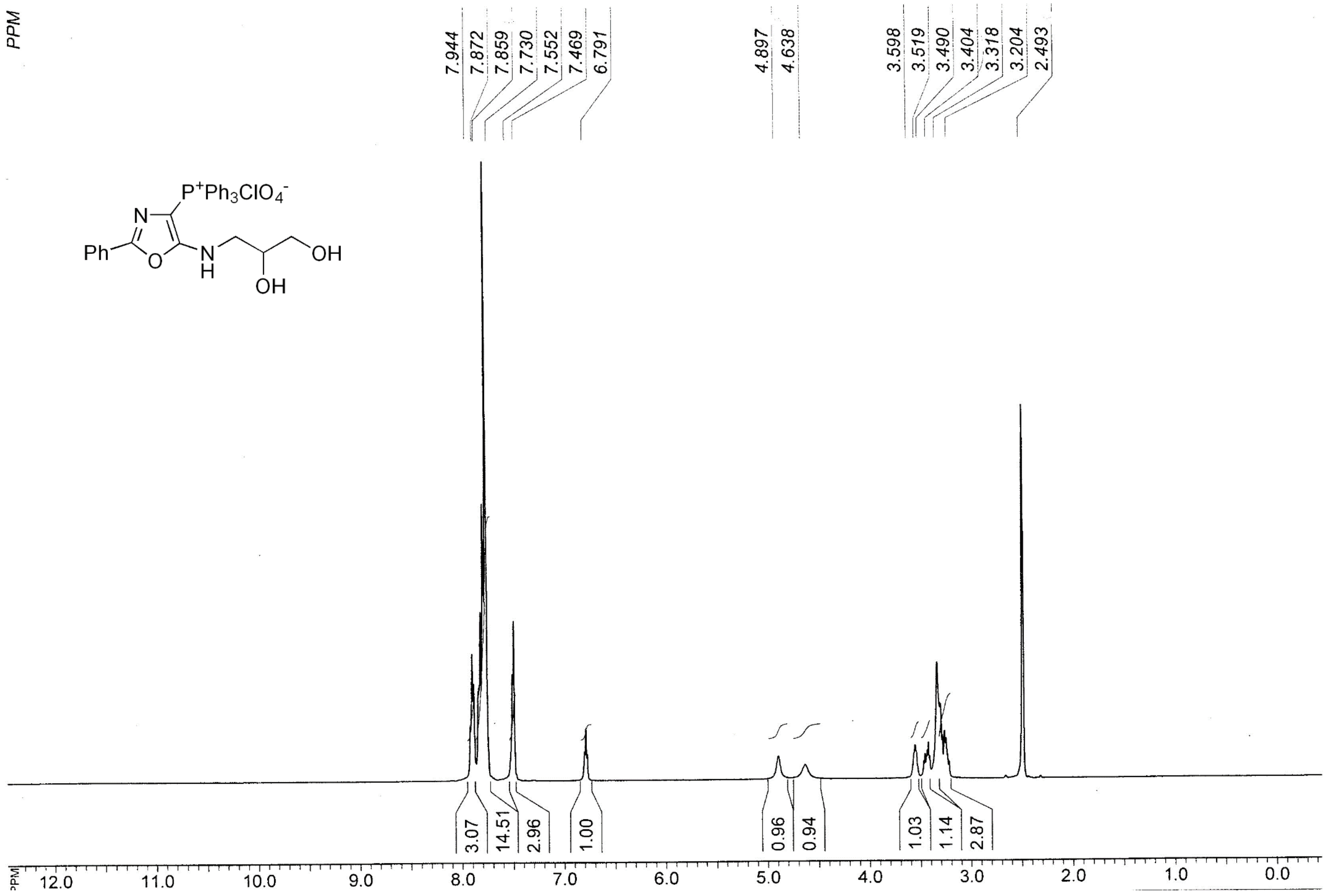

Figure S11. ^{1}H NMR (400 MHz, 294 K, DMSO-d_6) spectrum of compound (**3**).

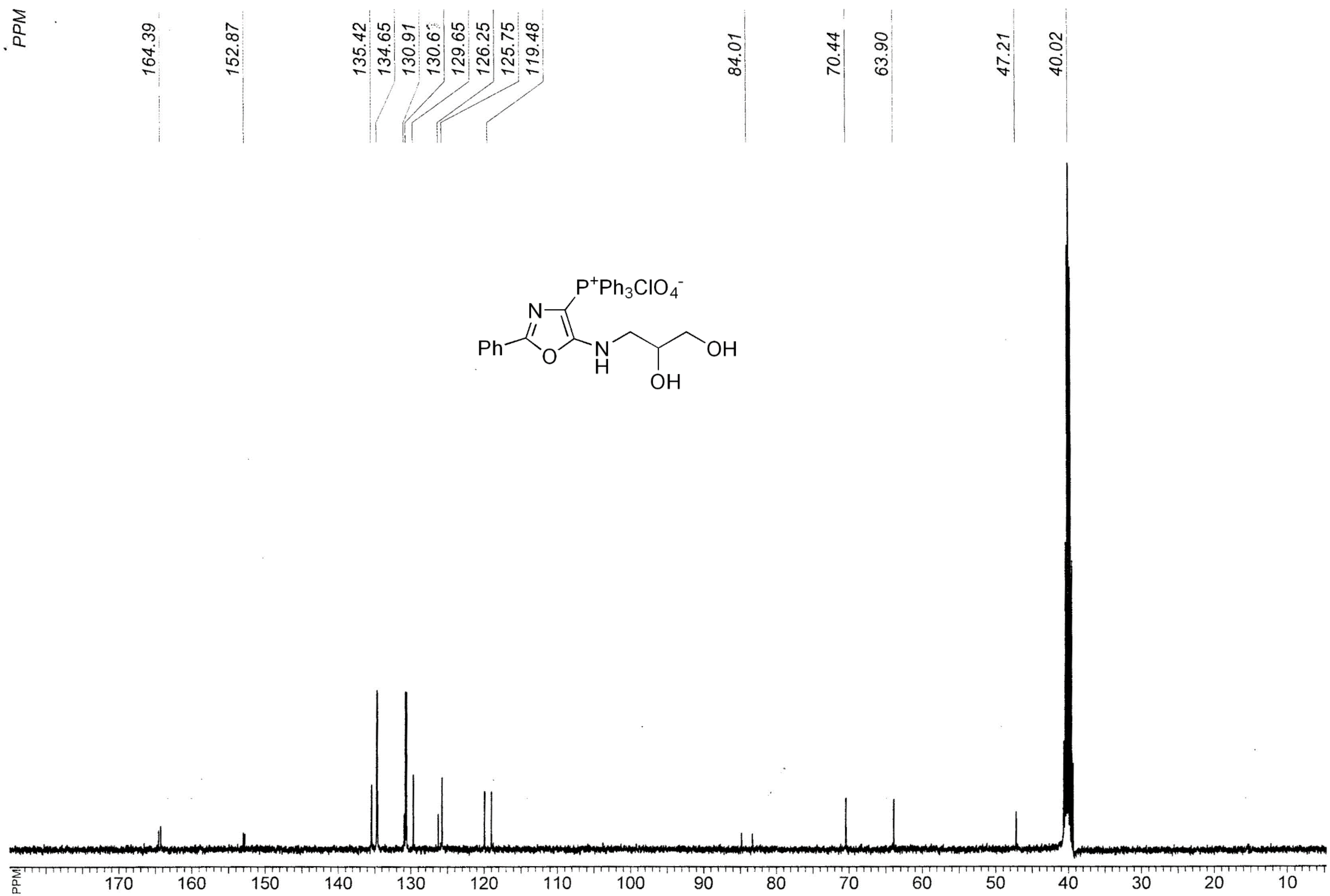

Figure S12. ^{13}C NMR (126 MHz, 298 K, DMSO-d_6) spectrum of compound (**3**).

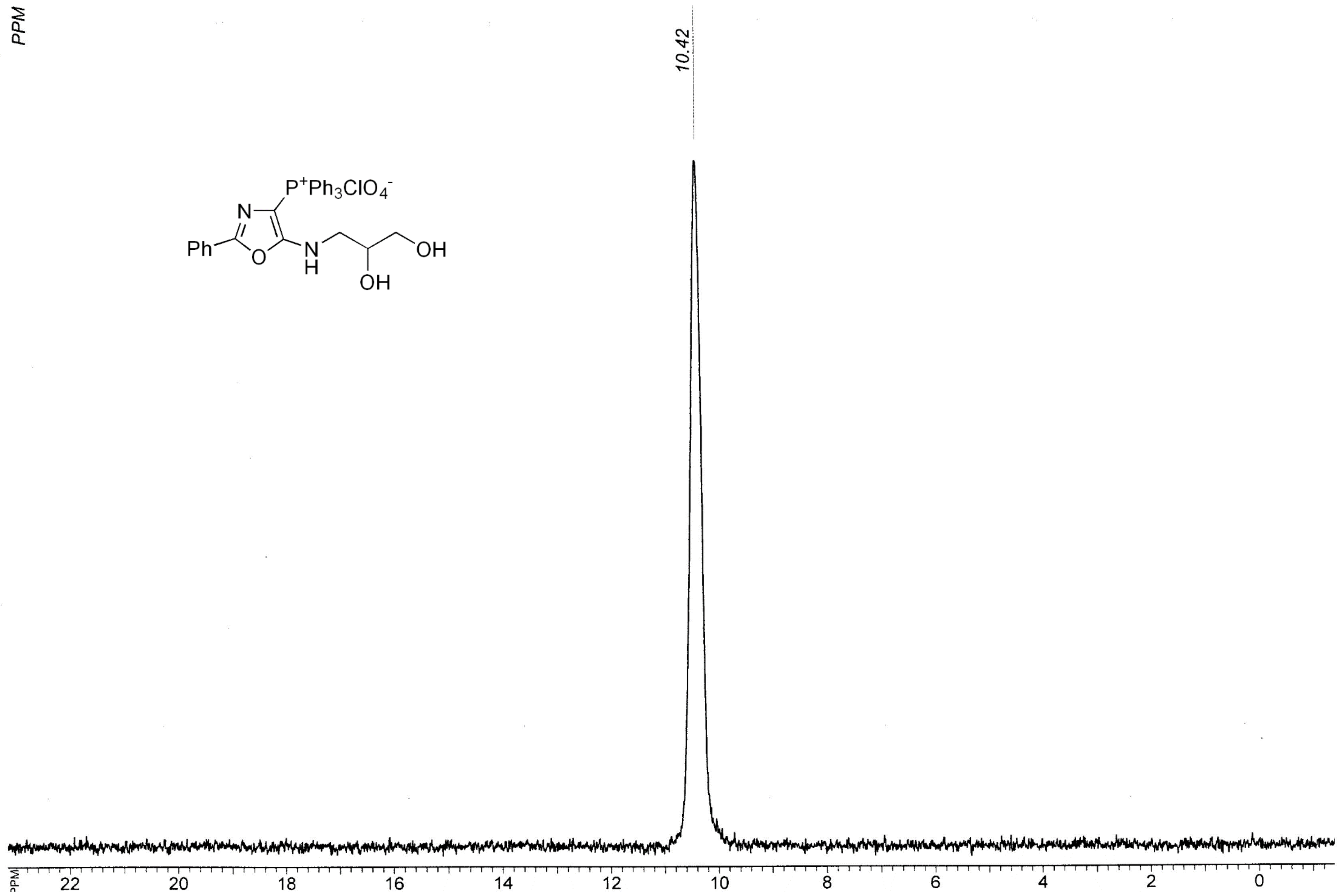

Figure S13. ^{31}P NMR (162 MHz, 294 K, DMSO-*d₆*) spectrum of compound (**3**).

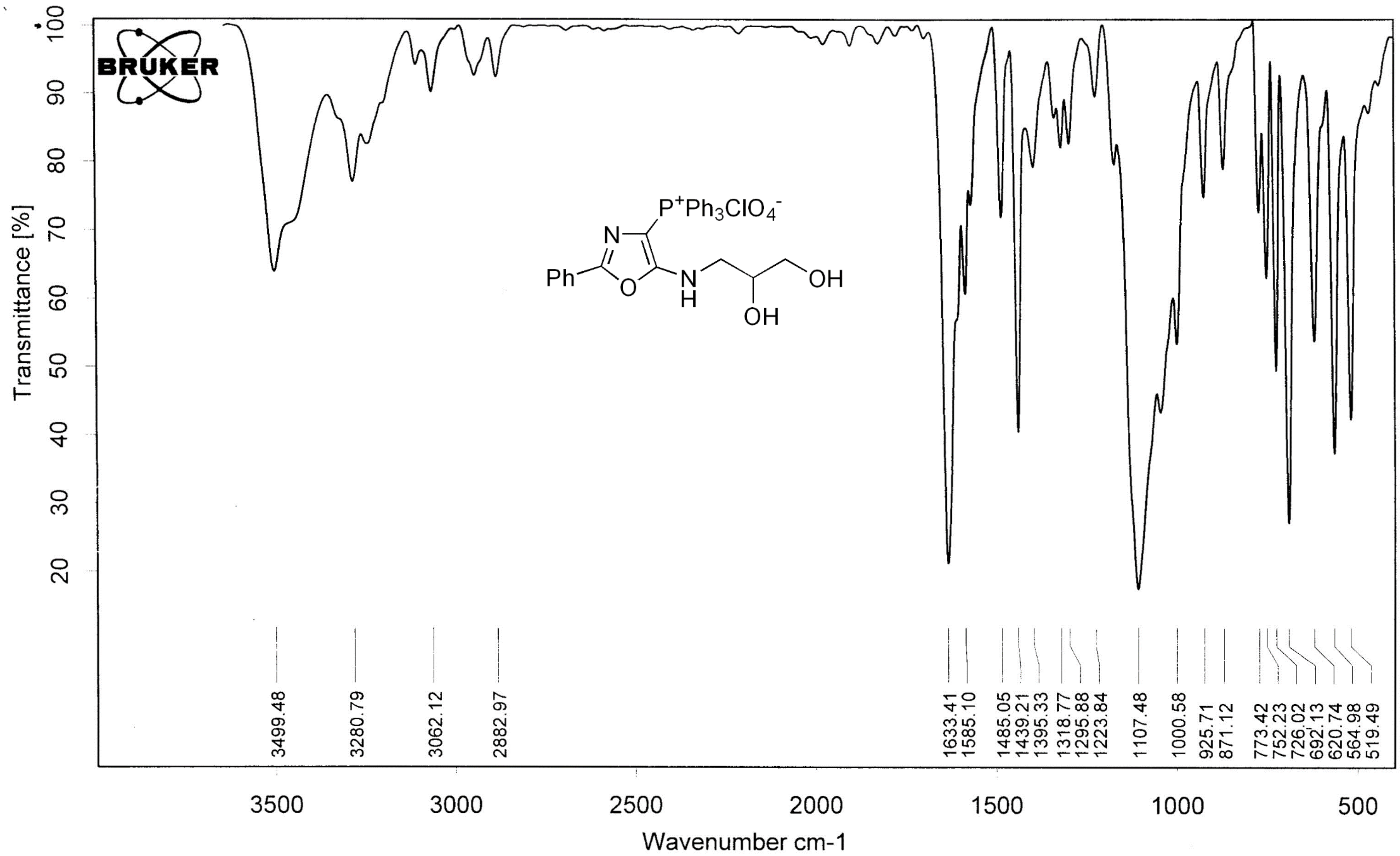

Figure S14. IR spectrum of compound (**3**).

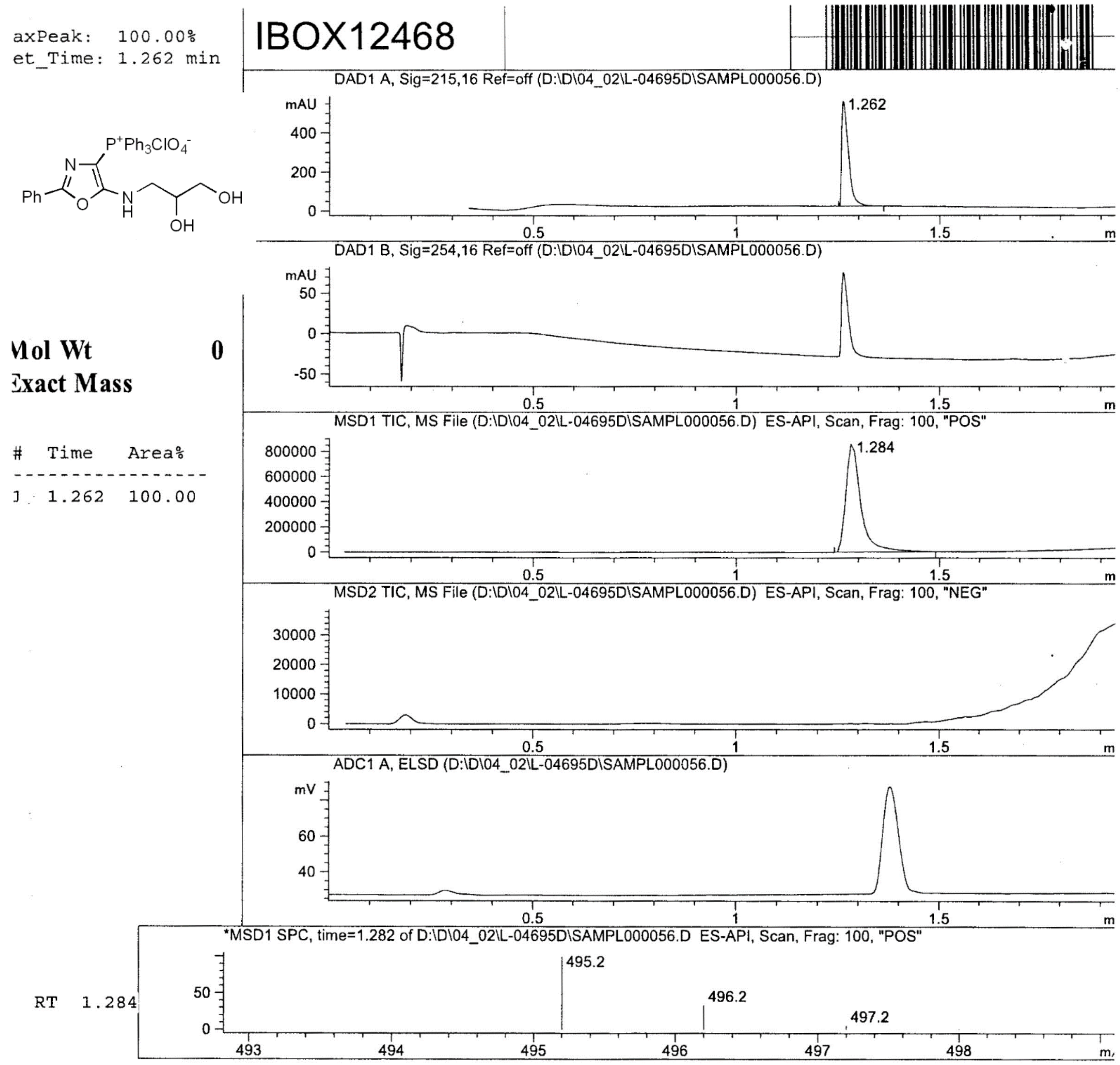

Figure S15. LCMS spectrum of compound (**3**).

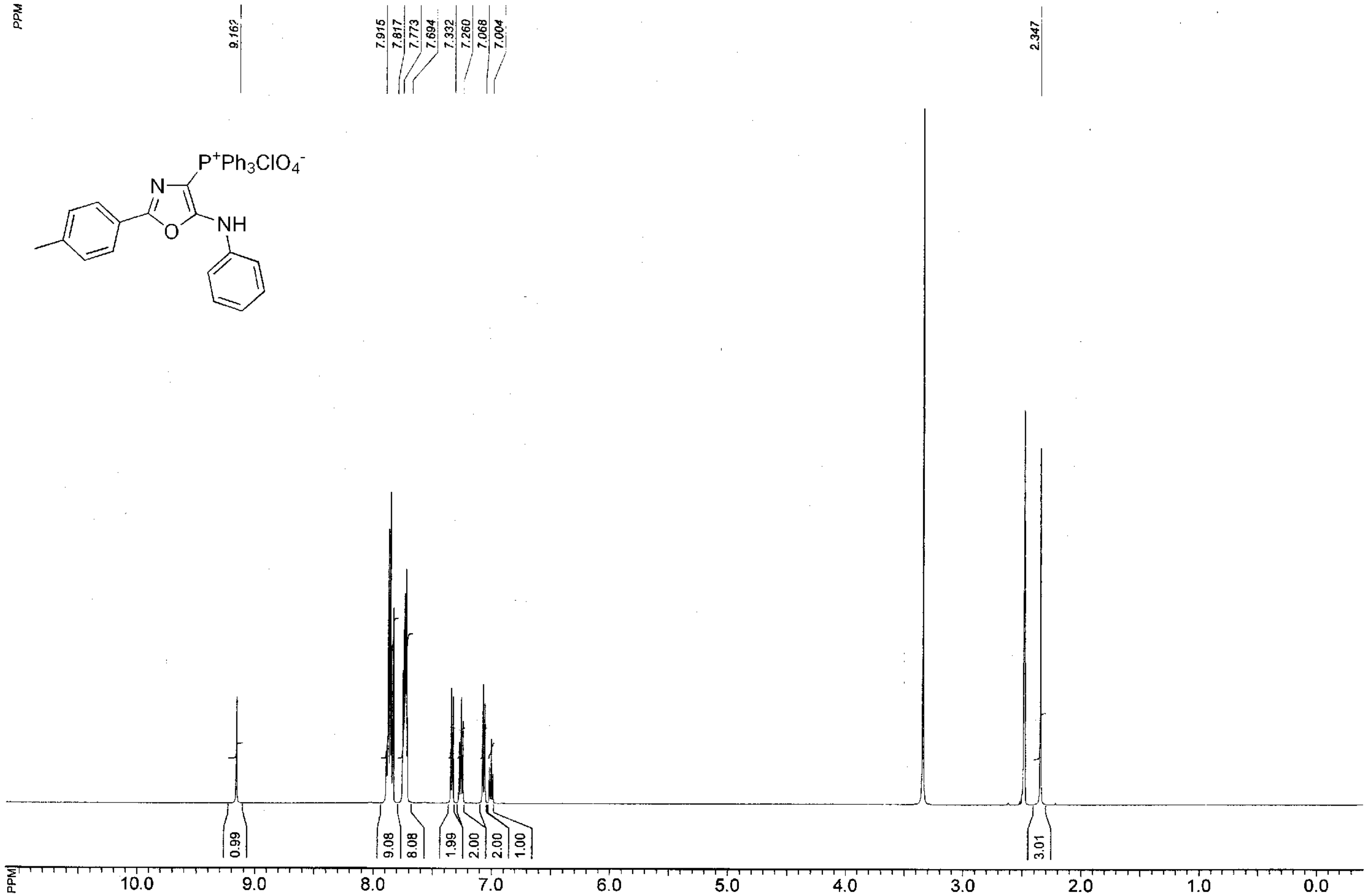

Figure S16. ^{1}H NMR (400 MHz, 294 K, DMSO-d_6) spectrum of compound (**4**).

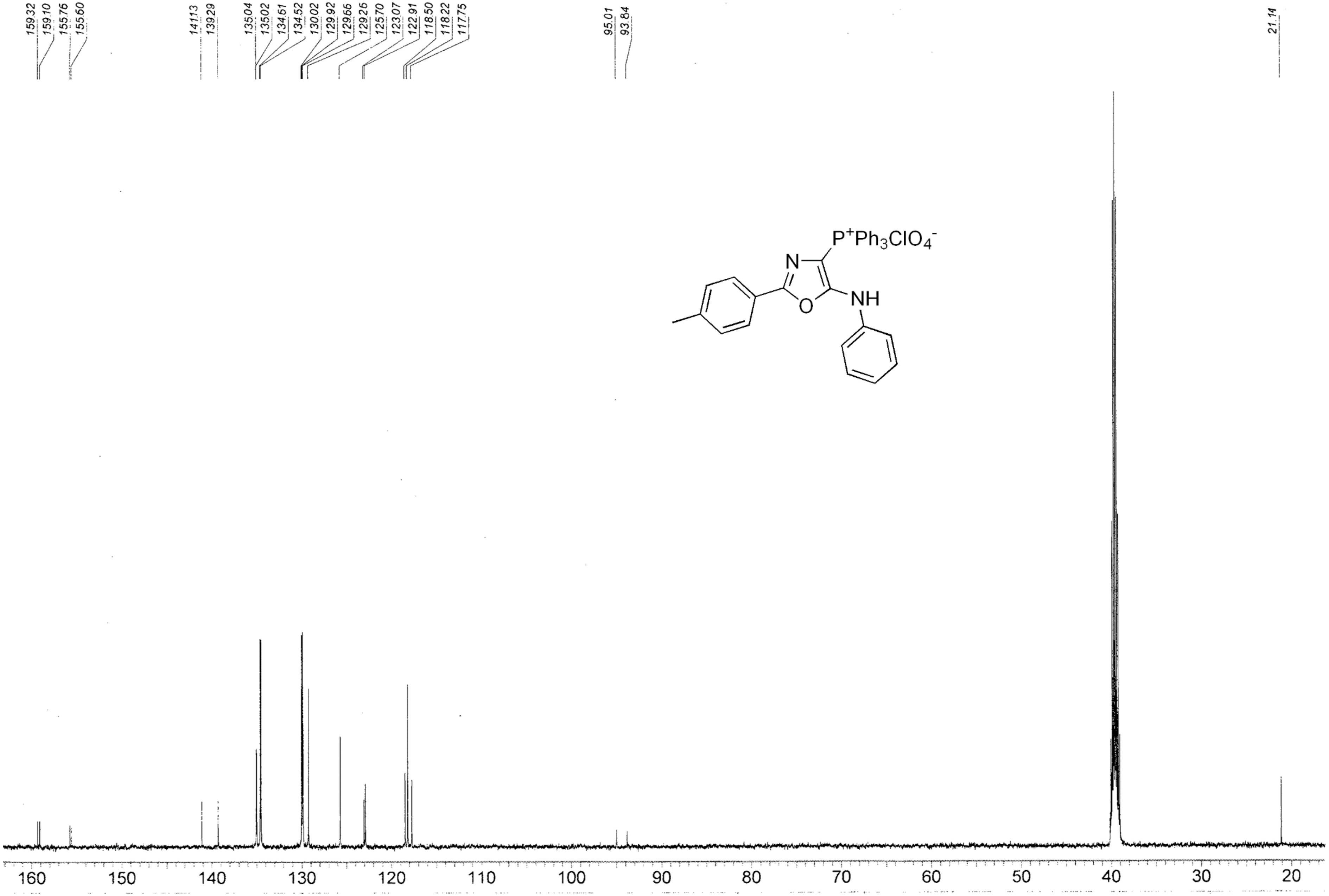

Figure S17. ^{13}C NMR (126 MHz, 298 K, DMSO-d_6) spectrum of compound (**4**).

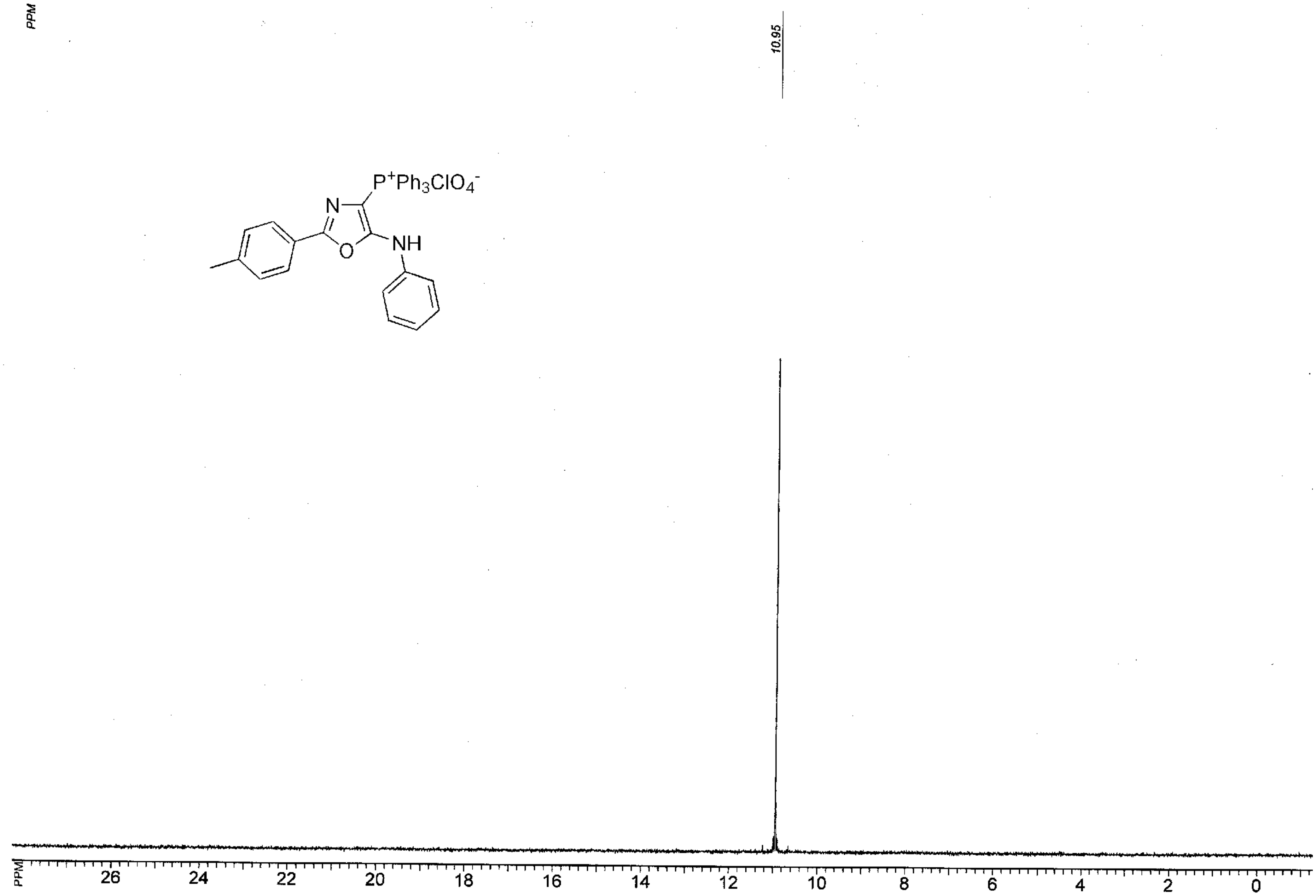

Figure S18. ^{31}P NMR (162 MHz, 294 K, DMSO-d_6) spectrum of compound (**4**).

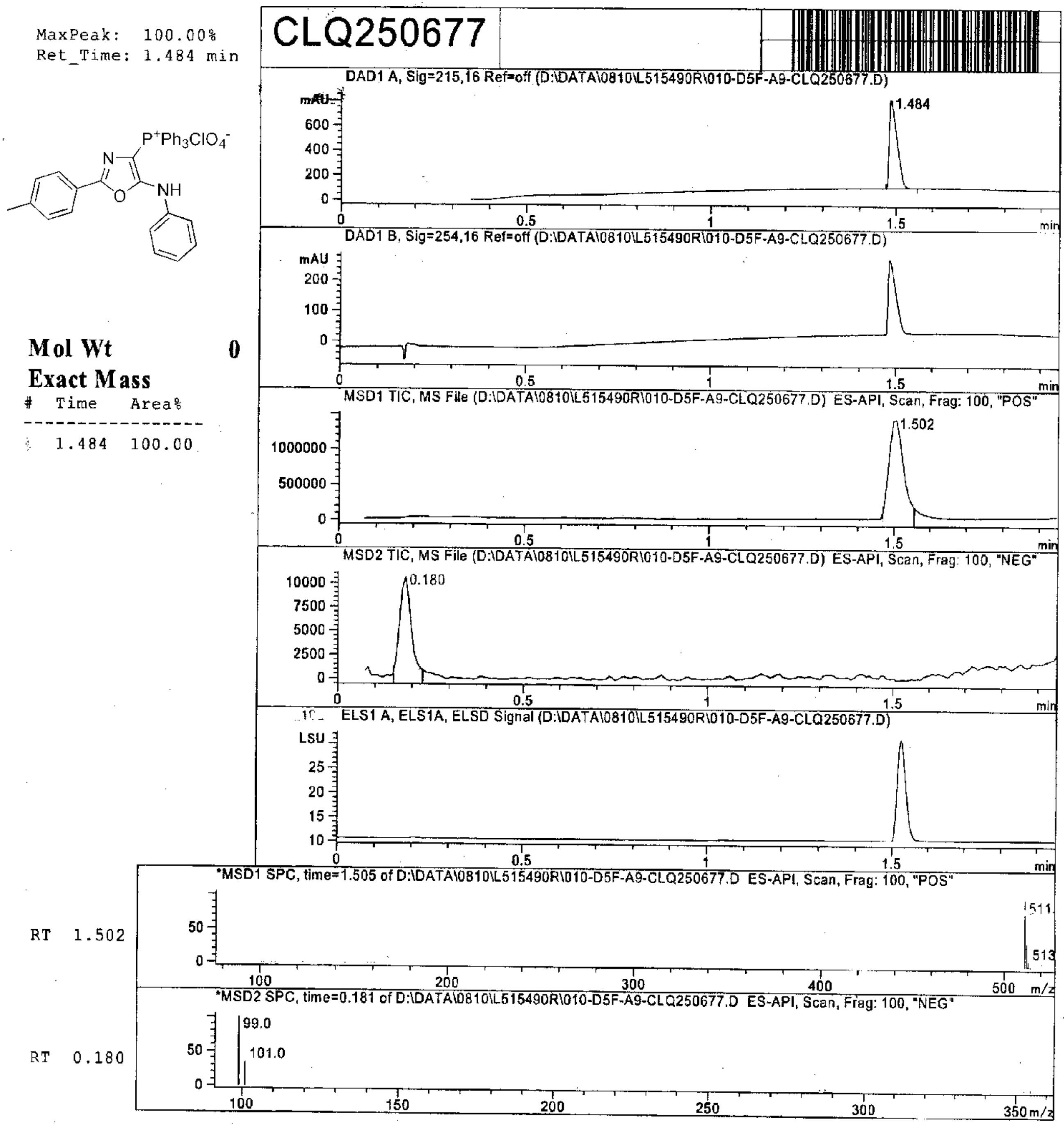

Figure S19. LCMS spectrum of compound (**4**).

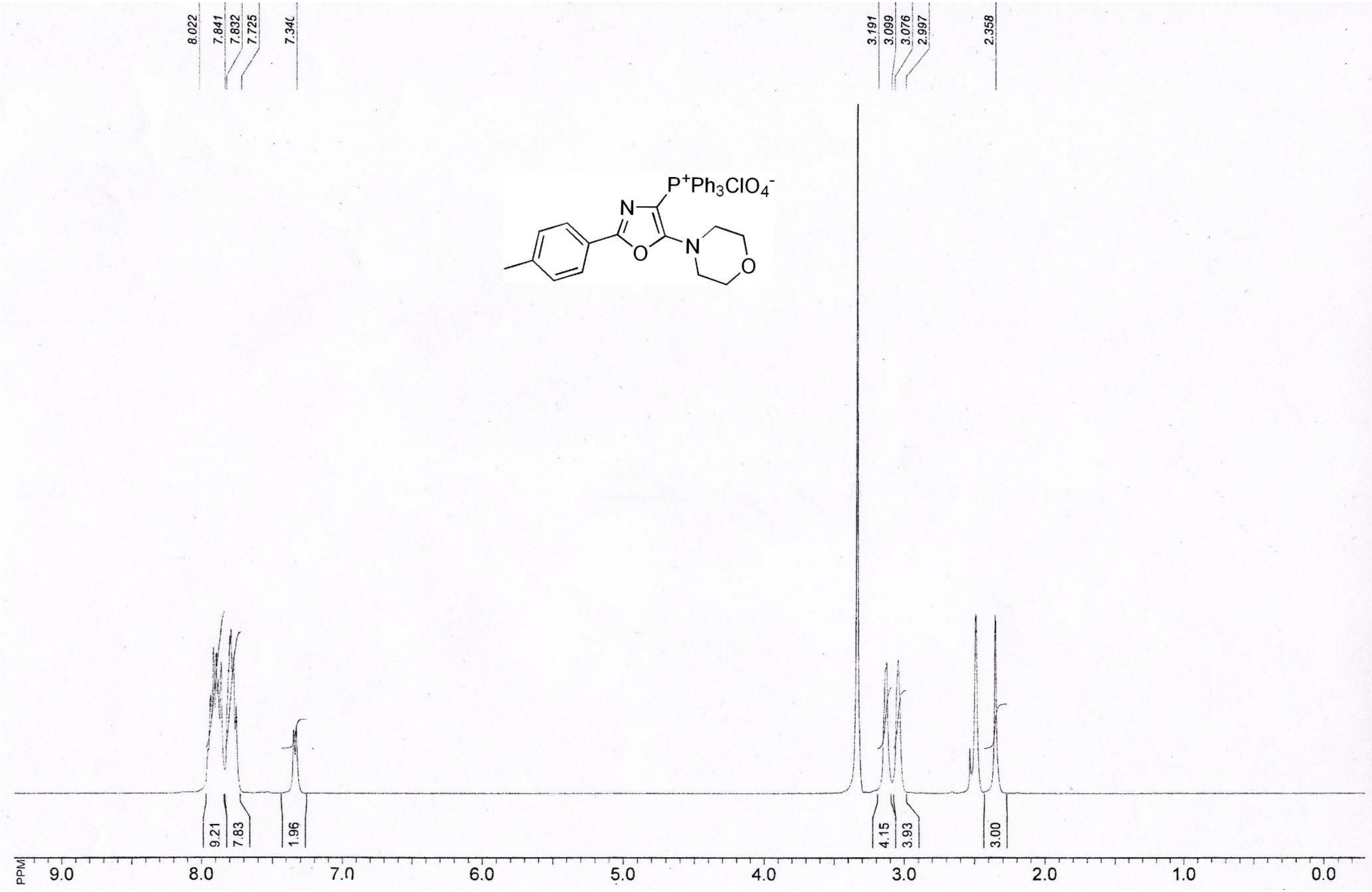

Figure S20. ^{1}H NMR (400 MHz, 294 K, DMSO-d_6) spectrum of compound (**5**).

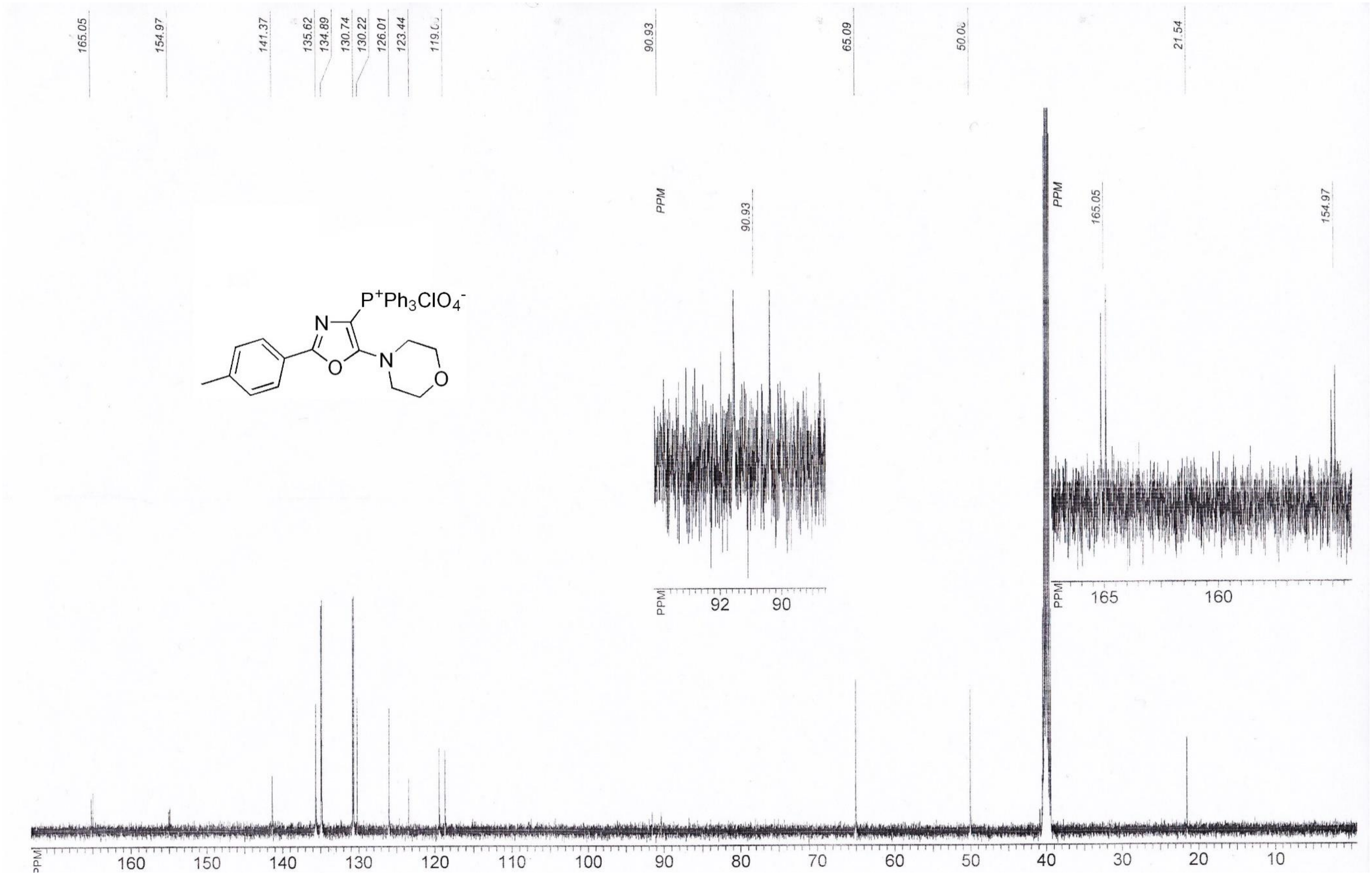

Figure S21. ^{13}C NMR (126 MHz, 298 K, DMSO-d_6) spectrum of compound (**5**).

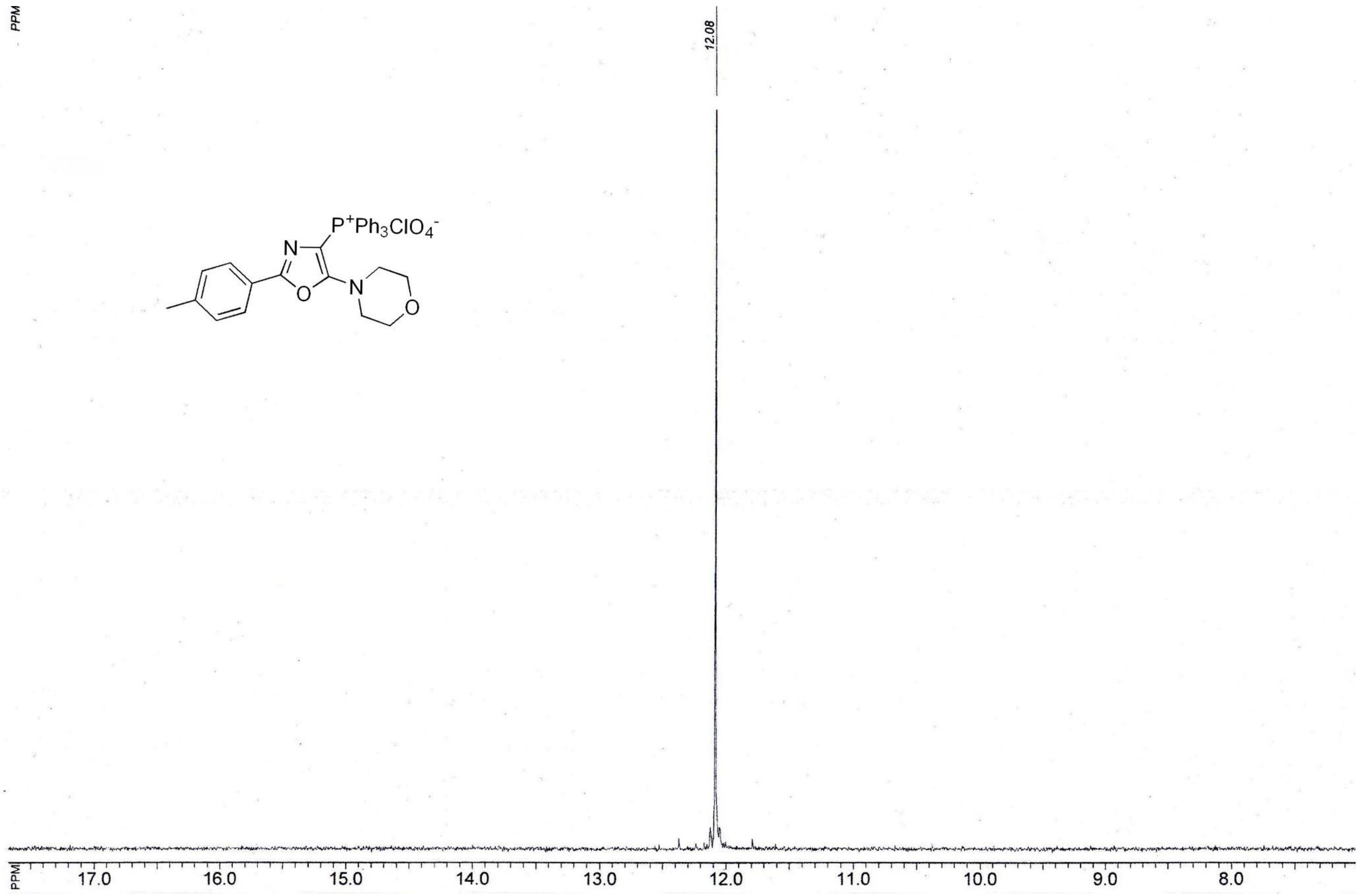

Figure S22. ^{31}P NMR (162 MHz, 294 K, DMSO-d_6) spectrum of compound (**5**).

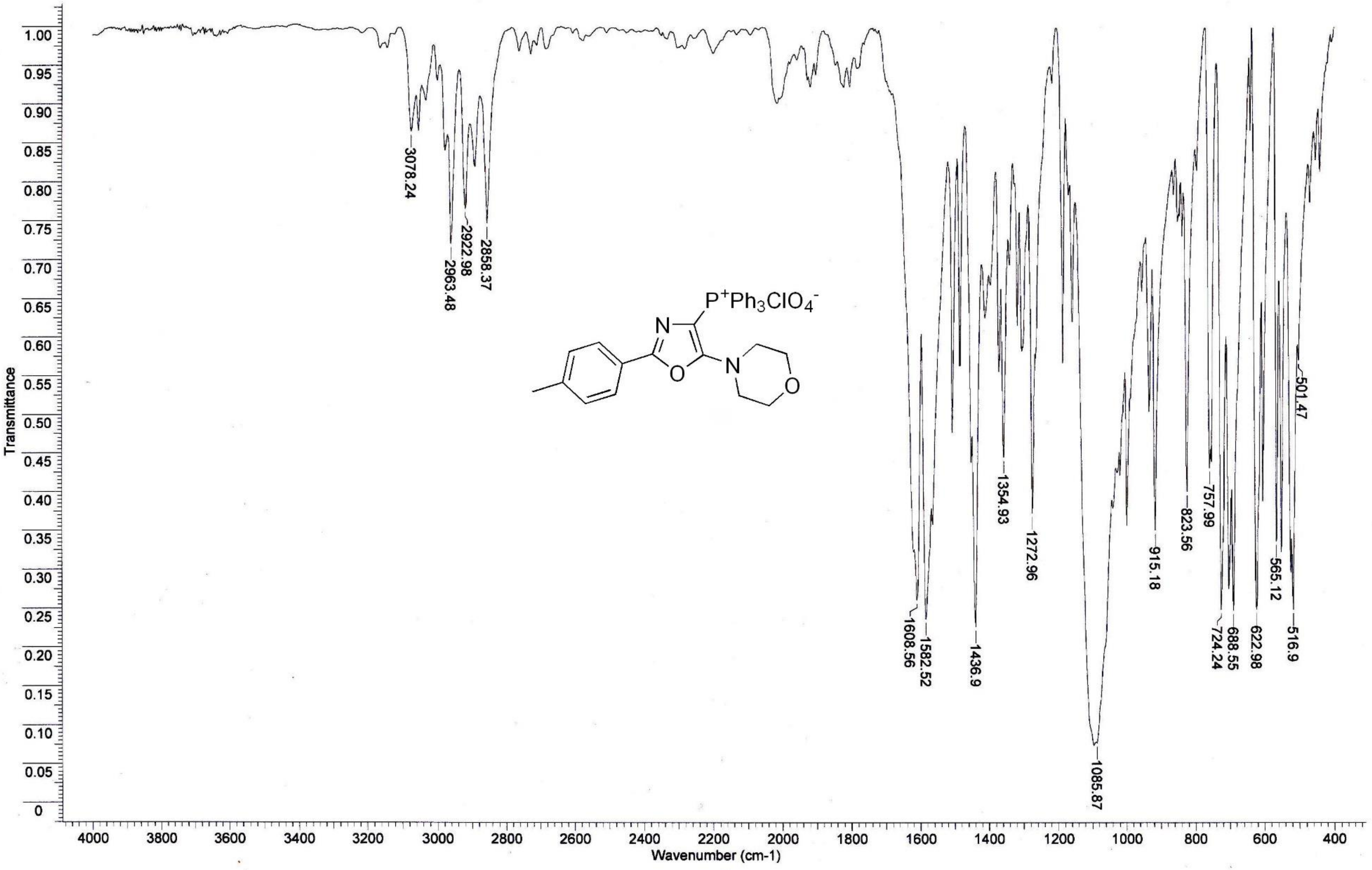

Figure S23. IR spectrum of compound (**5**).

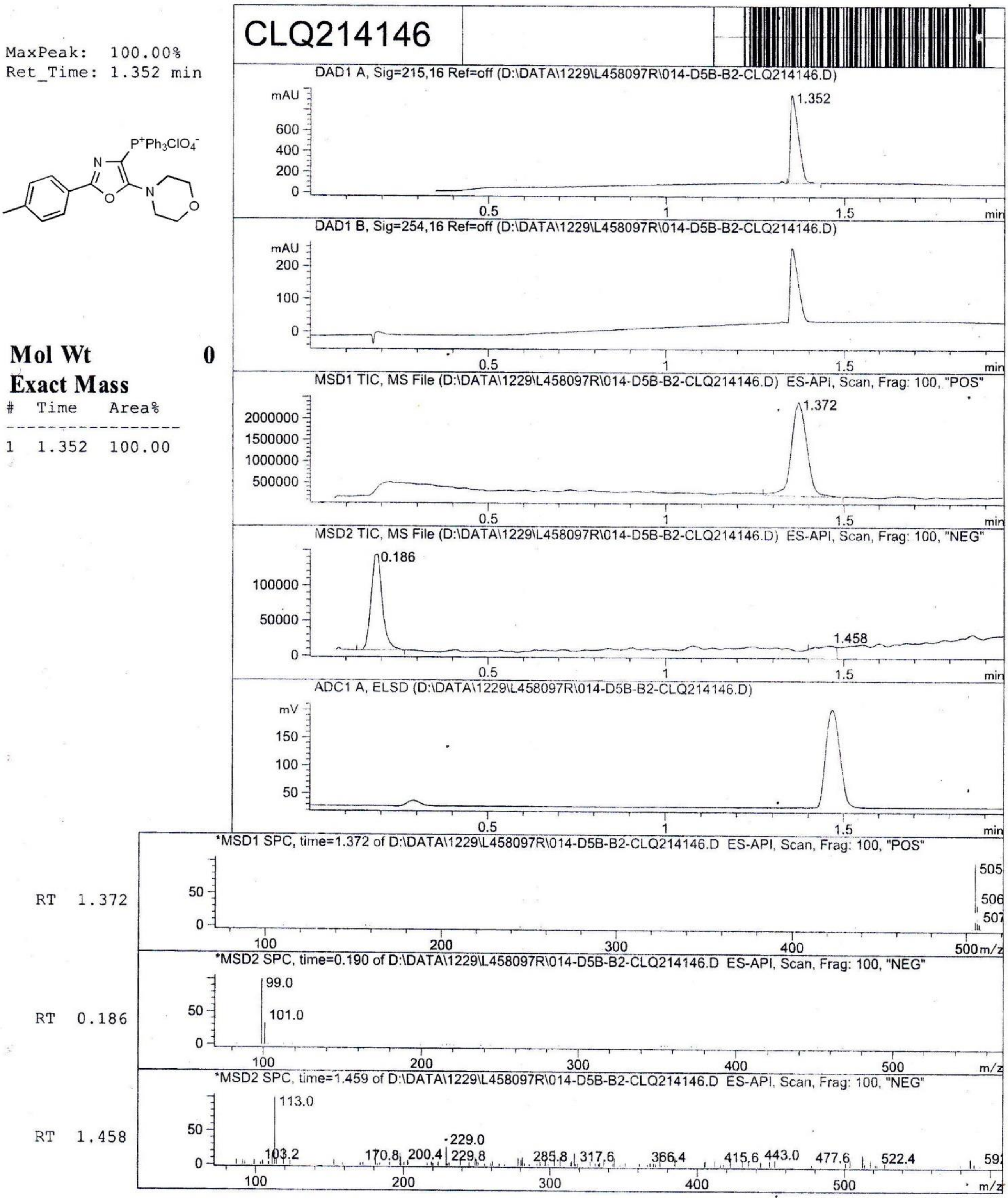

Figure S24. LCMS spectrum of compound (**5**).

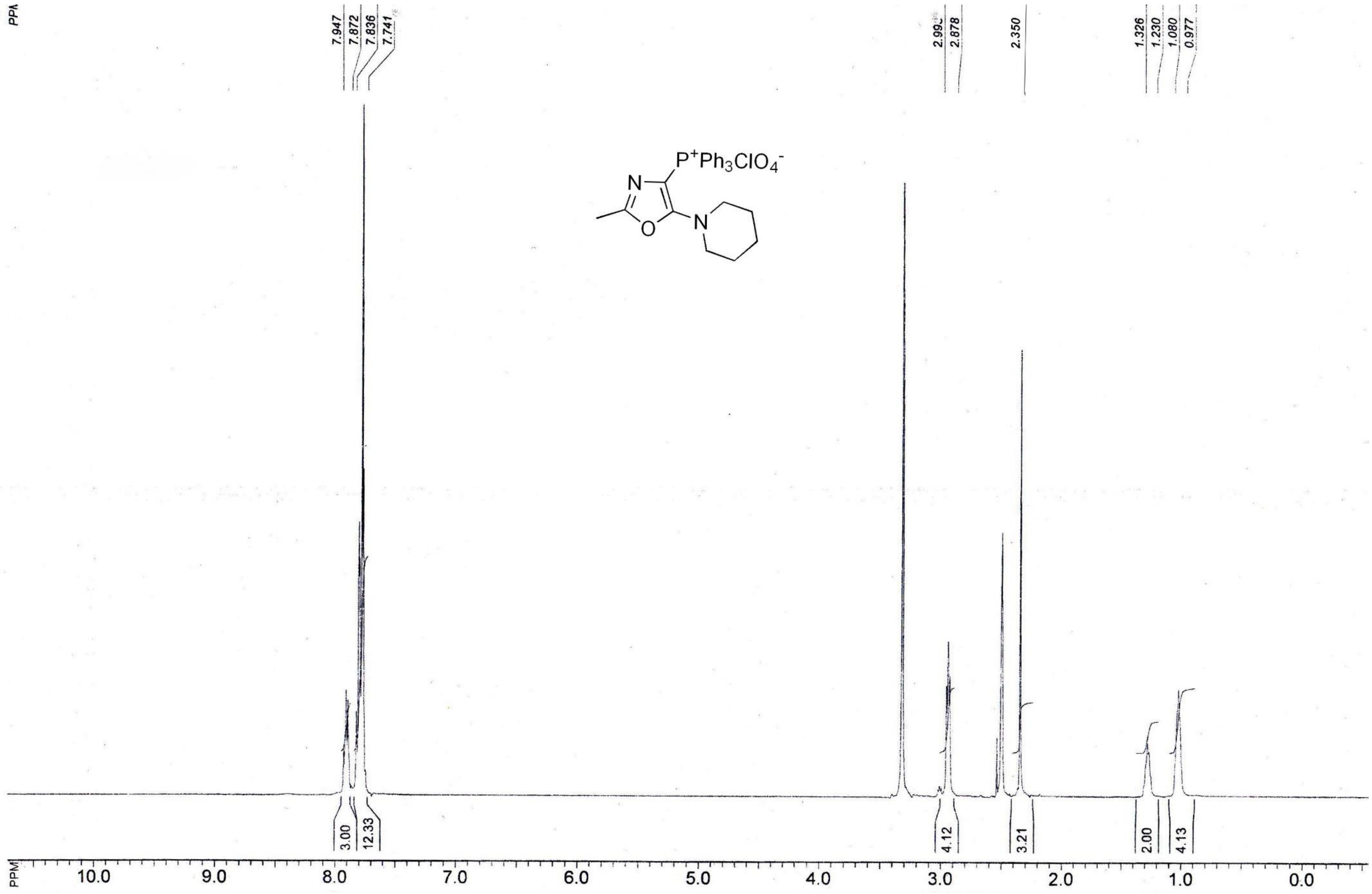

Figure S25. ^{1}H NMR (400 MHz, 298 K, DMSO-d_6) spectrum of compound **(6).**

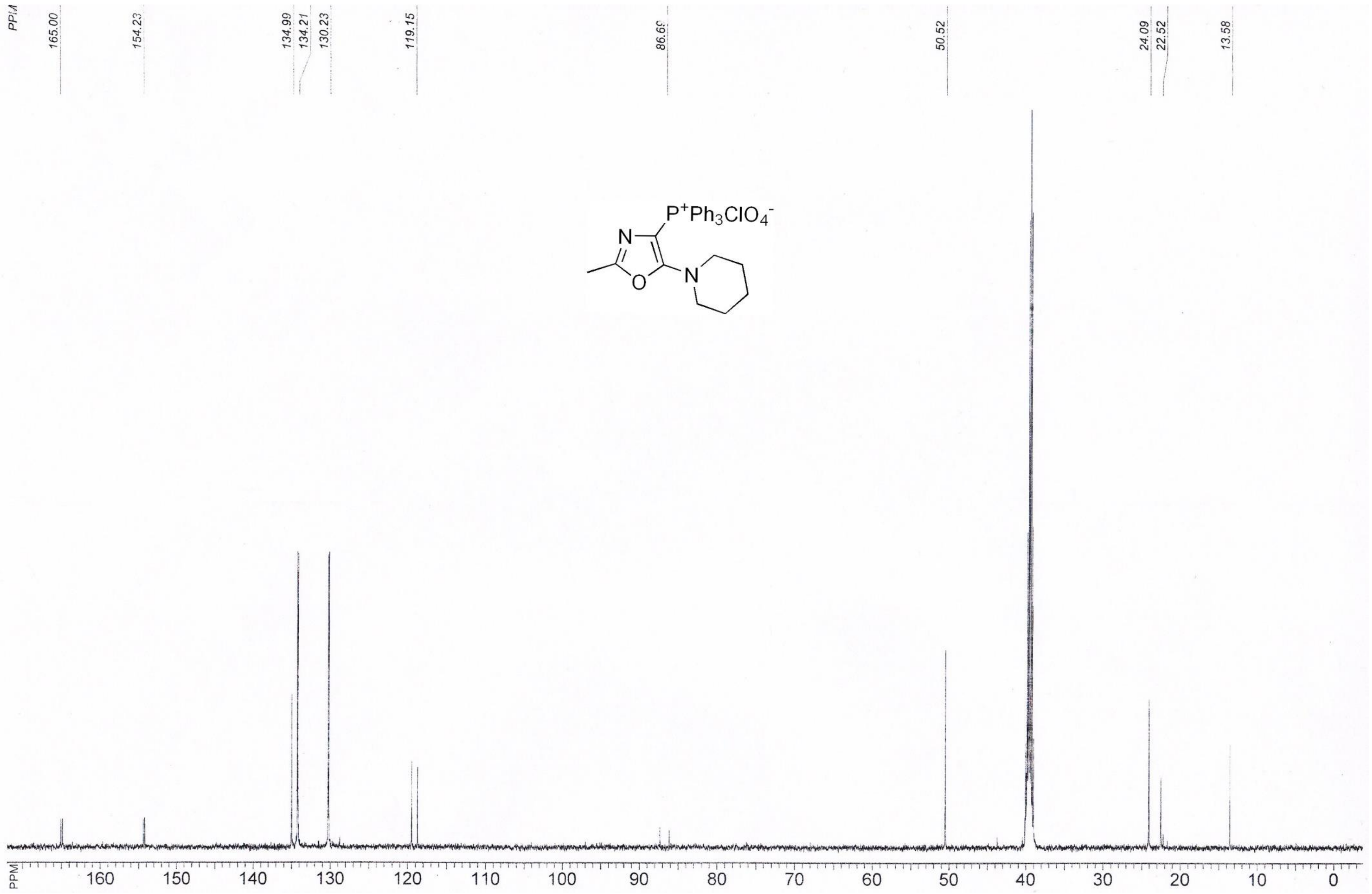

Figure S26. ^{13}C NMR (126 MHz, 683 K, DMSO-d_6) spectrum of compound **(6).**

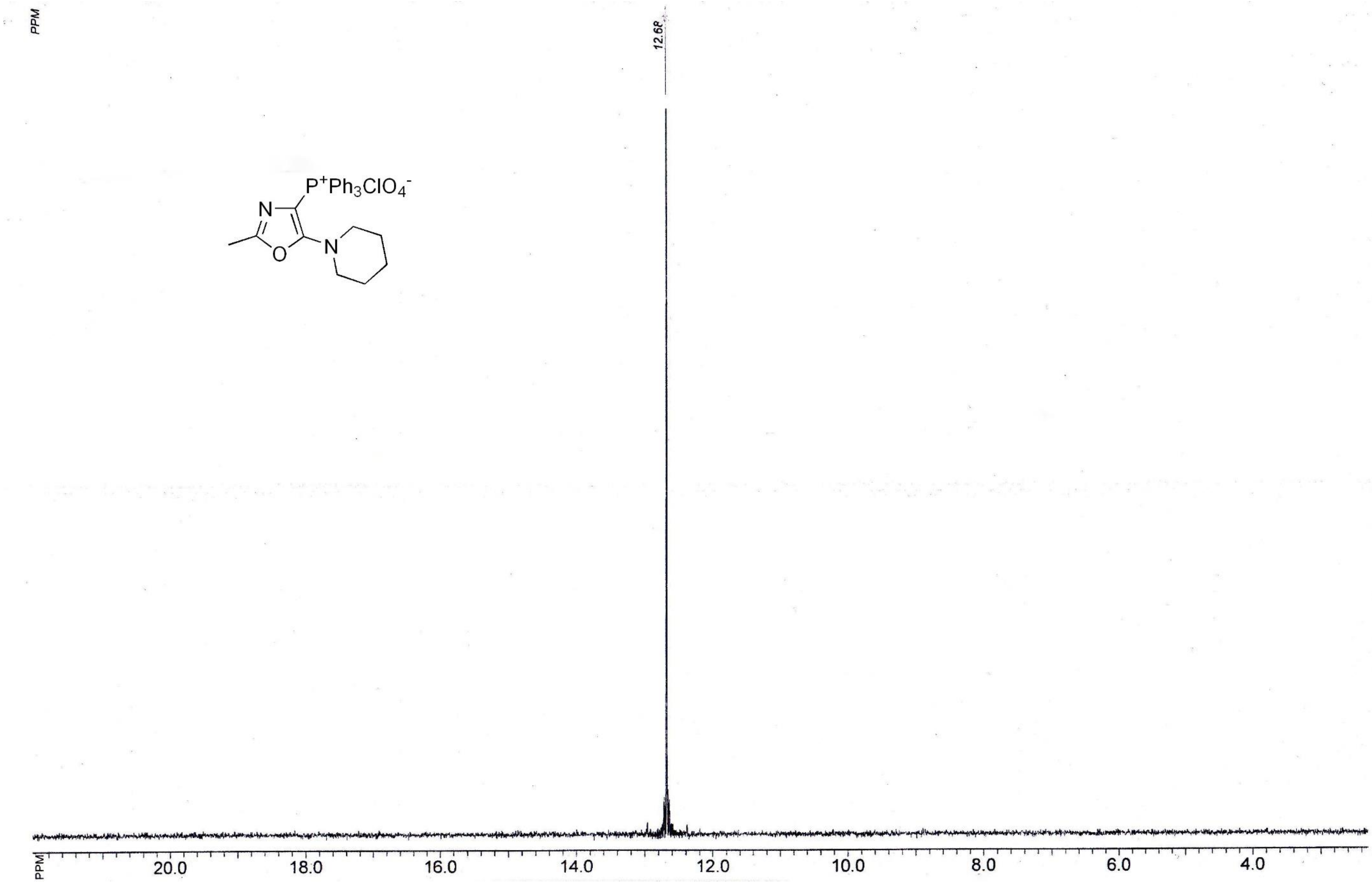

Figure S27. ^{31}P NMR (162 MHz, 294 K, DMSO-d_6) spectrum of compound (**6**).

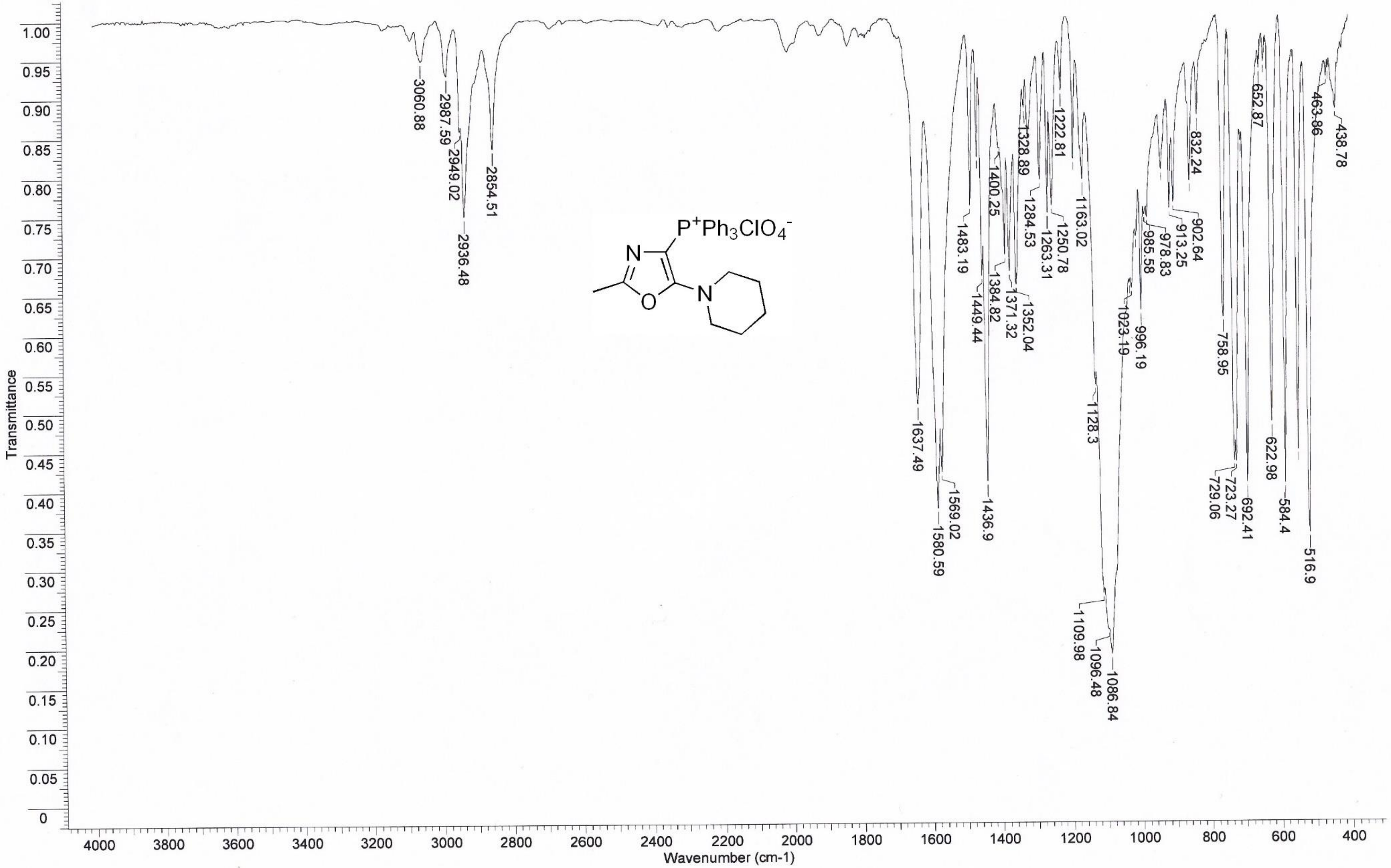

Figure S28. IR spectrum of compound (**6**).

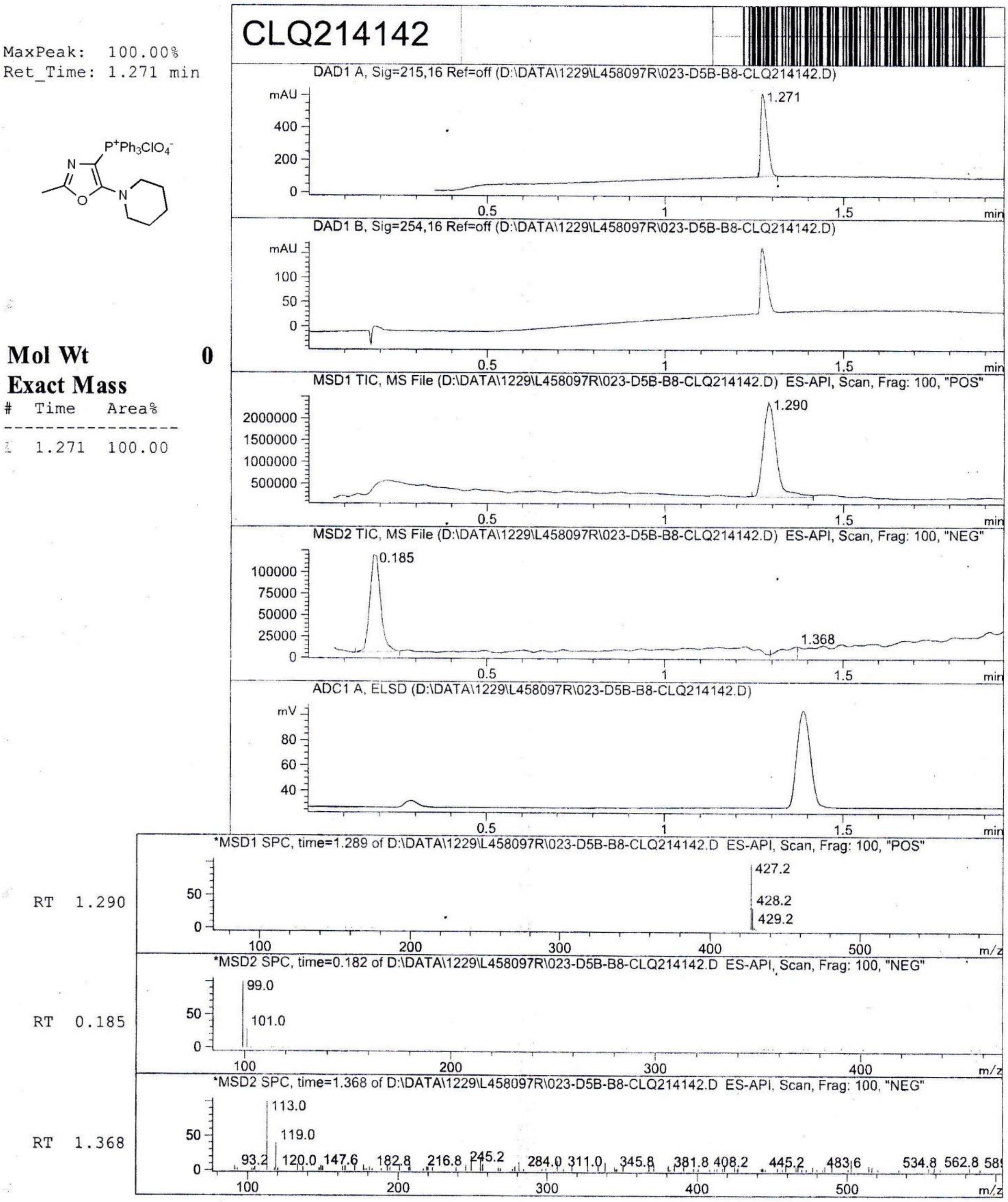

Figure S29. LCMS spectrum of compound **(6).**

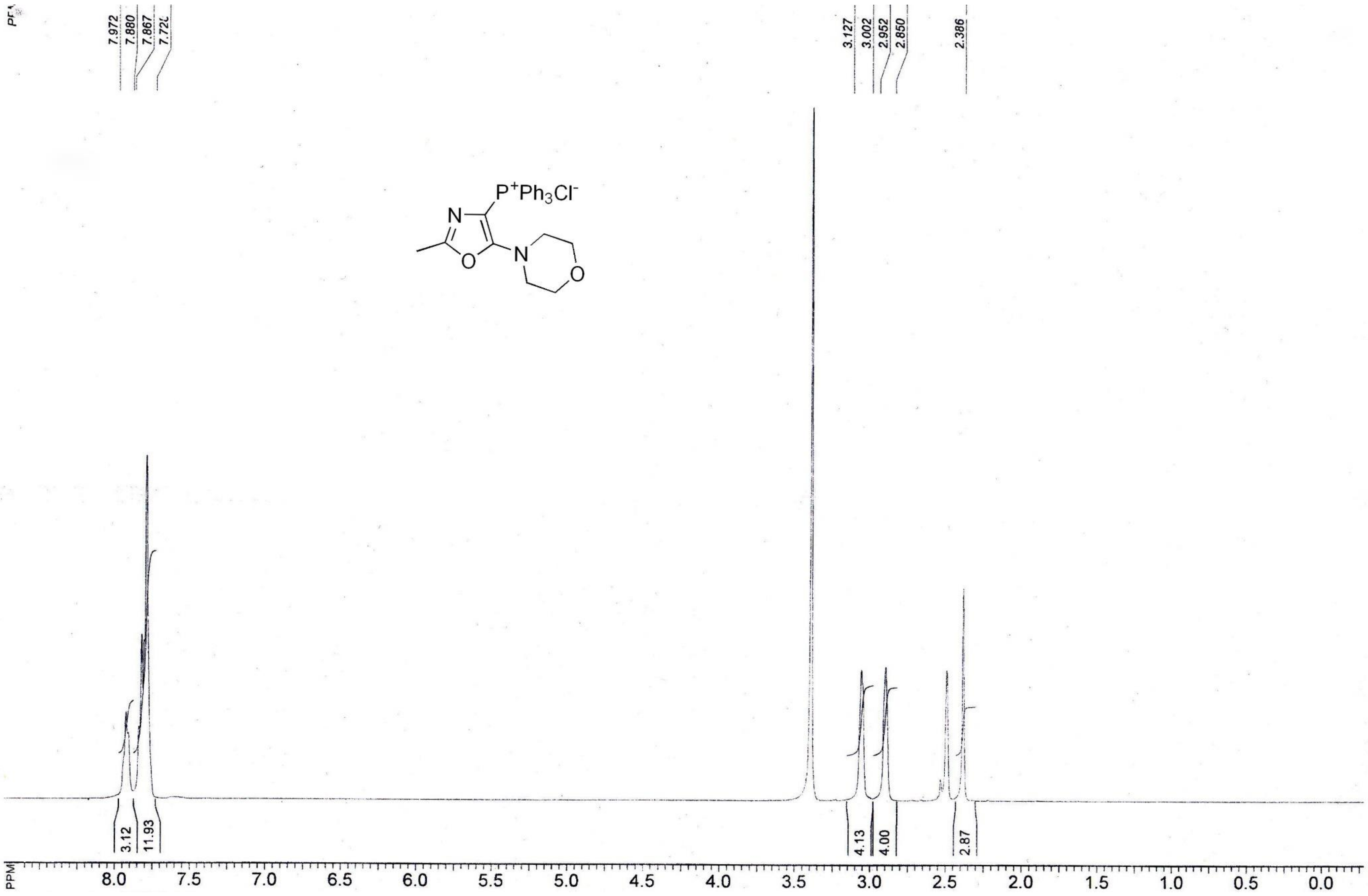

Figure S30. ^{1}H NMR (400 MHz, 294 K, DMSO-d_6) spectrum of compound (**7**).

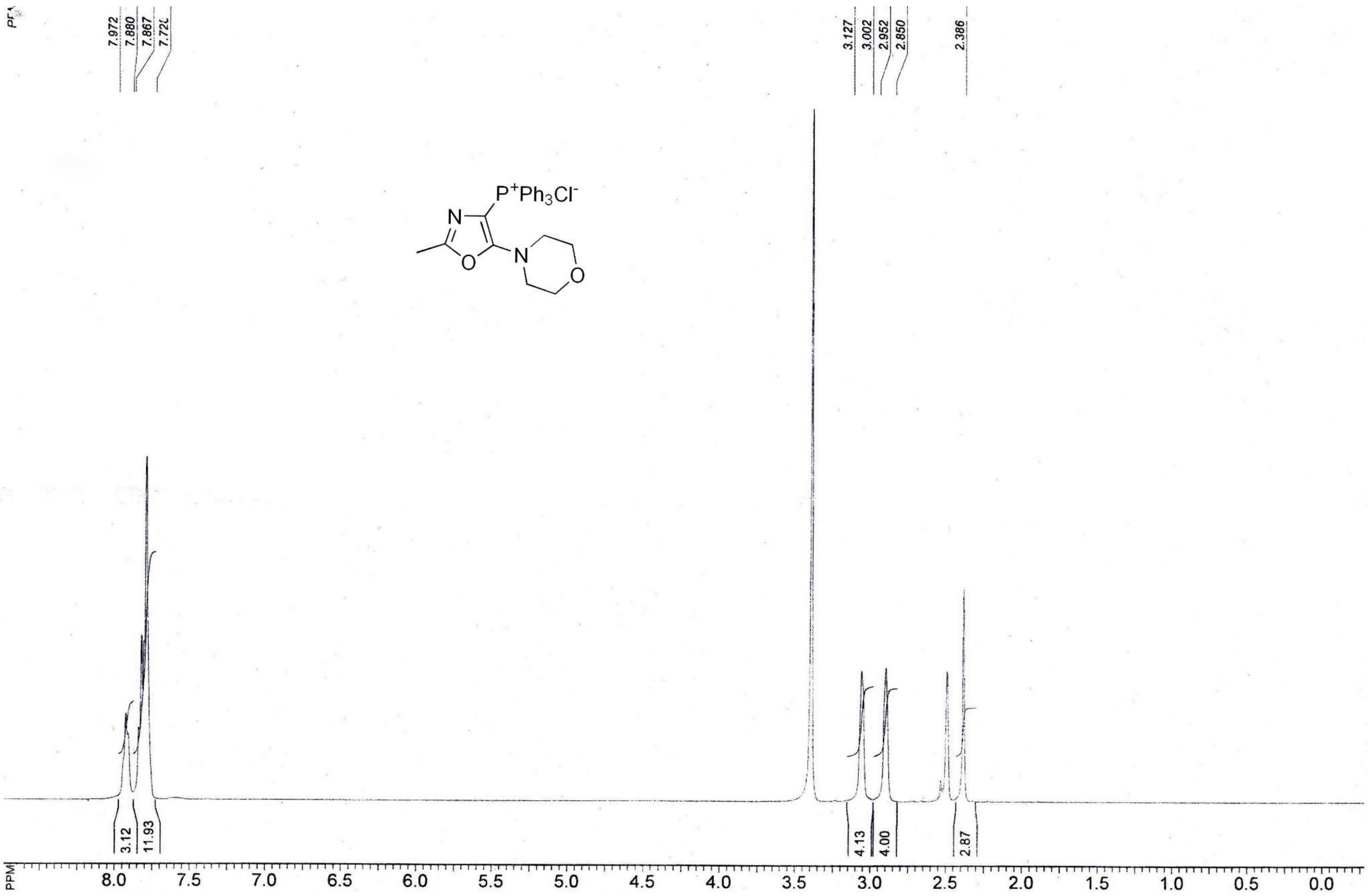

Figure S31. ^{13}C NMR (151 MHz, 298 K, DMSO-d_6) spectrum of compound (**7**).

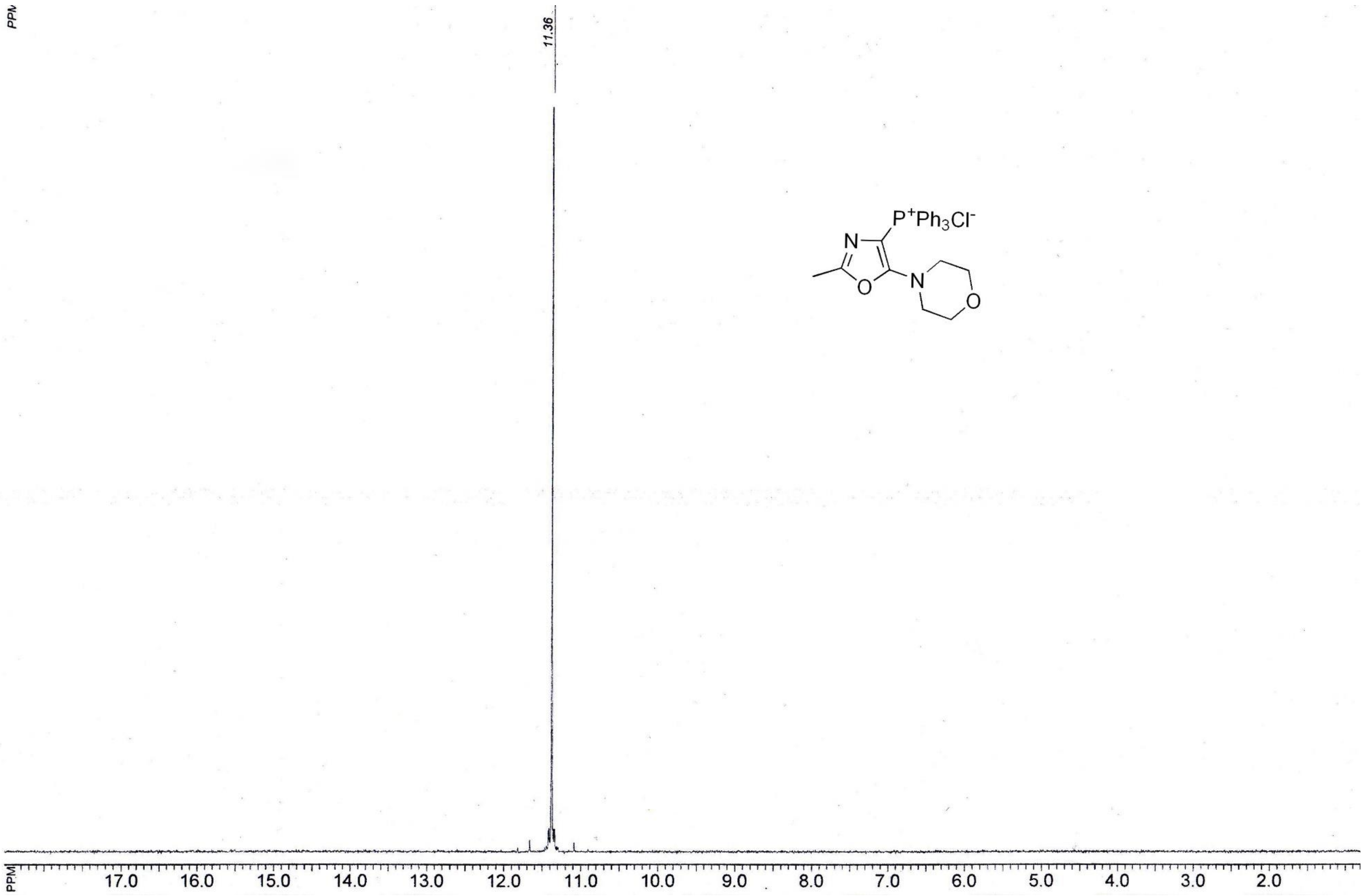

Figure S32. ^{31}P NMR (162 MHz, 294 K, DMSO-d_6) spectrum of compound (**7**).

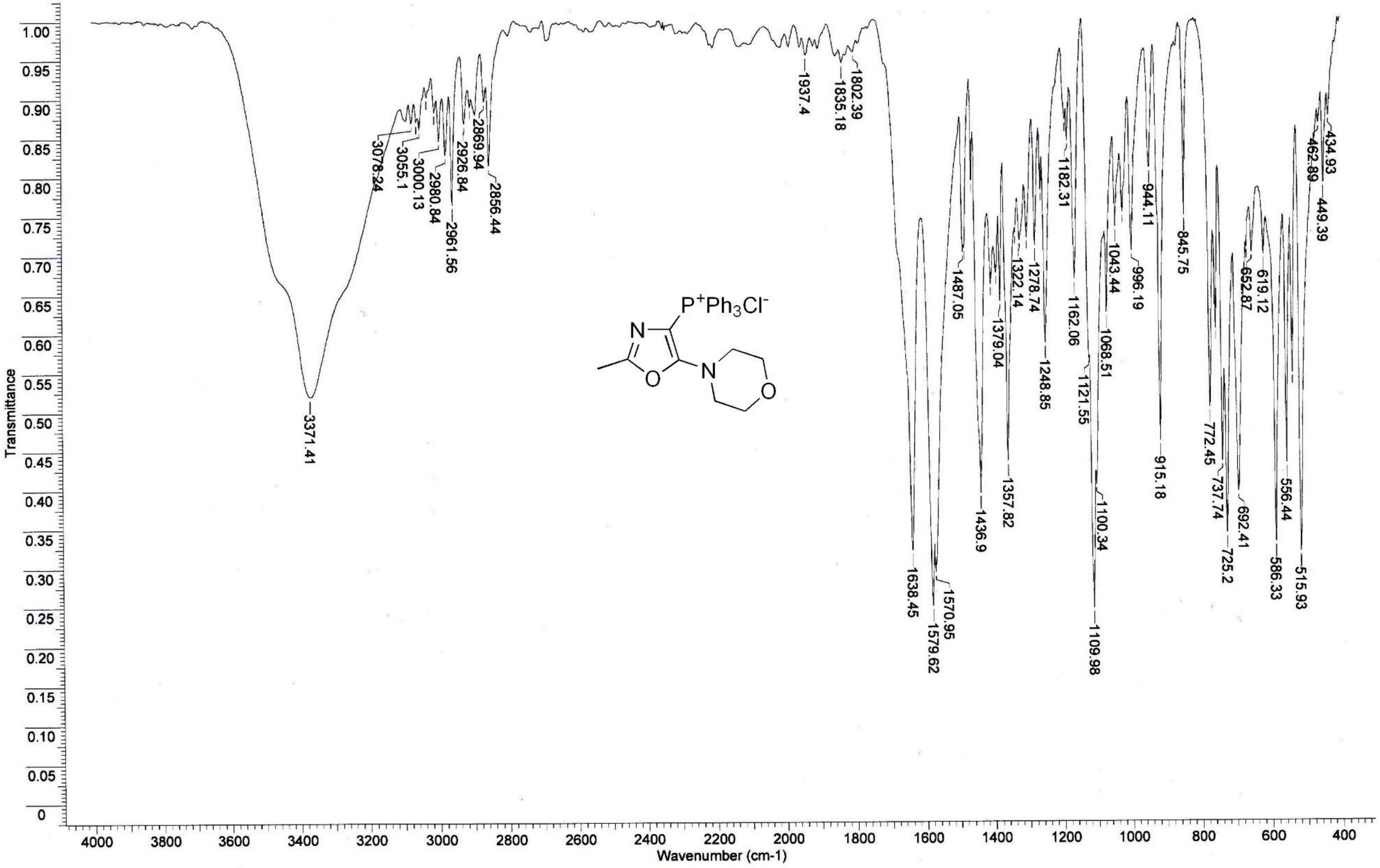

Figure S33. IR spectrum of compound (**7**).

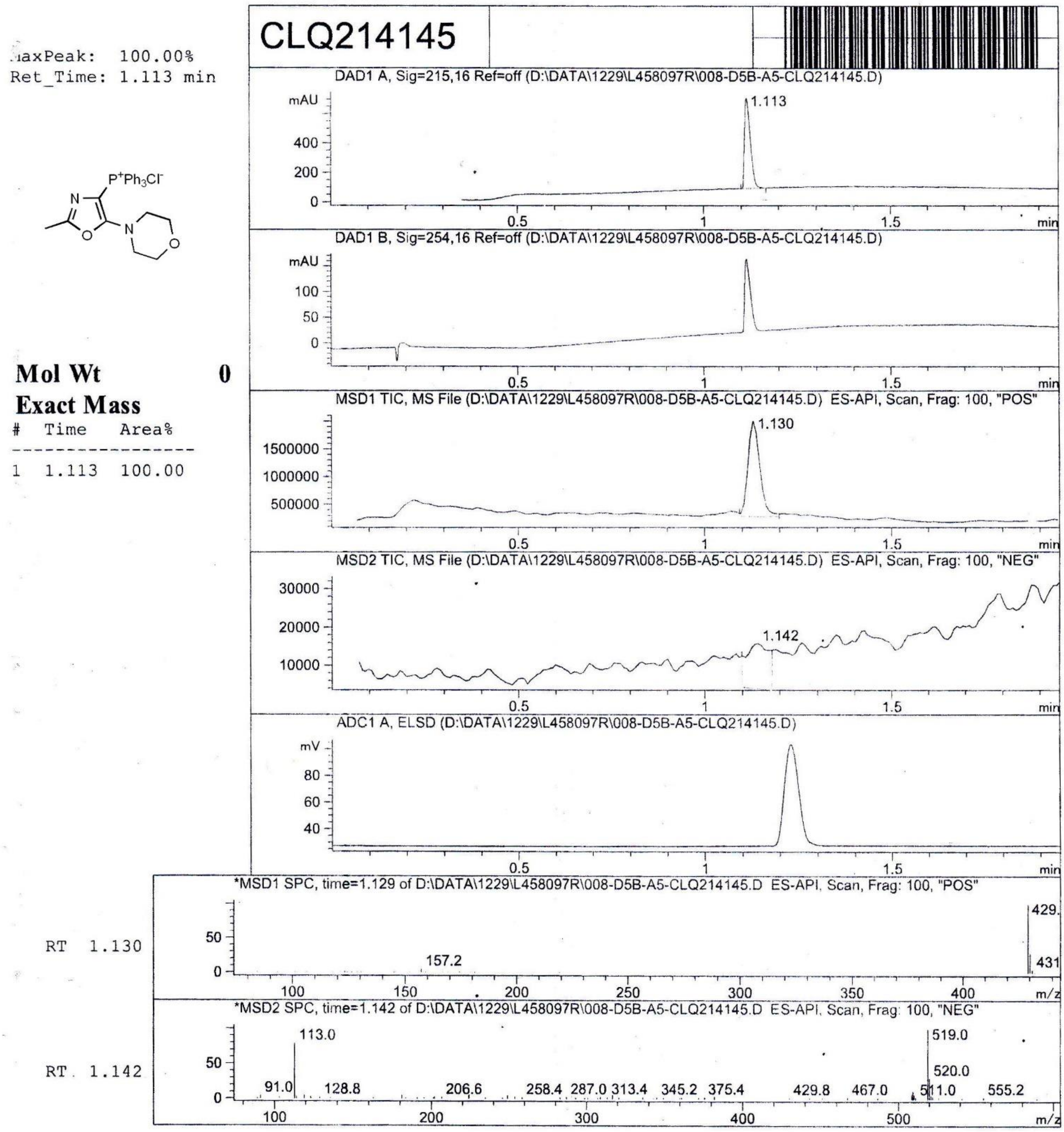

Figure S34. LCMS spectrum of compound (**7**).

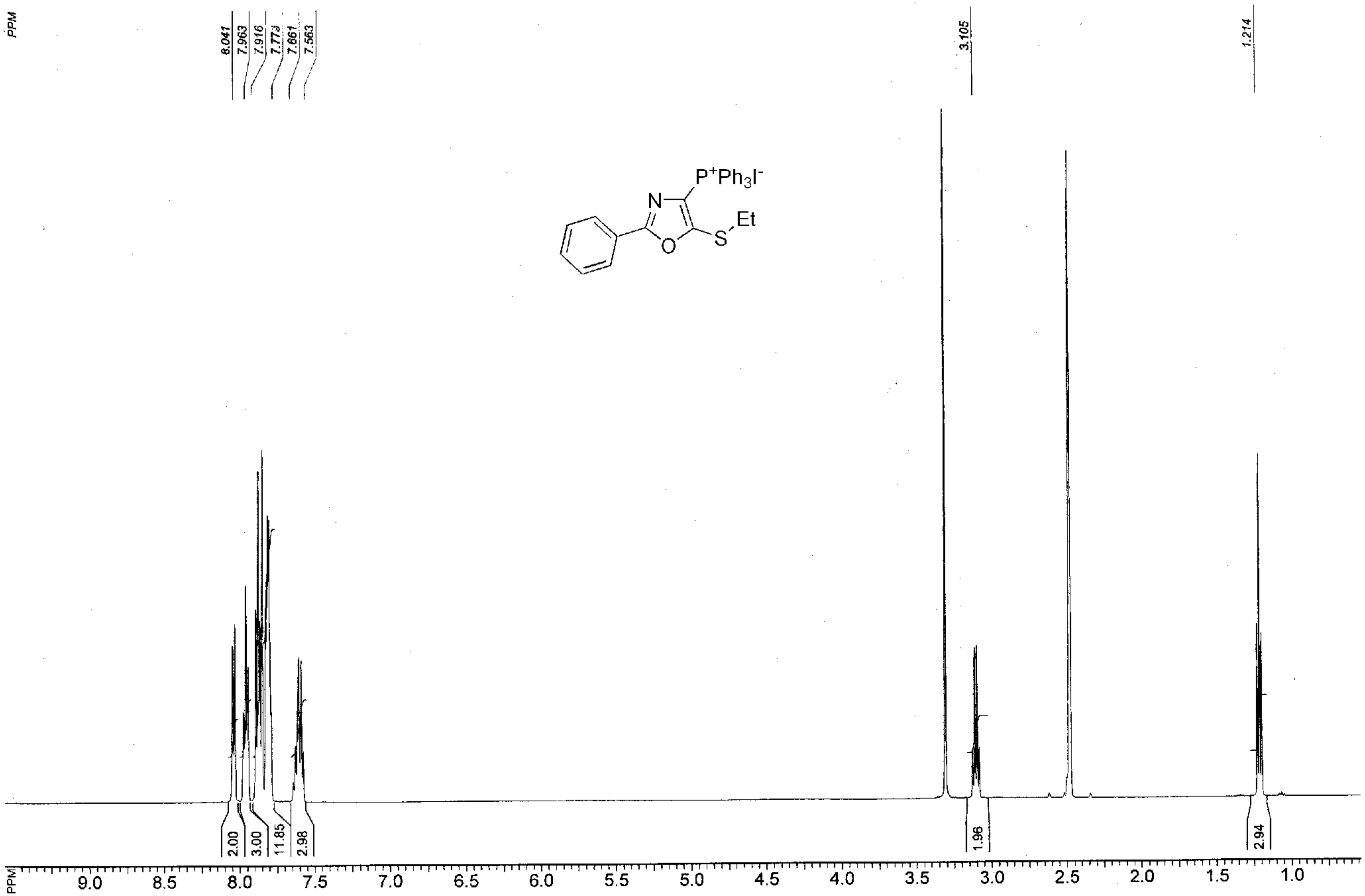

Figure S35. ^{1}H NMR (400 MHz, 298 K, DMSO-d_6) spectrum of compound (**8**).

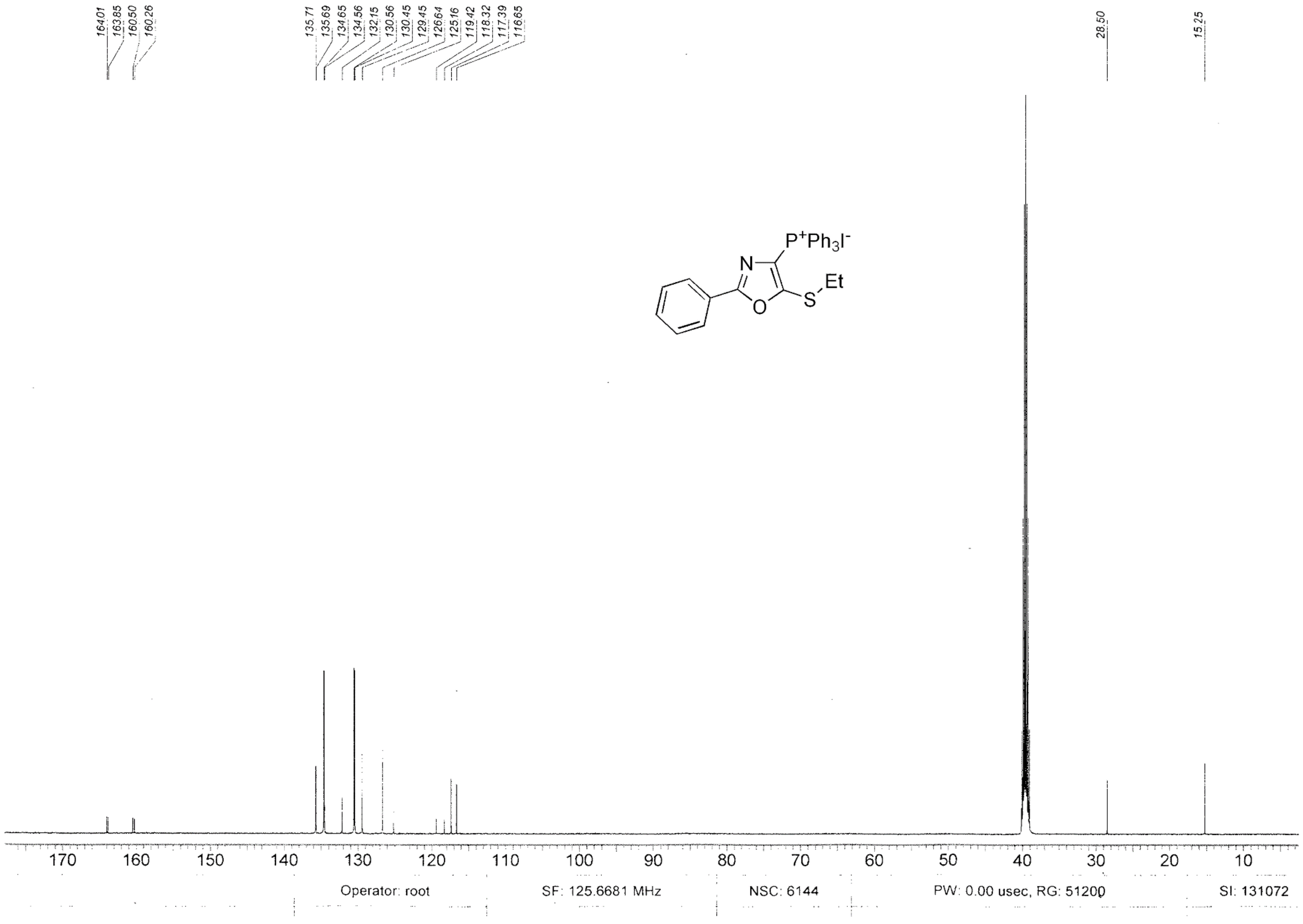

Figure S36. ^{13}C NMR (127 MHz, 298 K, DMSO-d_6) spectrum of compound (**8**).

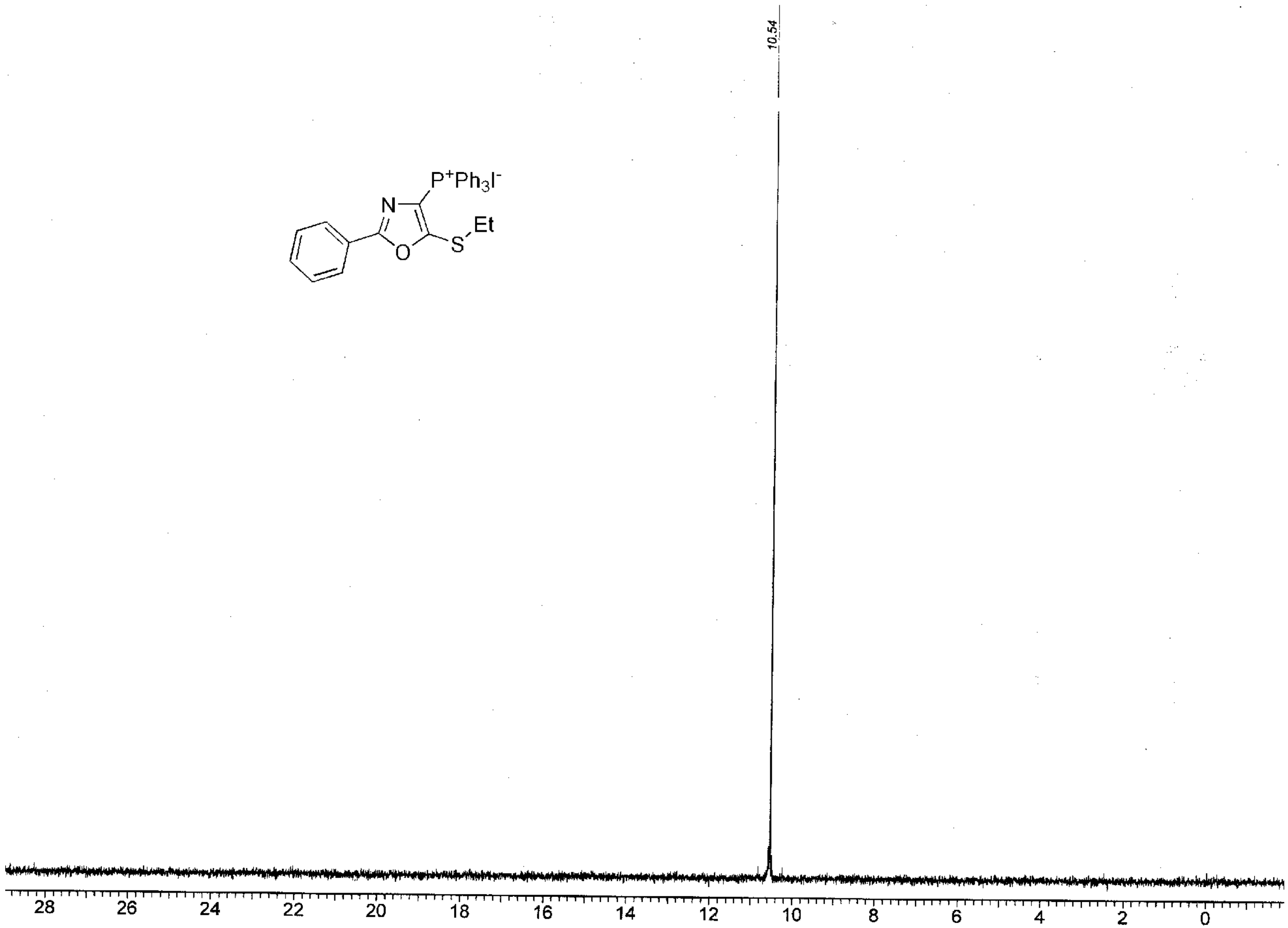

Figure S37. ^{31}P NMR (162 MHz, 294 K, DMSO-d_6) spectrum of compound (**8**).

MaxPeak: 100.00%
Ret_Time: 1.400 min

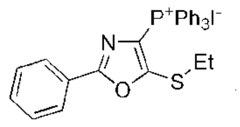

Mol Wt 0
Exact Mass

#	Time	Area%
1	1.400	100.00

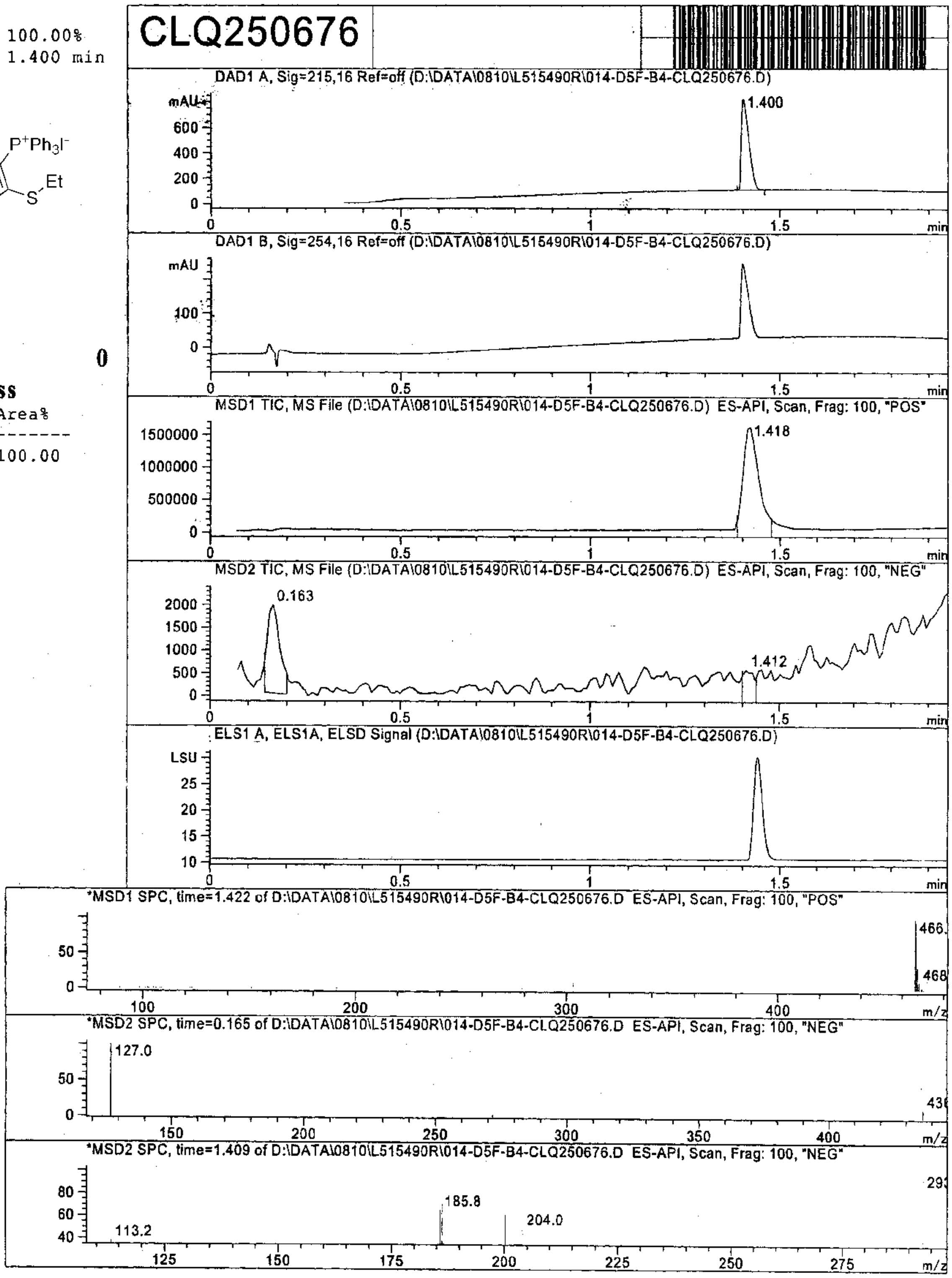

Figure S38. LCMS spectrum of compound (**8**).

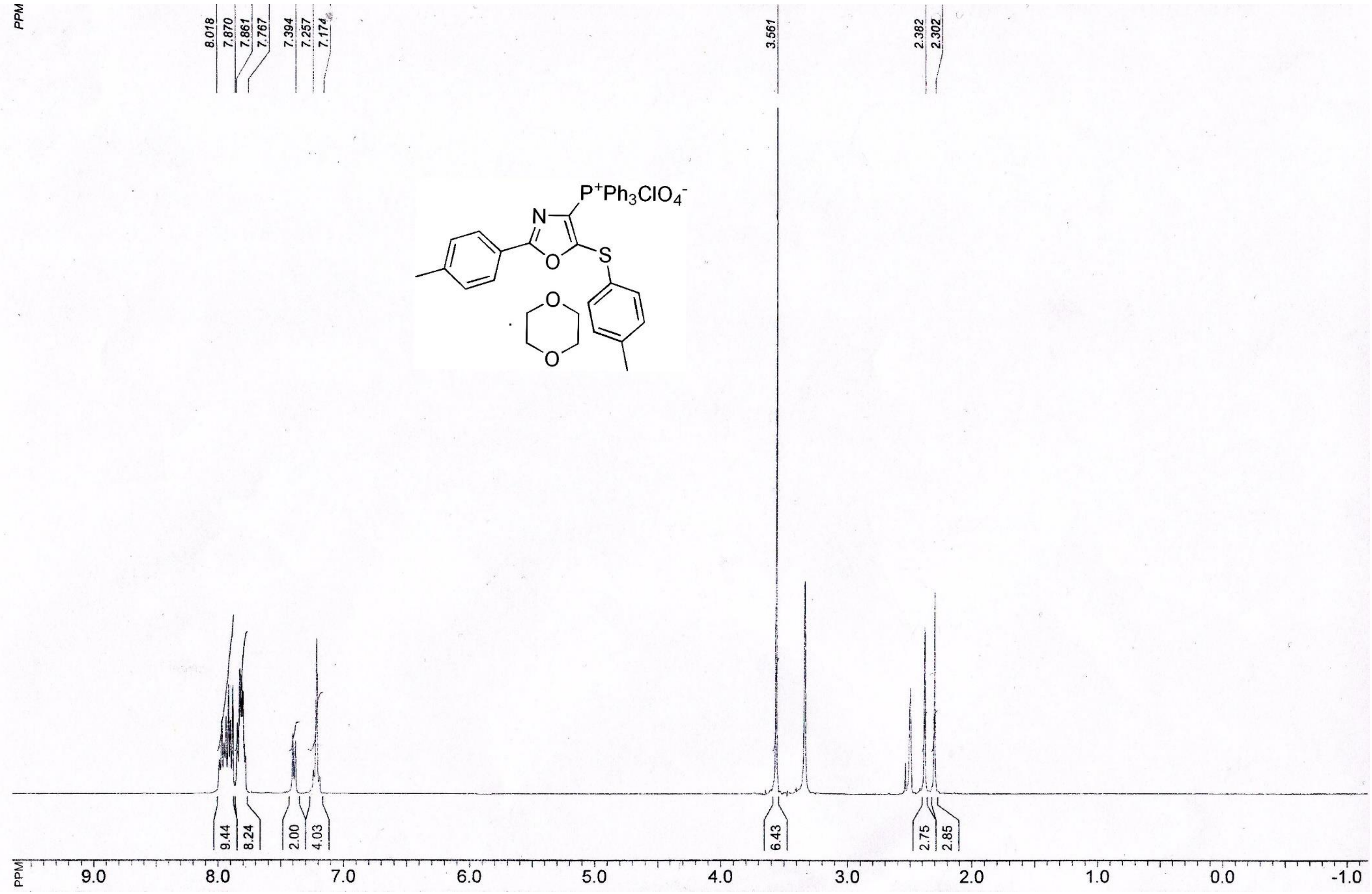

Figure S39. ^{1}H NMR (400 MHz, 298 K, DMSO-d_6) spectrum of compound (**9**).

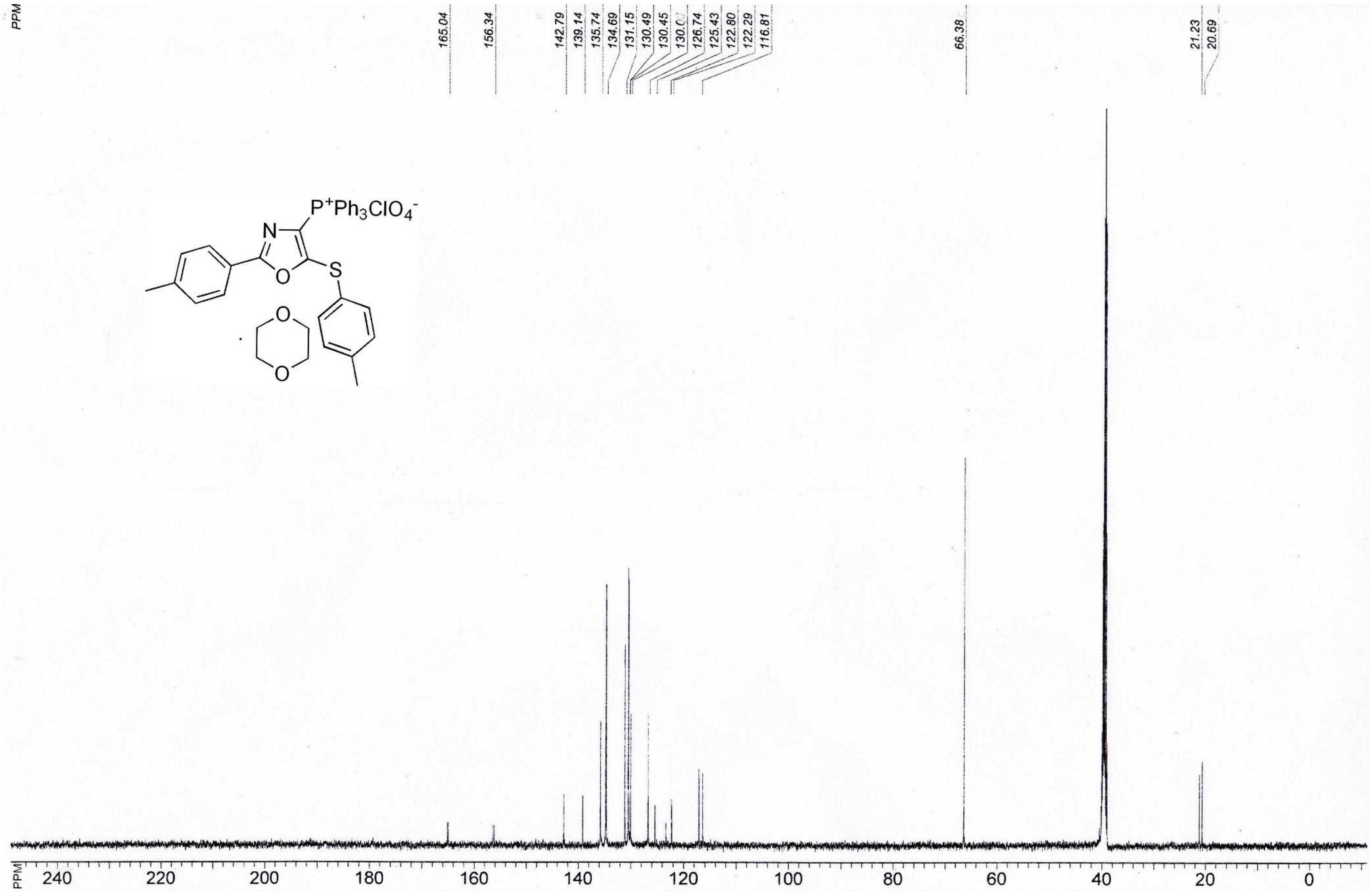

Figure S40. ^{13}C NMR (126 MHz, 683 K, DMSO-d_6) spectrum of compound (**9**).

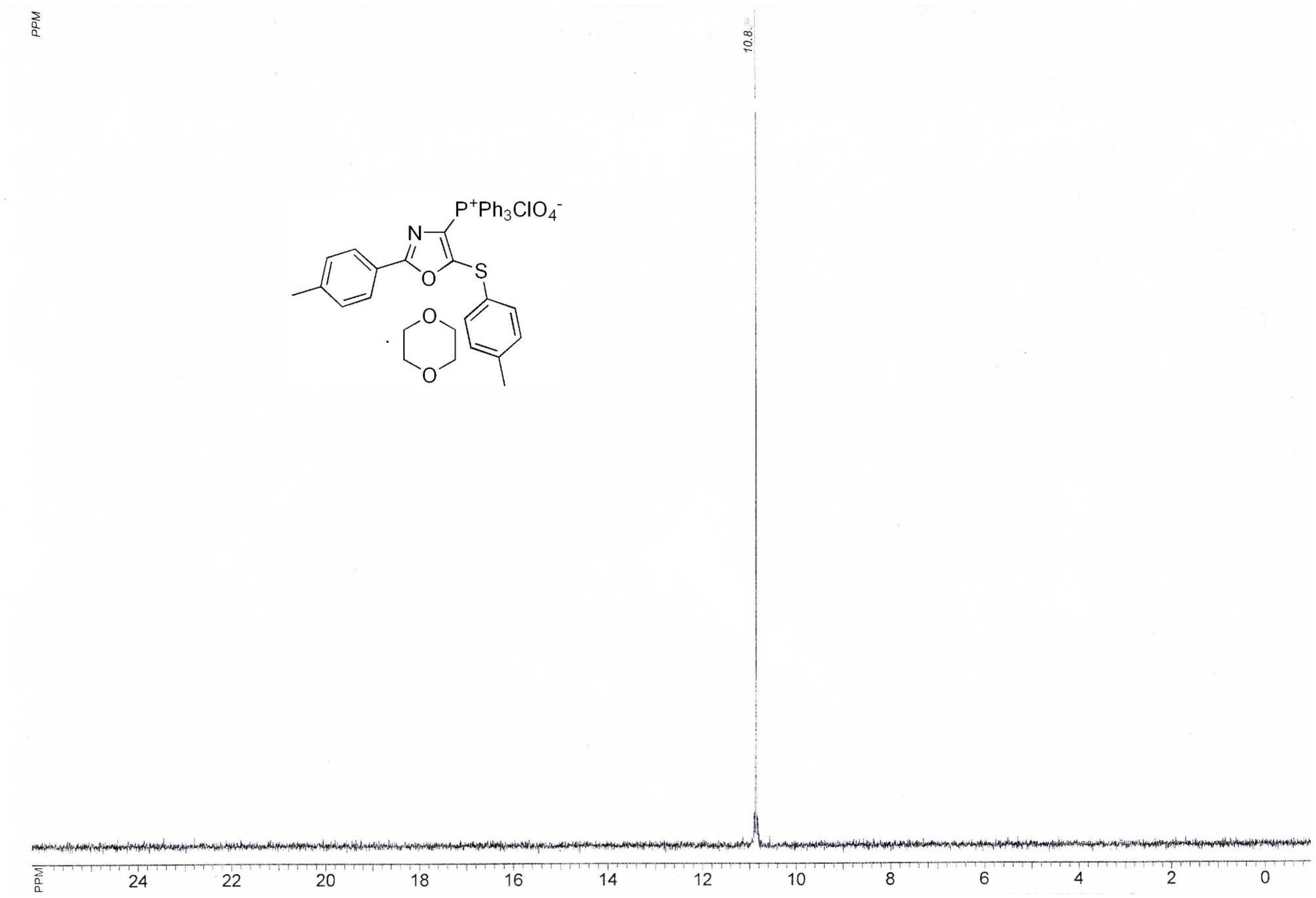

Figure S41. ^{31}P NMR (162 MHz, 294 K, DMSO-d_6) spectrum of compound (**9**).

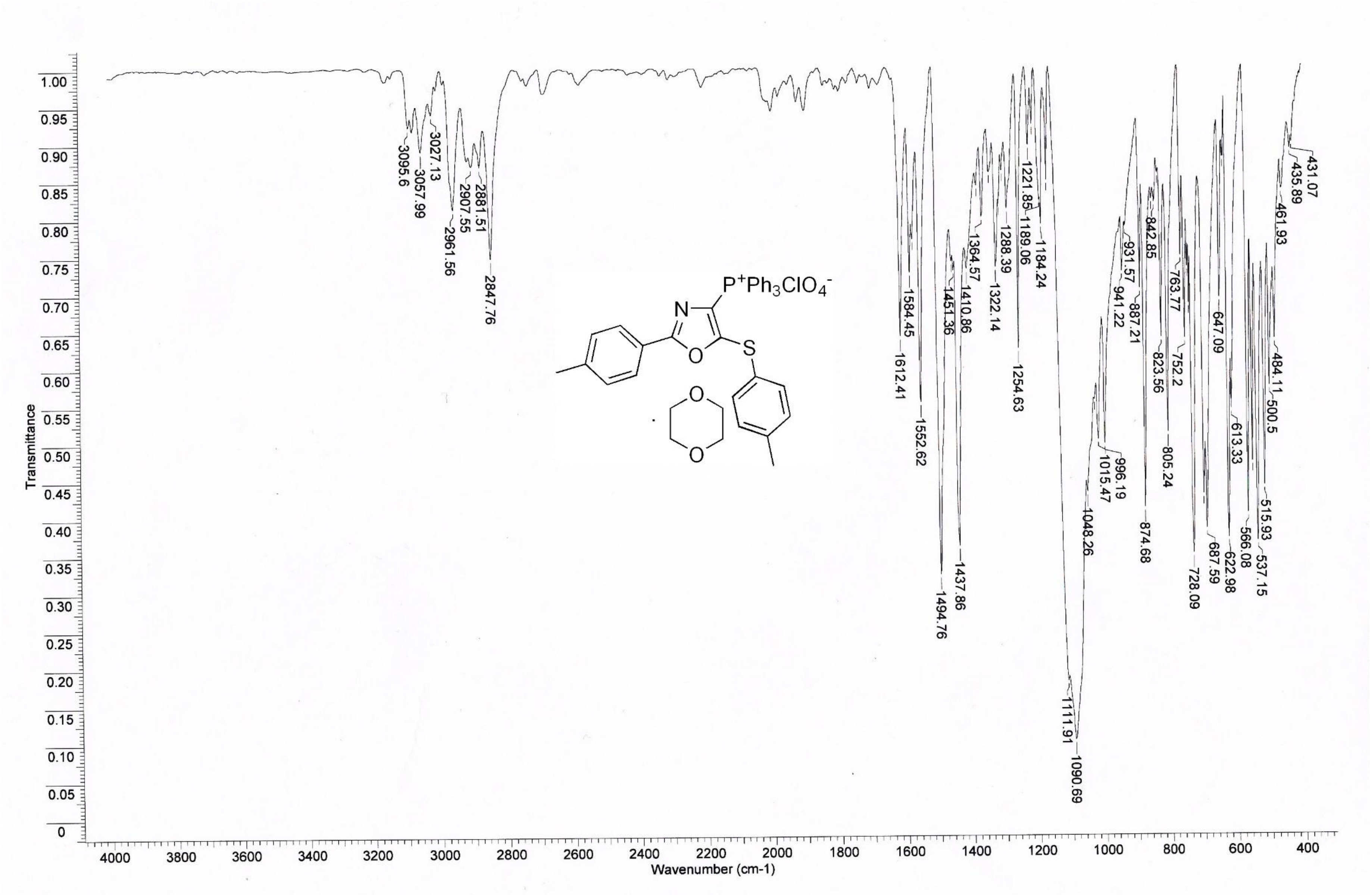

Figure S42. IR spectrum of compound (**9**).

MaxPeak: 96.36%
Ret_Time: 1.551 min

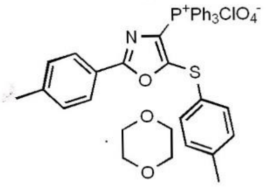

Mol Wt 0
Exact Mass

#	Time	Area%
1	1.297	1.68
2	1.389	1.95
3	1.551	96.36

RT 1.309

RT 1.400

RT 1.569

RT 0.185

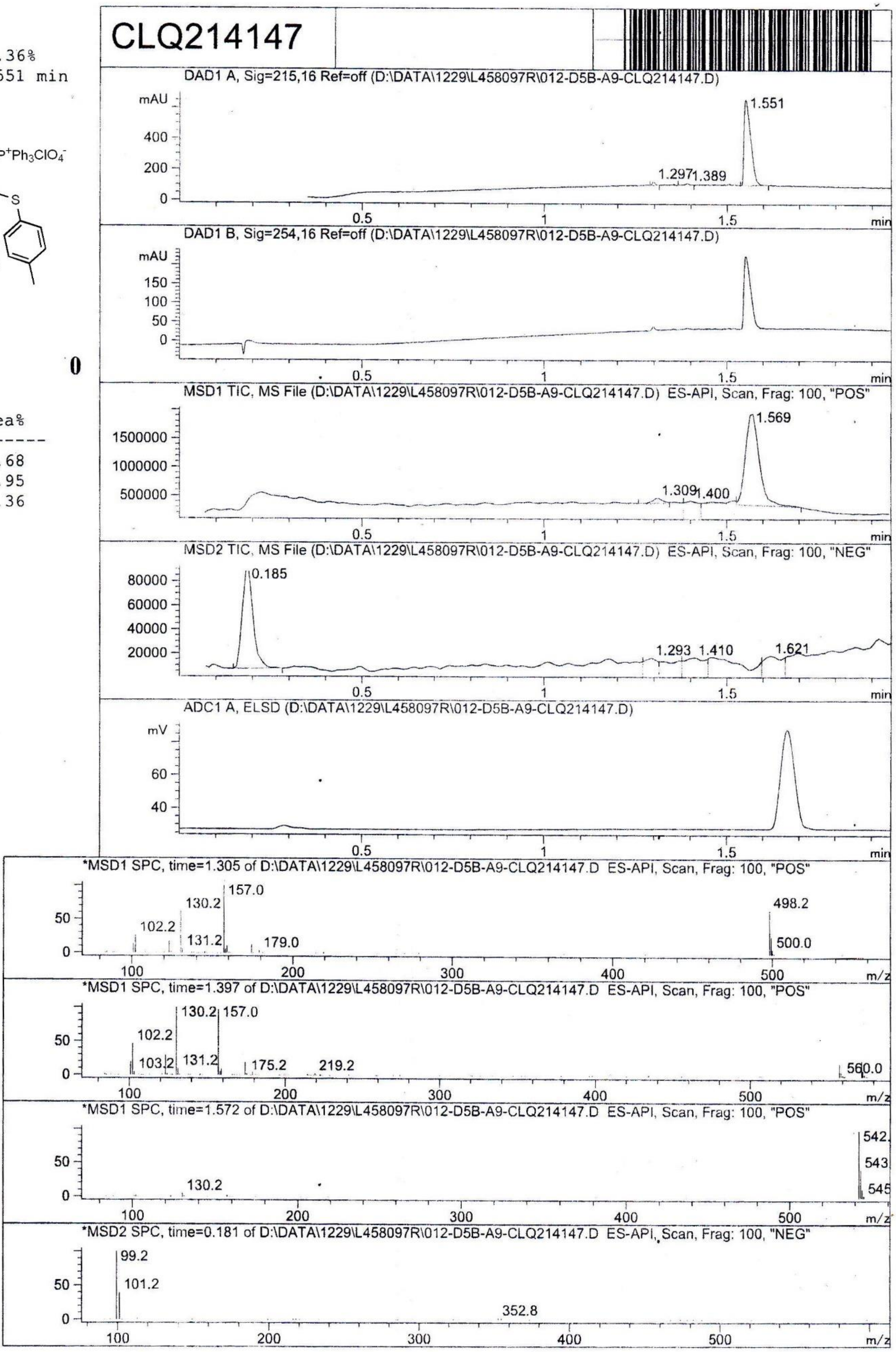

Figure S43. LCMS spectrum of compound (**9**).

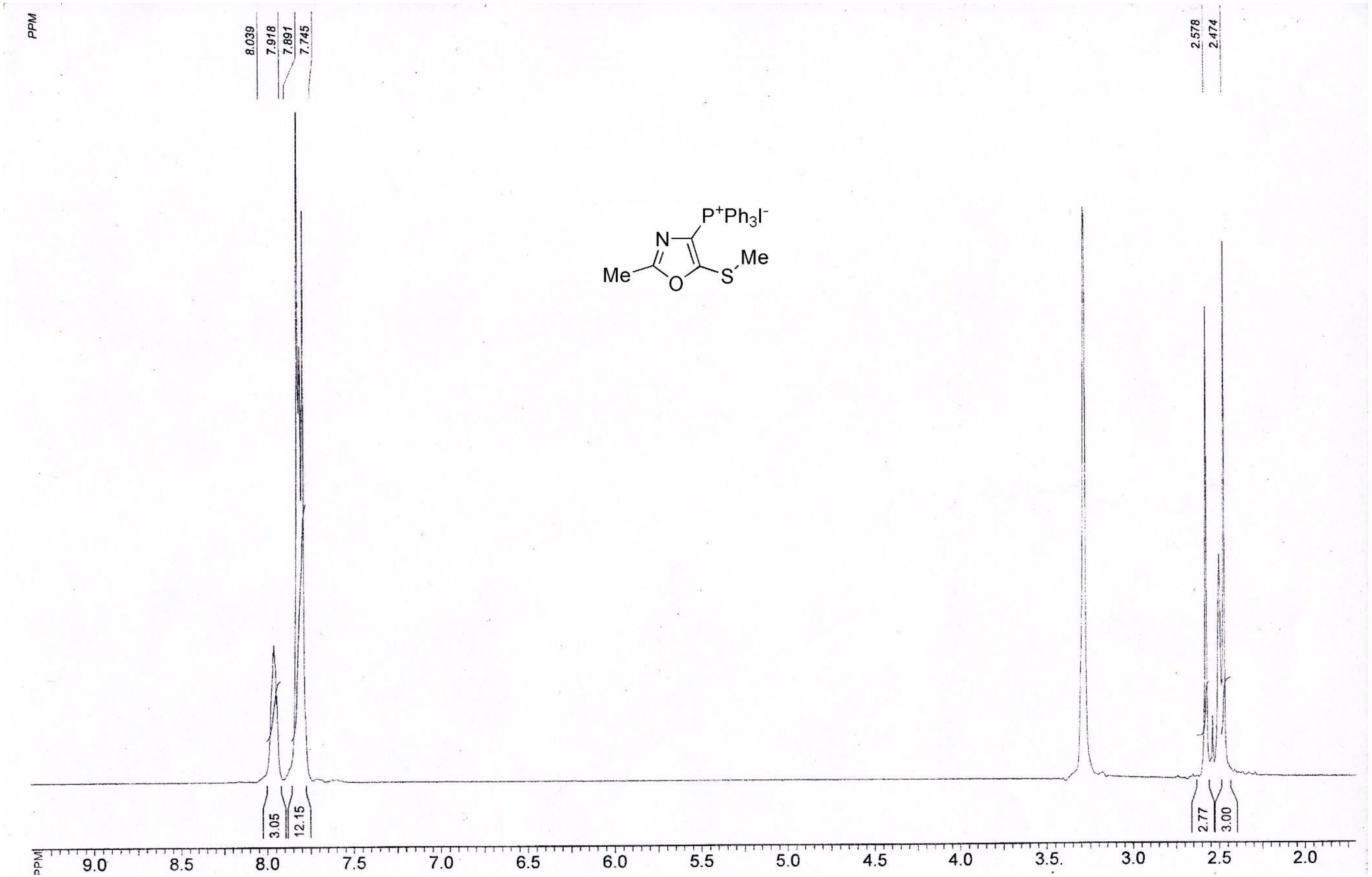

Figure S44. ^{1}H NMR (400 MHz, 298 K, DMSO-d_6) spectrum of compound (**10**).

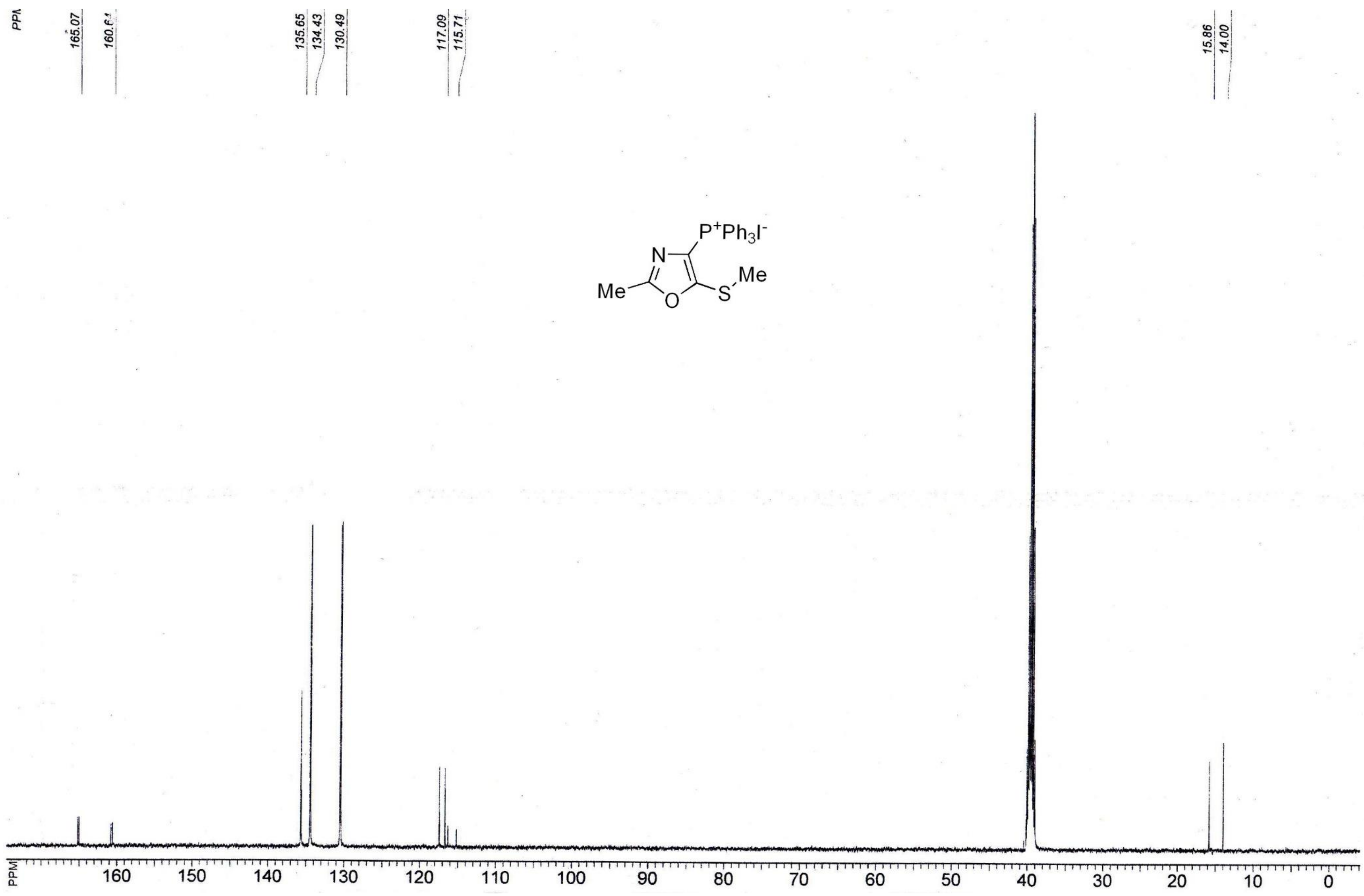

Figure S45. ^{13}C NMR (126 MHz, 683 K, DMSO-d_6) spectrum of compound (**10**).

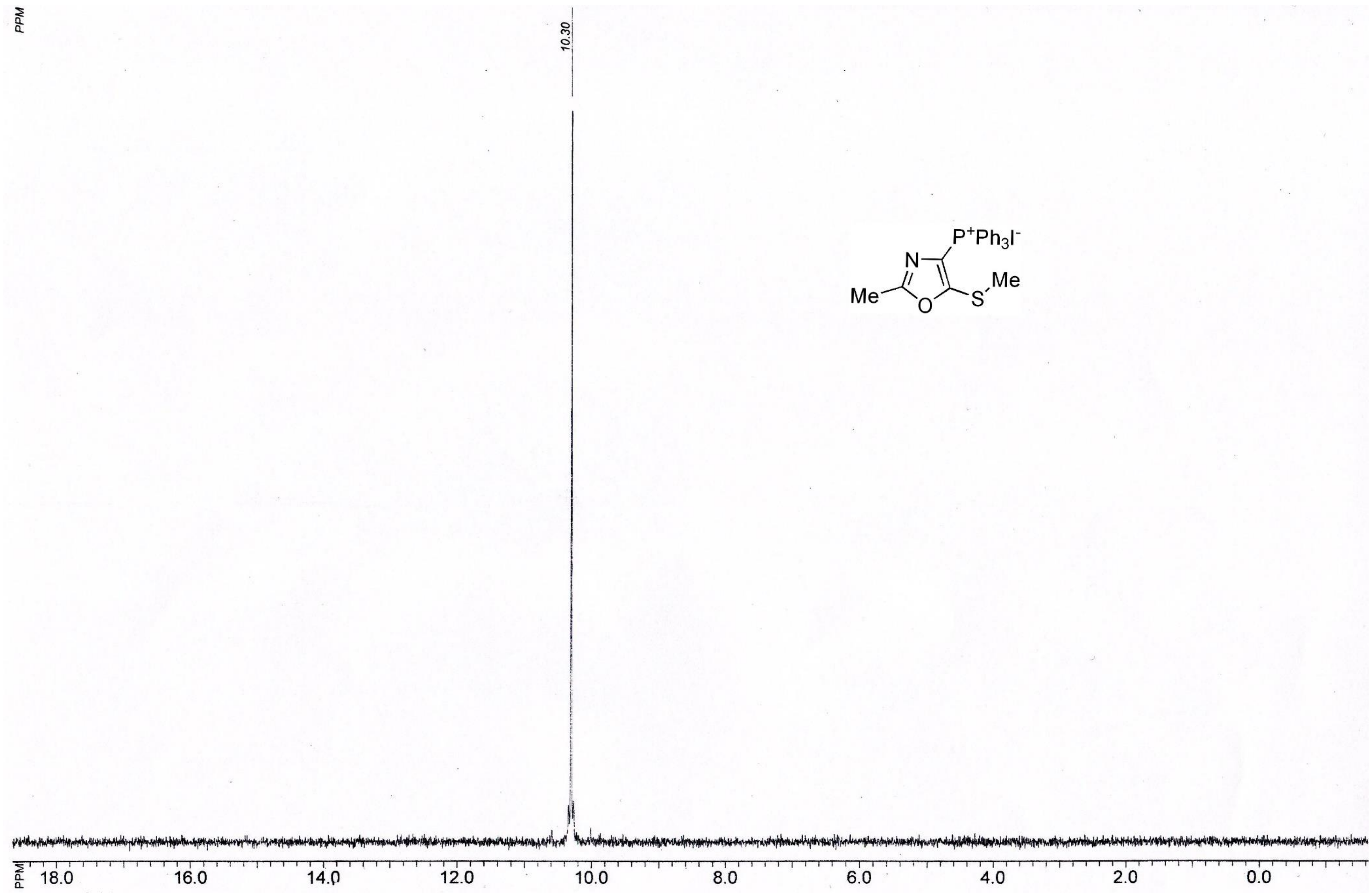

Figure S46. ^{31}P NMR (162 MHz, 294 K, DMSO-d_6) spectrum of compound (**10**).

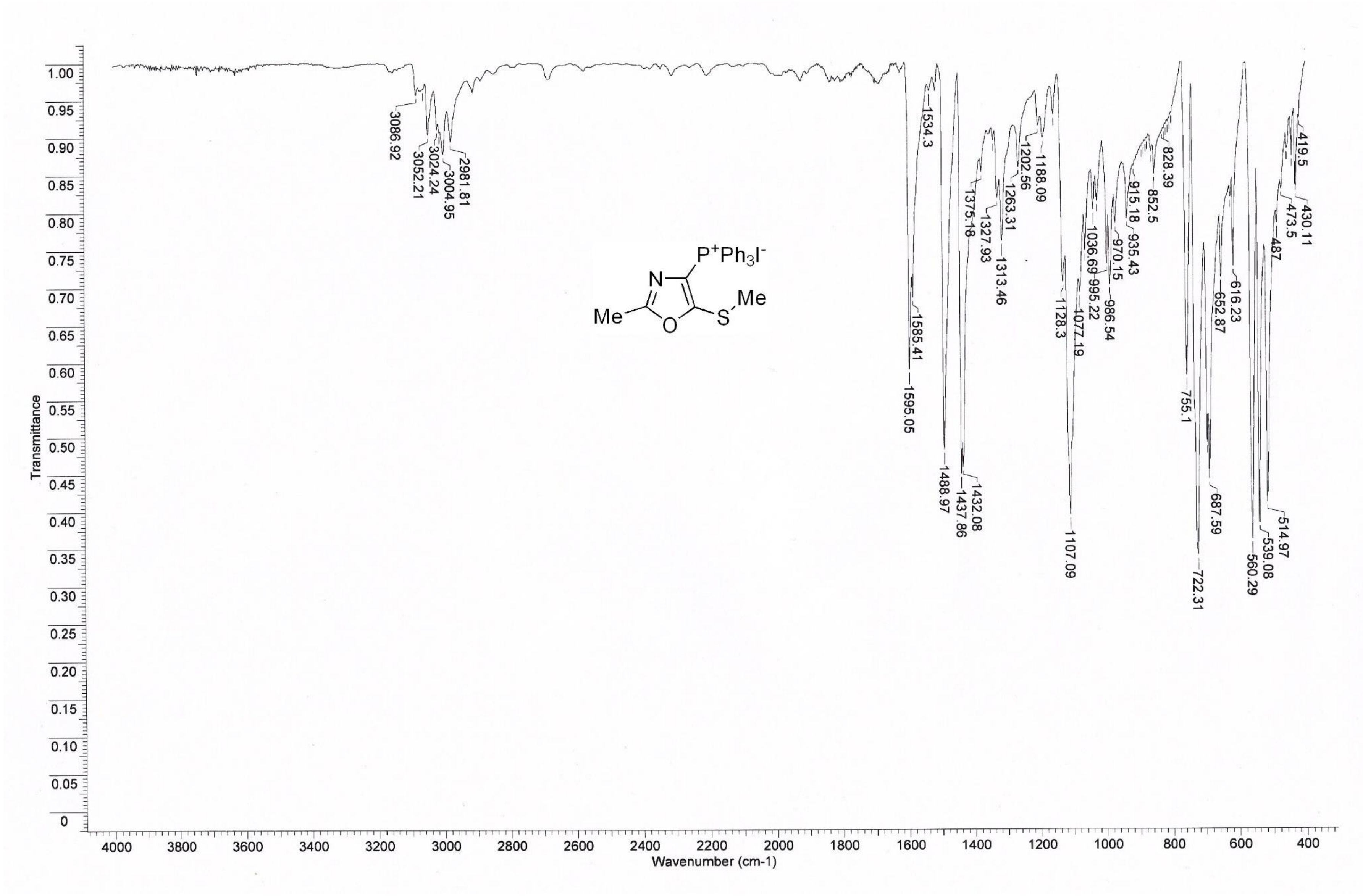

Figure S47. IR spectrum of compound (**10**).

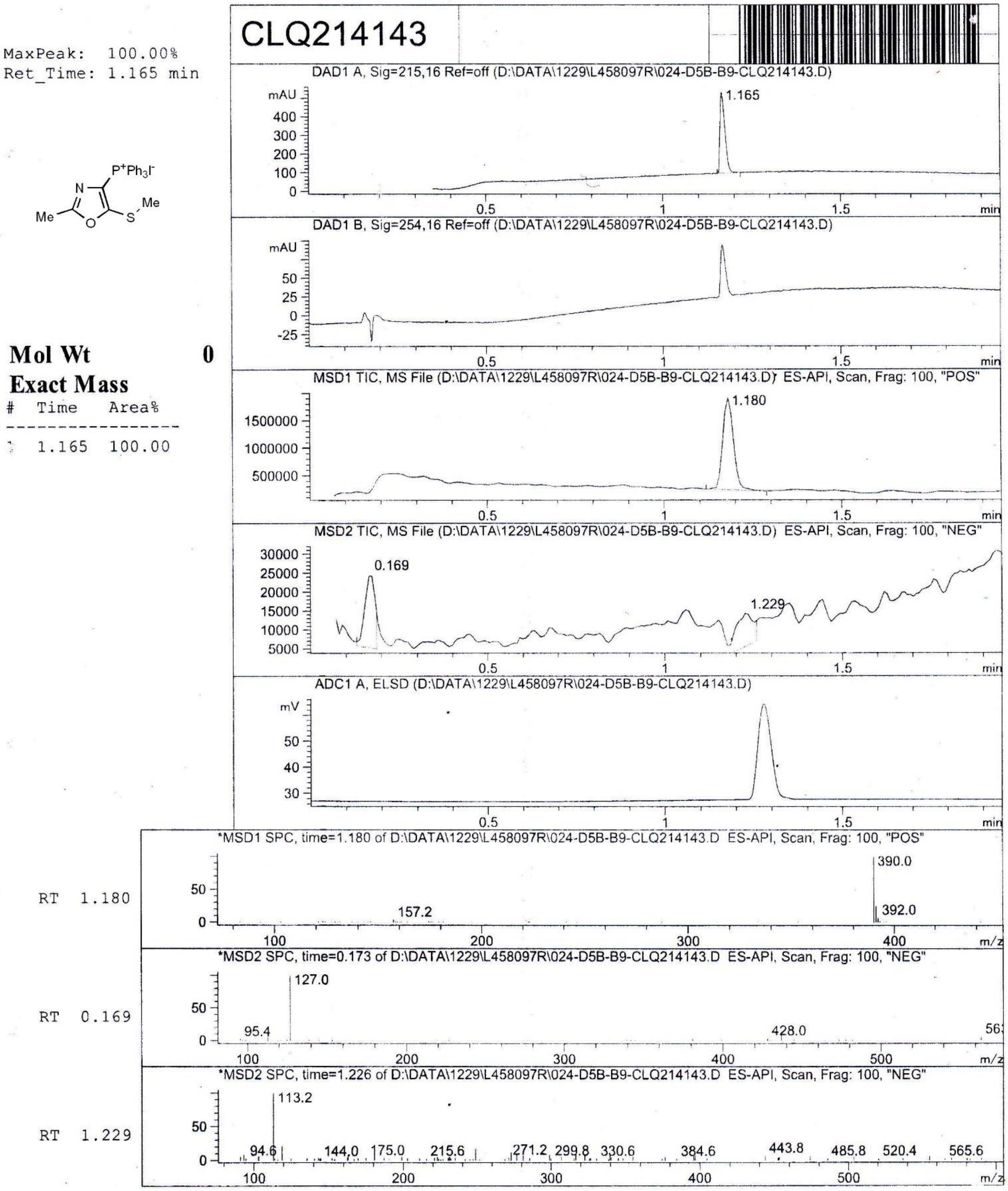

Figure S48. LCMS spectrum of compound (**10**).

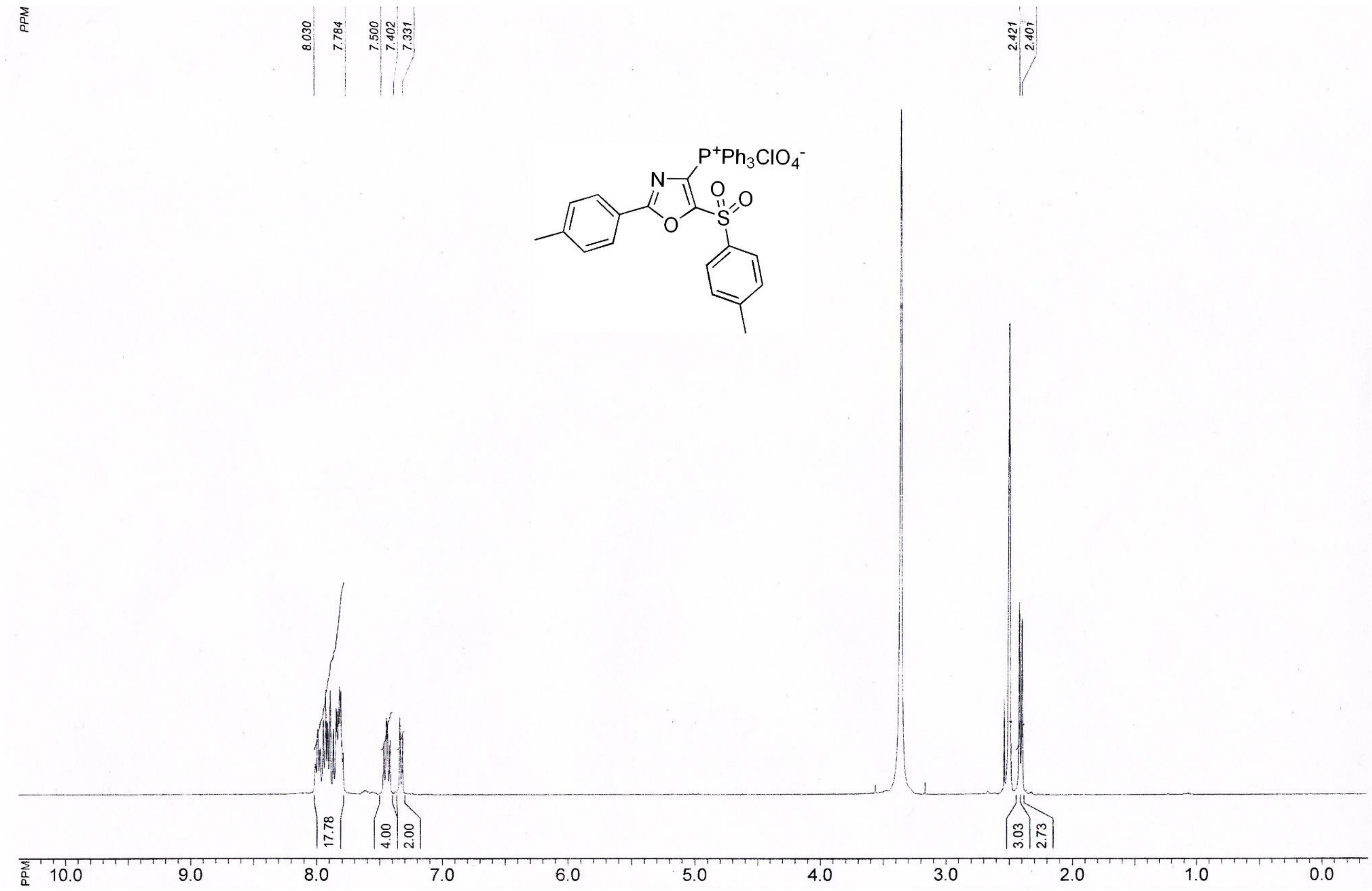

Figure S49. ^{1}H NMR (400 MHz, 298 K, DMSO-d_6) spectrum of compound (**11**).

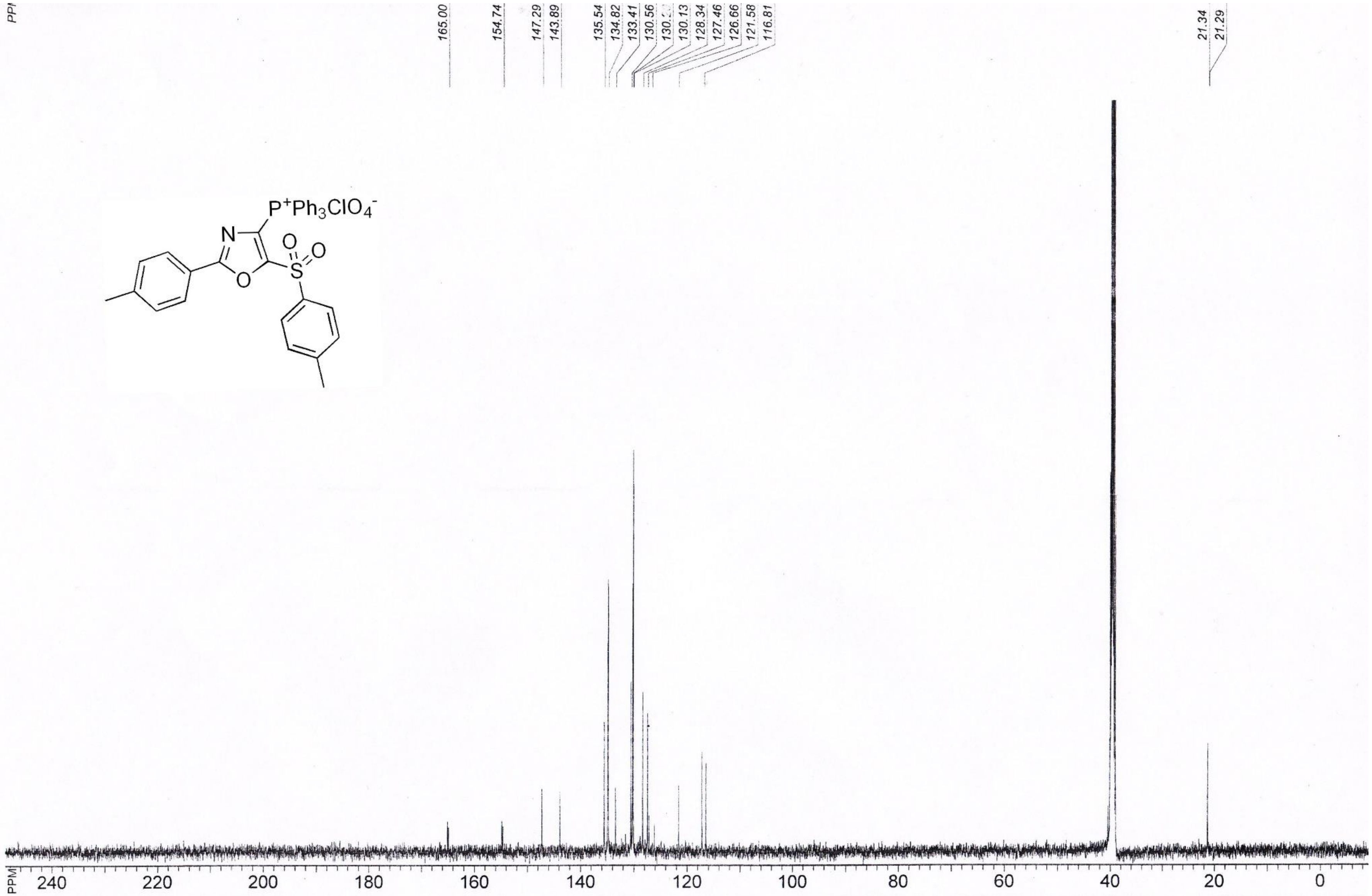

Figure S50. ^{13}C NMR (126 MHz, 683 K, DMSO-d_6) spectrum of compound (**11**).

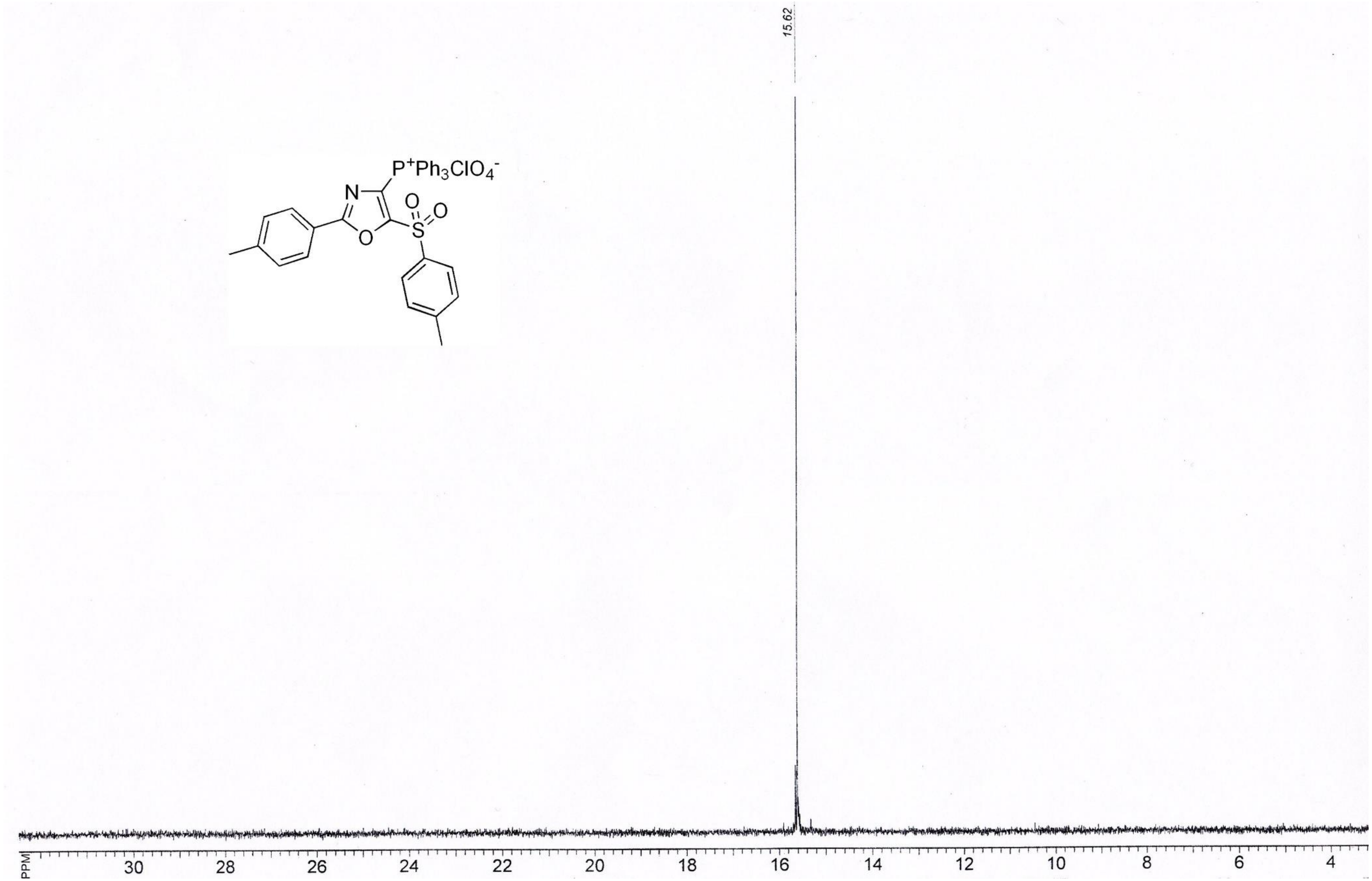

Figure S51. ^{31}P NMR (162 MHz, 294 K, DMSO-*d*$_6$) spectrum of compound (**11**).

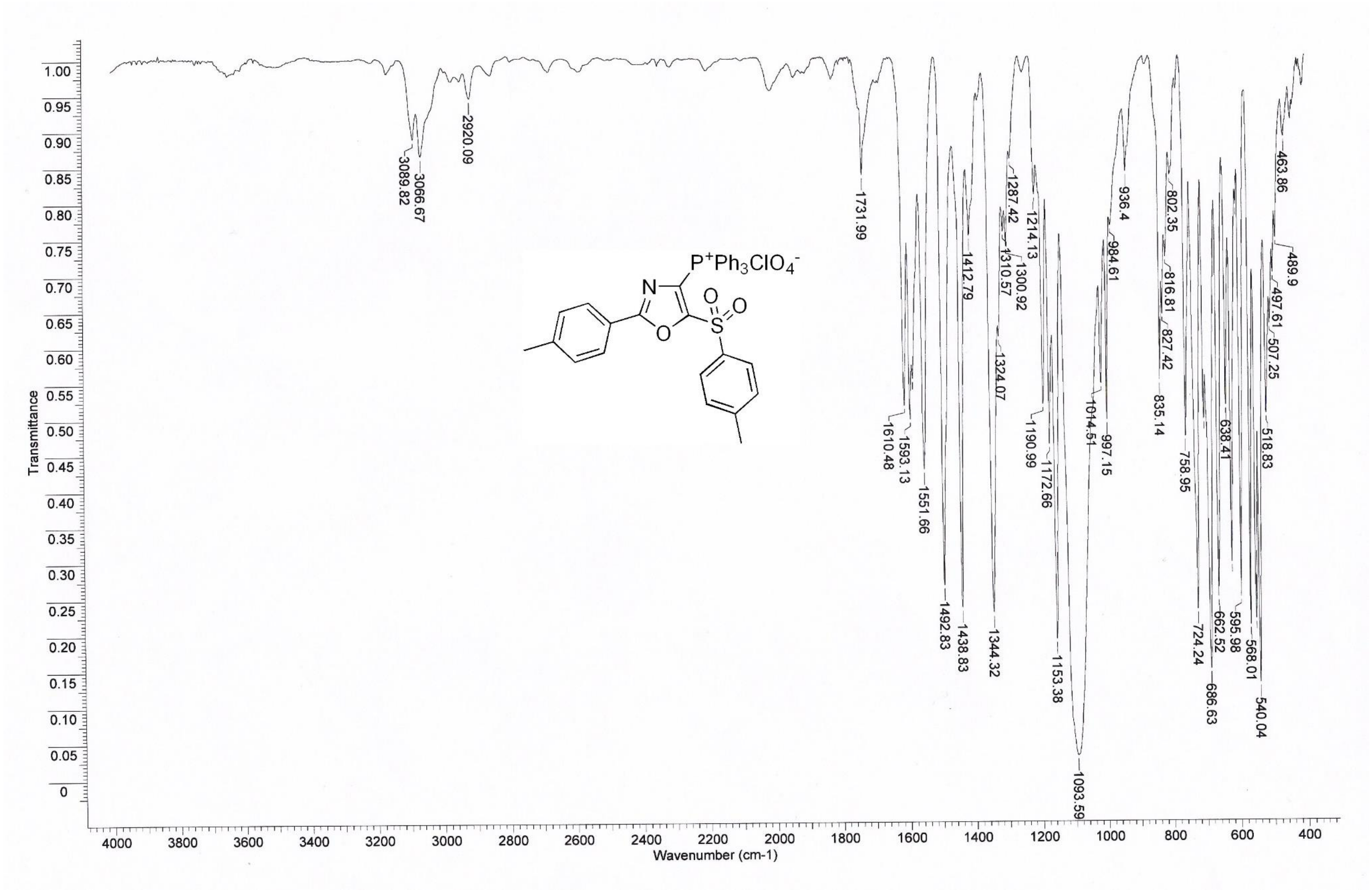

Figure S52. IR spectrum of compound (**11**).

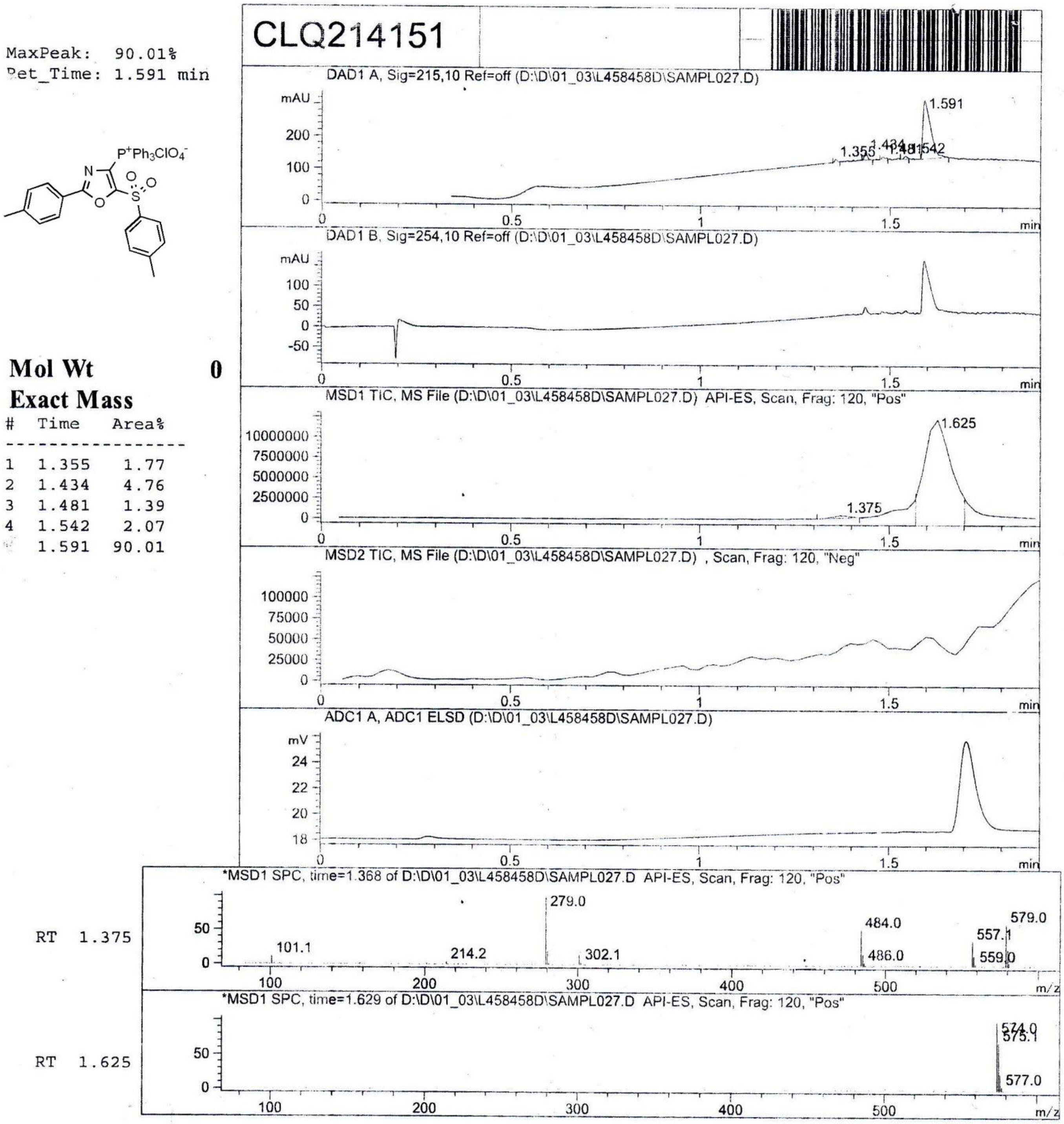

Figure S53. LCMS spectrum of compound (**11**).

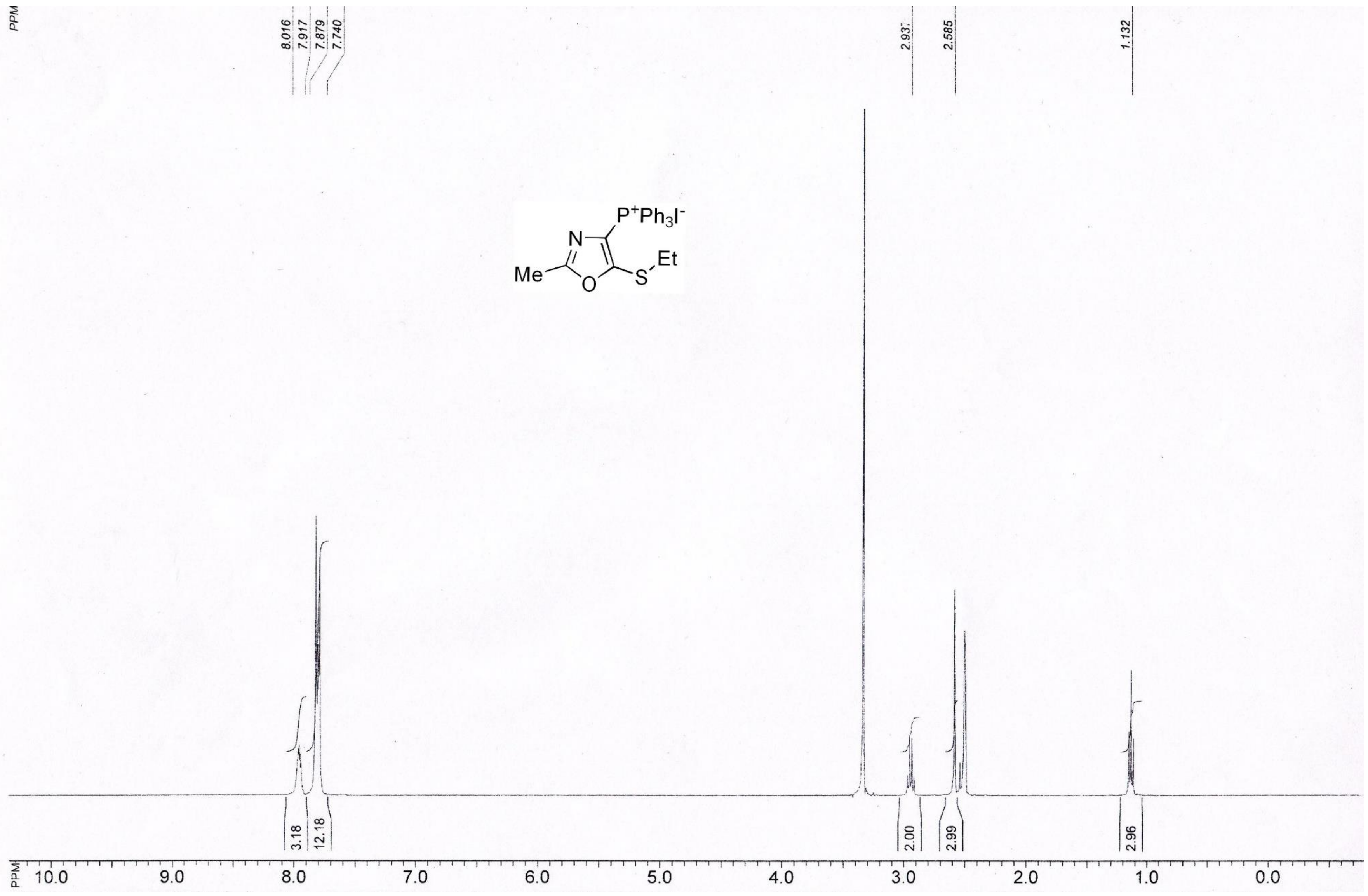

Figure S54. ^{1}H NMR (400 MHz, 298 K, DMSO-d_6) spectrum of compound (**12**).

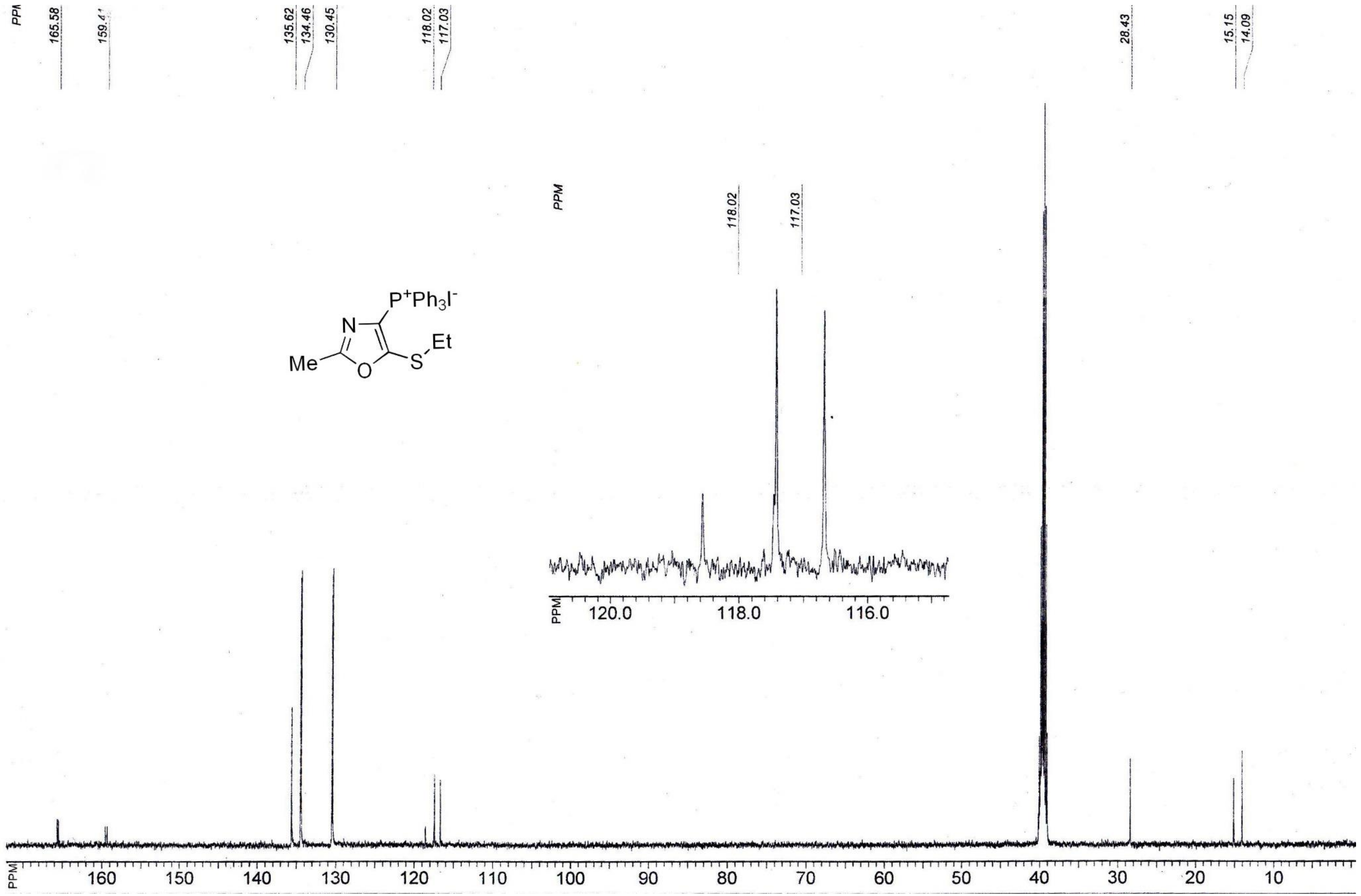

Figure S55. ^{13}C NMR (126 MHz, 683 K, DMSO-d_6) spectrum of compound (**12**).

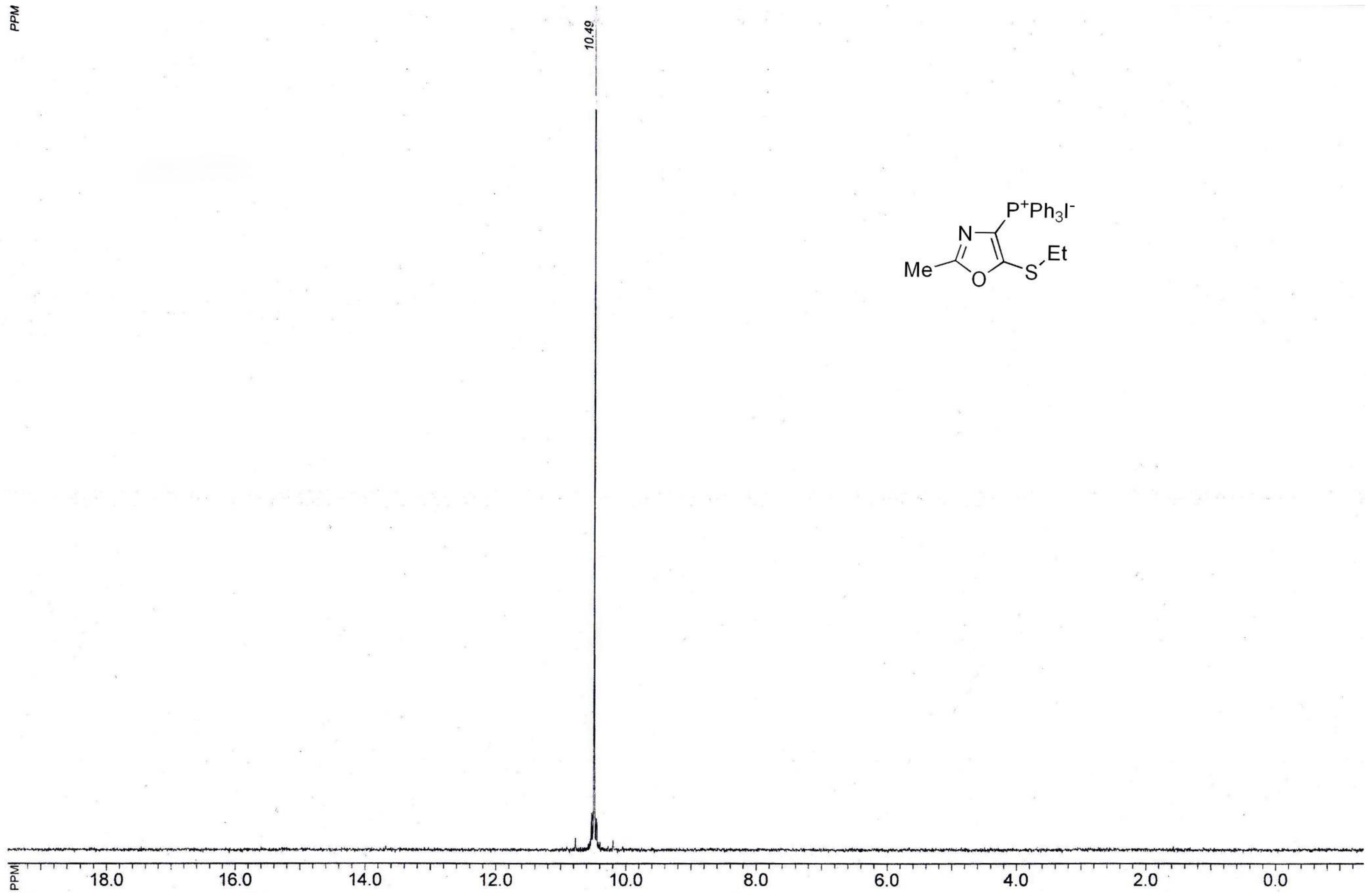

Figure S56. ^{31}P NMR (162 MHz, 294 K, DMSO-d_6) spectrum of compound (**12**).

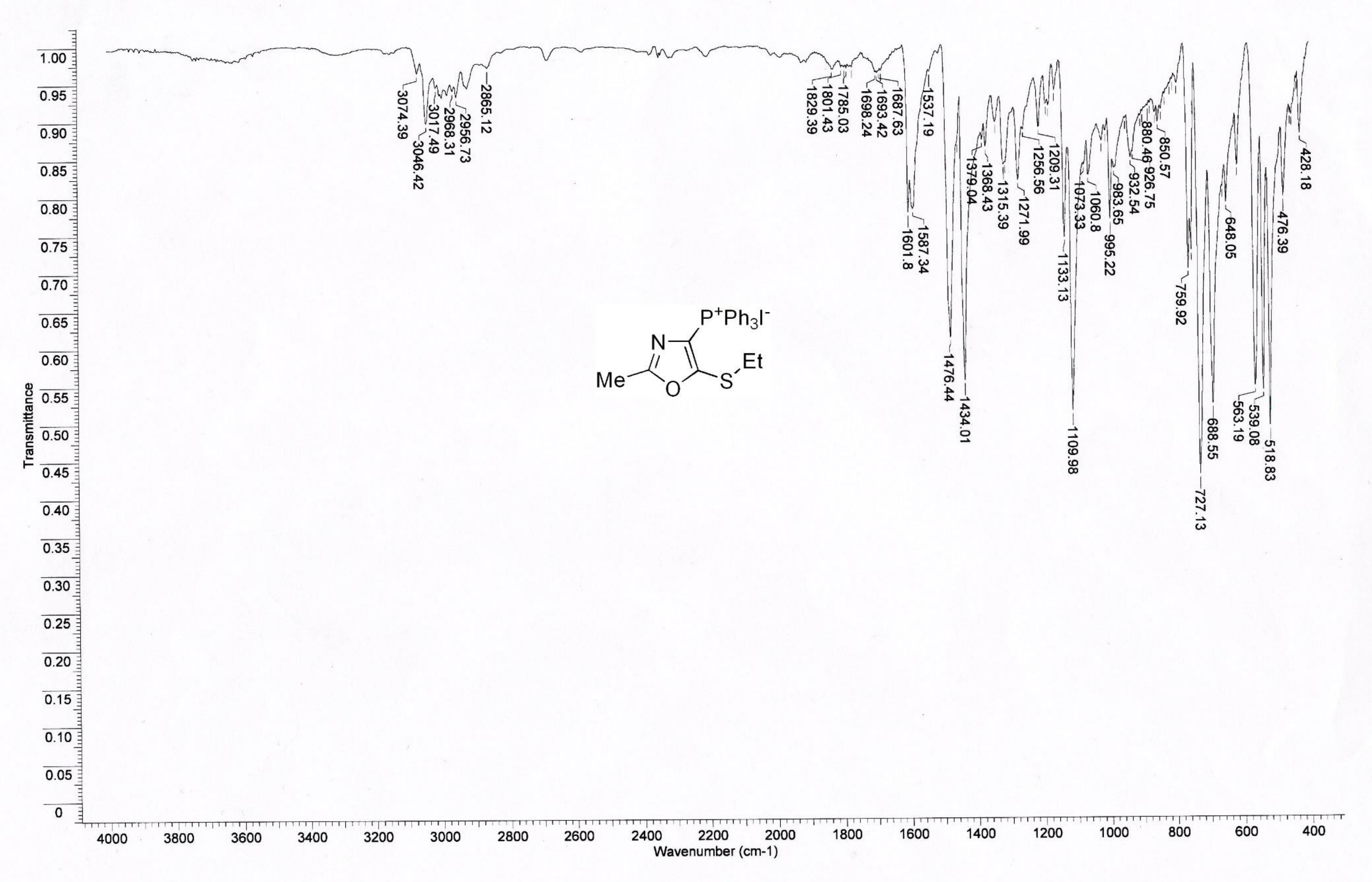

Figure S57. IR spectrum of compound (**12**).

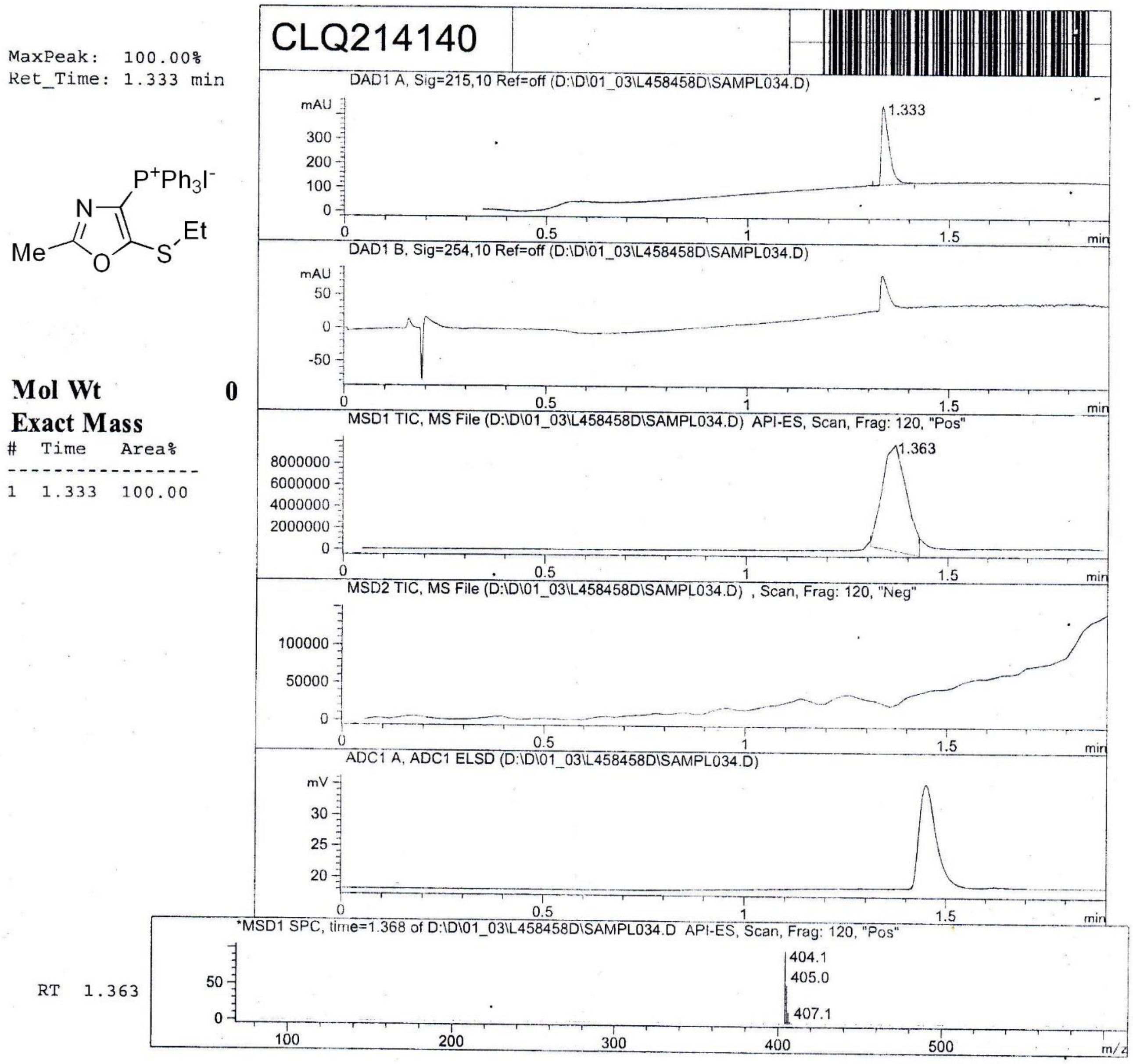

Figure S58. LCMS spectrum of compound (**12**).

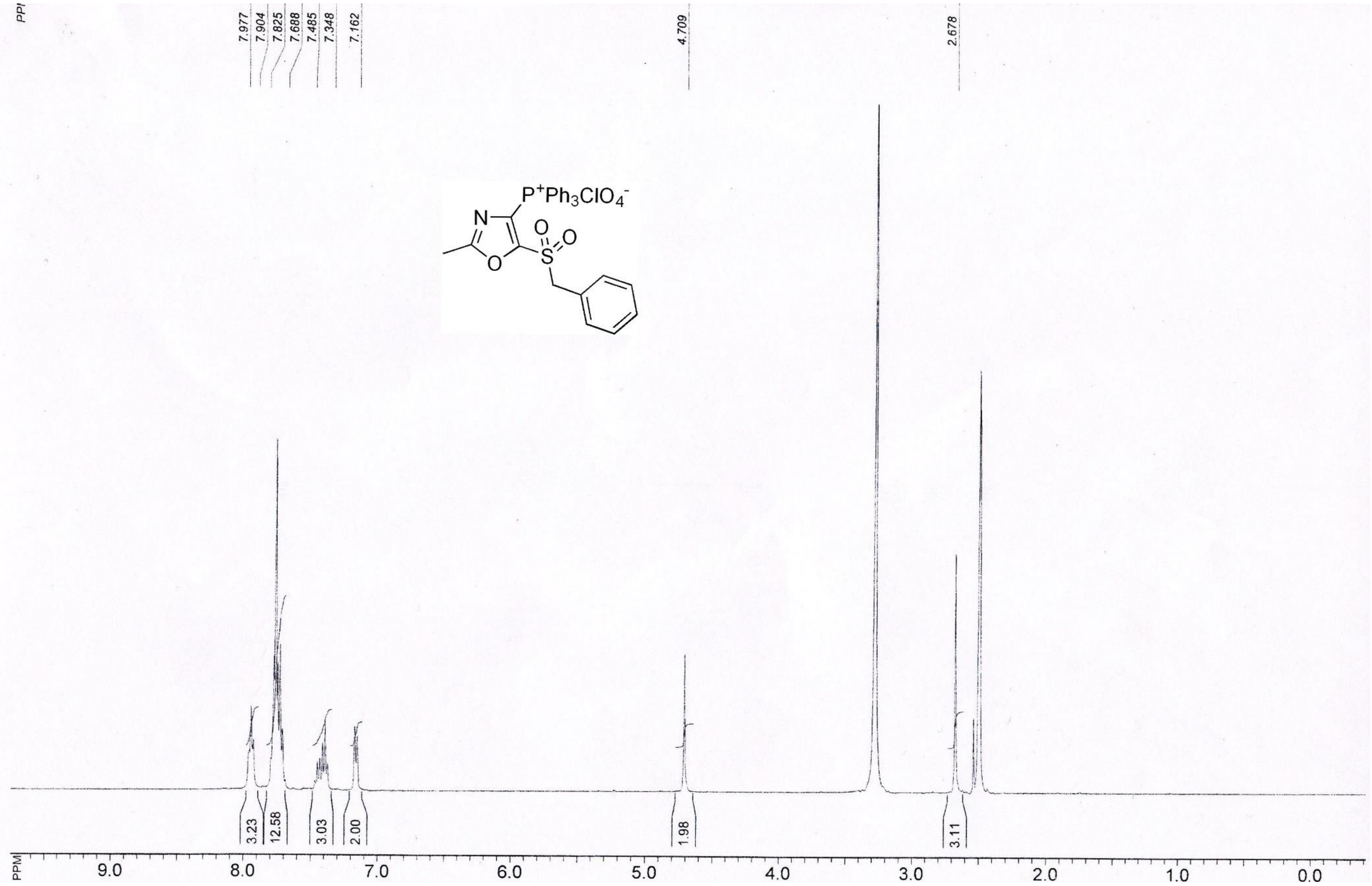

Figure S59. ^{1}H NMR (400 MHz, 298 K, DMSO-d_6) spectrum of compound (**13**).

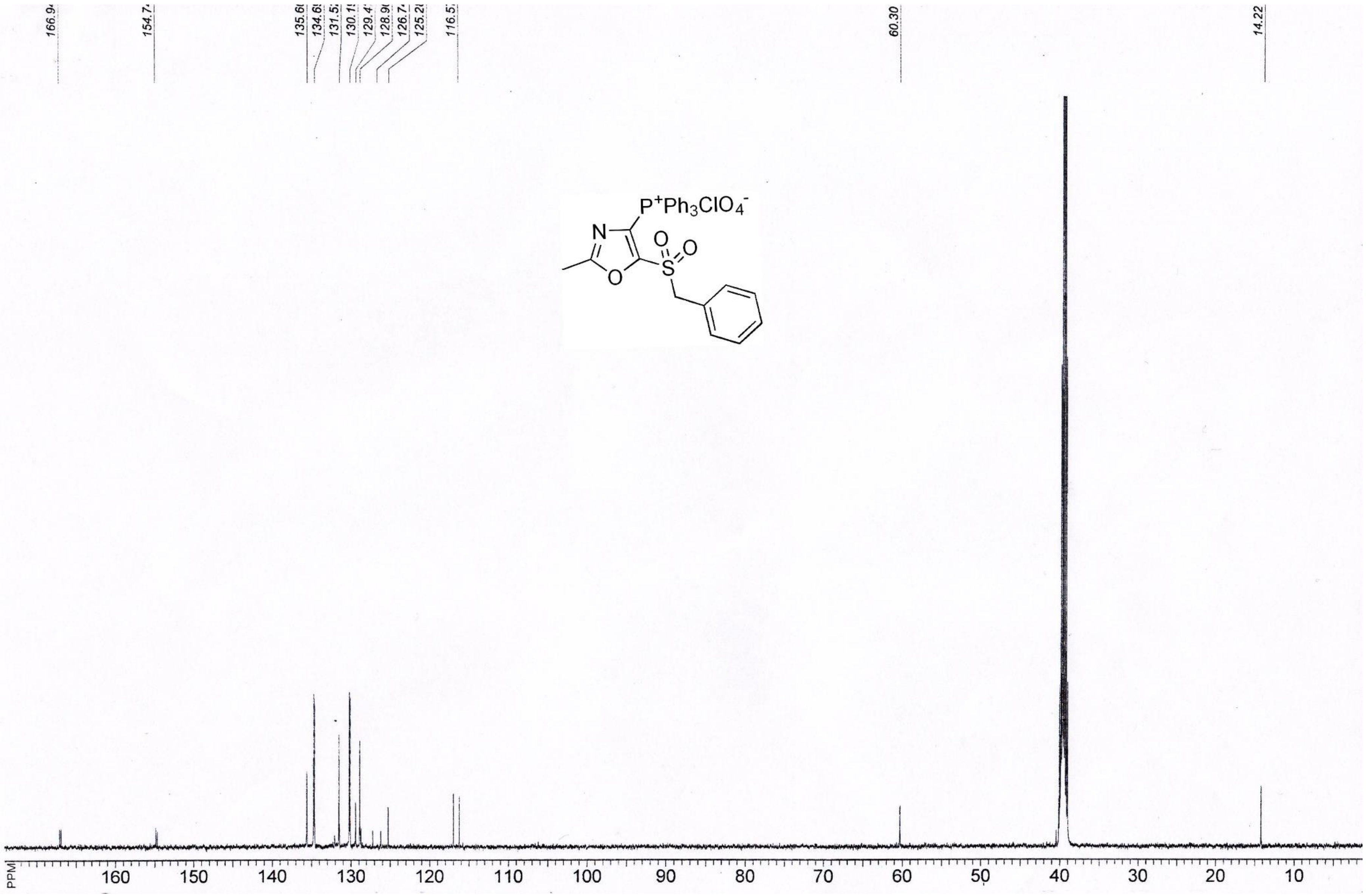

Figure S60. ^{13}C NMR (126 MHz, 683 K, DMSO-d_6) spectrum of compound (**13**).

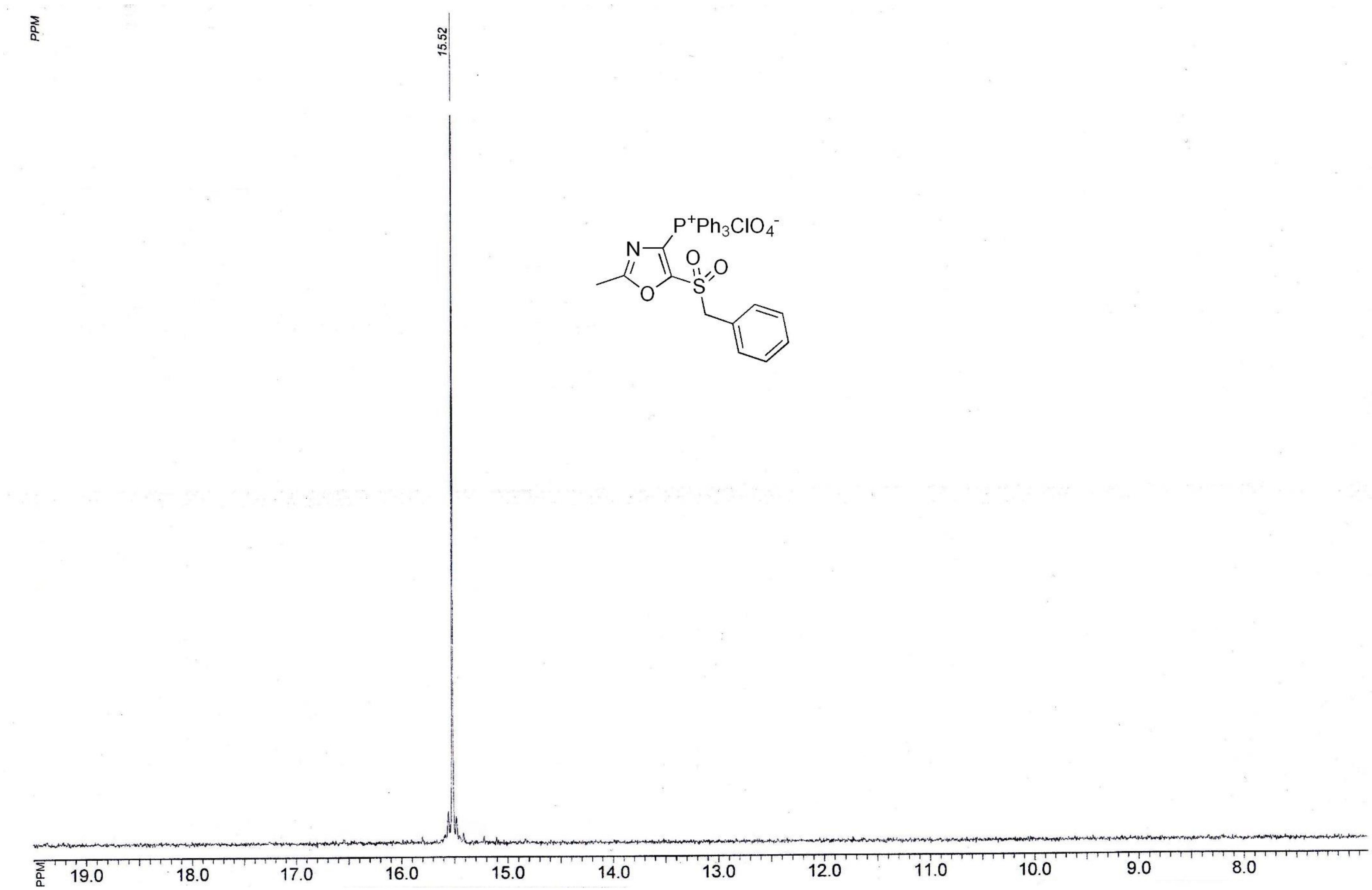

Figure S61. ^{31}P NMR (162 MHz, 294 K, DMSO-d_6) spectrum of compound (**13**).

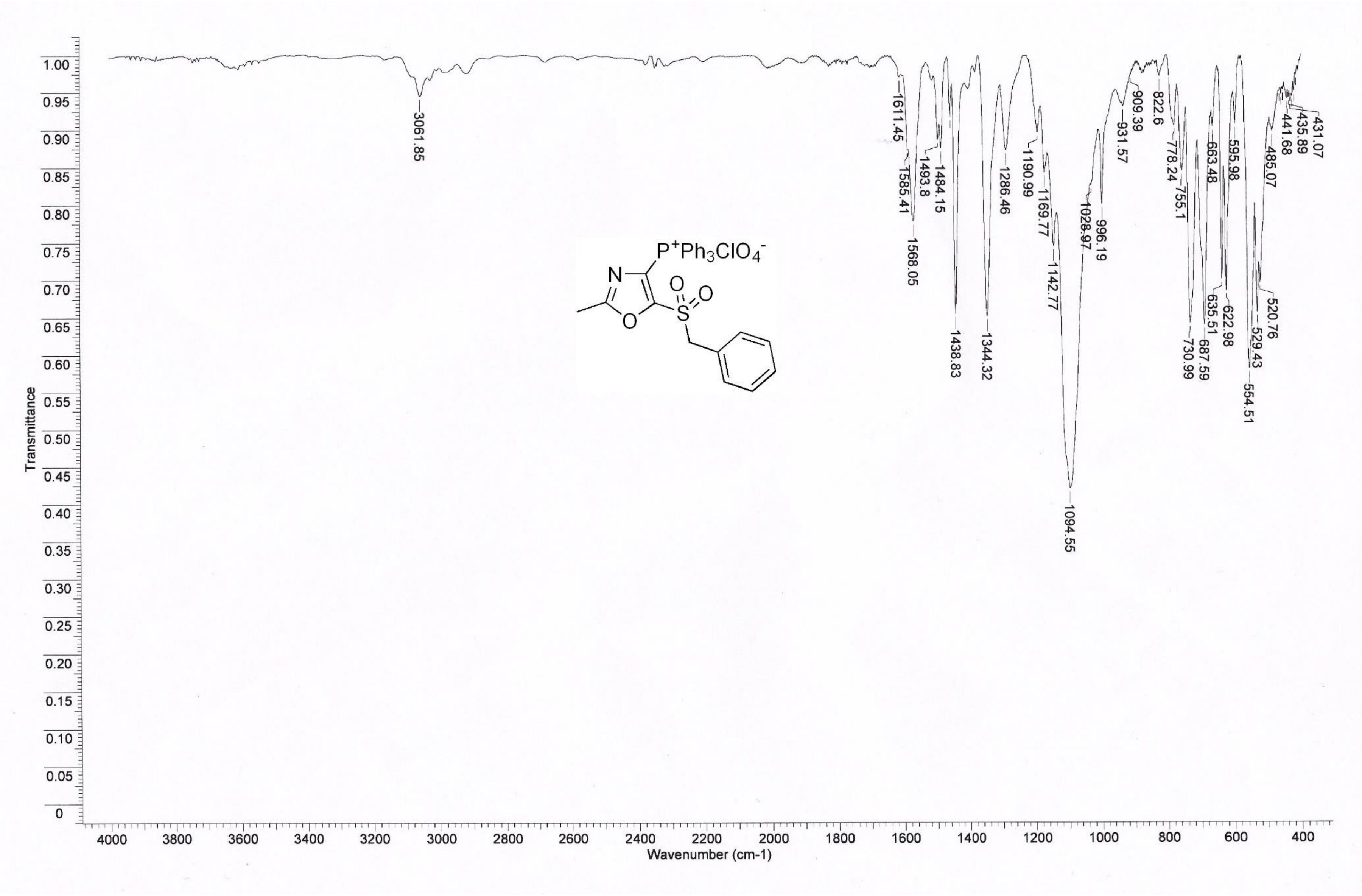

Figure S62. IR spectrum of compound (**13**).

MaxPeak: 77.17%
Ret_Time: 1.310 min

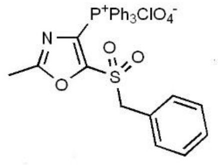

Mol Wt 0
Exact Mass

#	Time	Area%
1	1.035	0.67
2	1.169	1.95
3	1.273	4.63
4	1.310	77.17
5	1.342	14.42
6	1.400	1.16

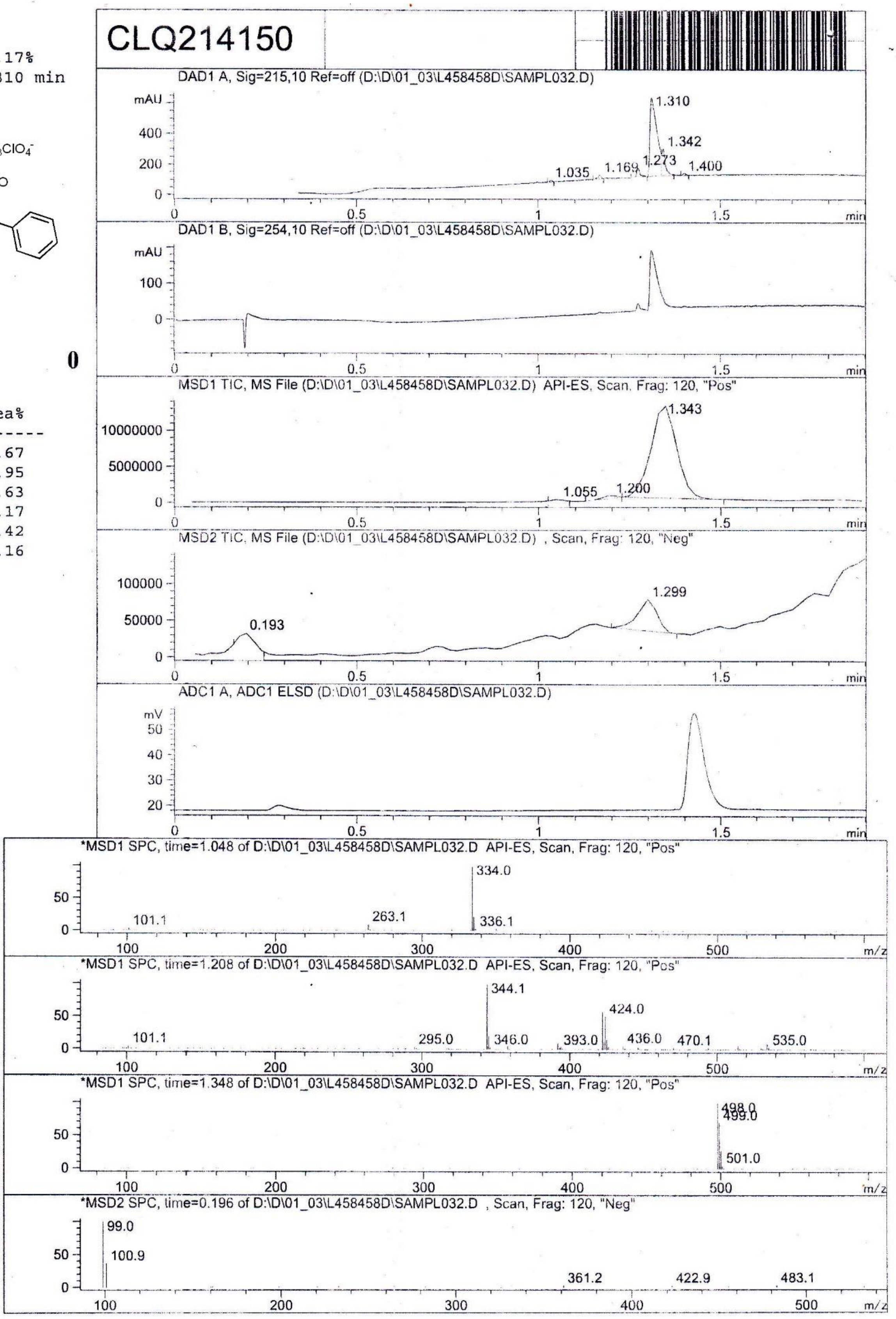

RT 1.055

RT 1.200

RT 1.343

RT 0.193

Figure S63. LCMS spectrum of compound (**13**).

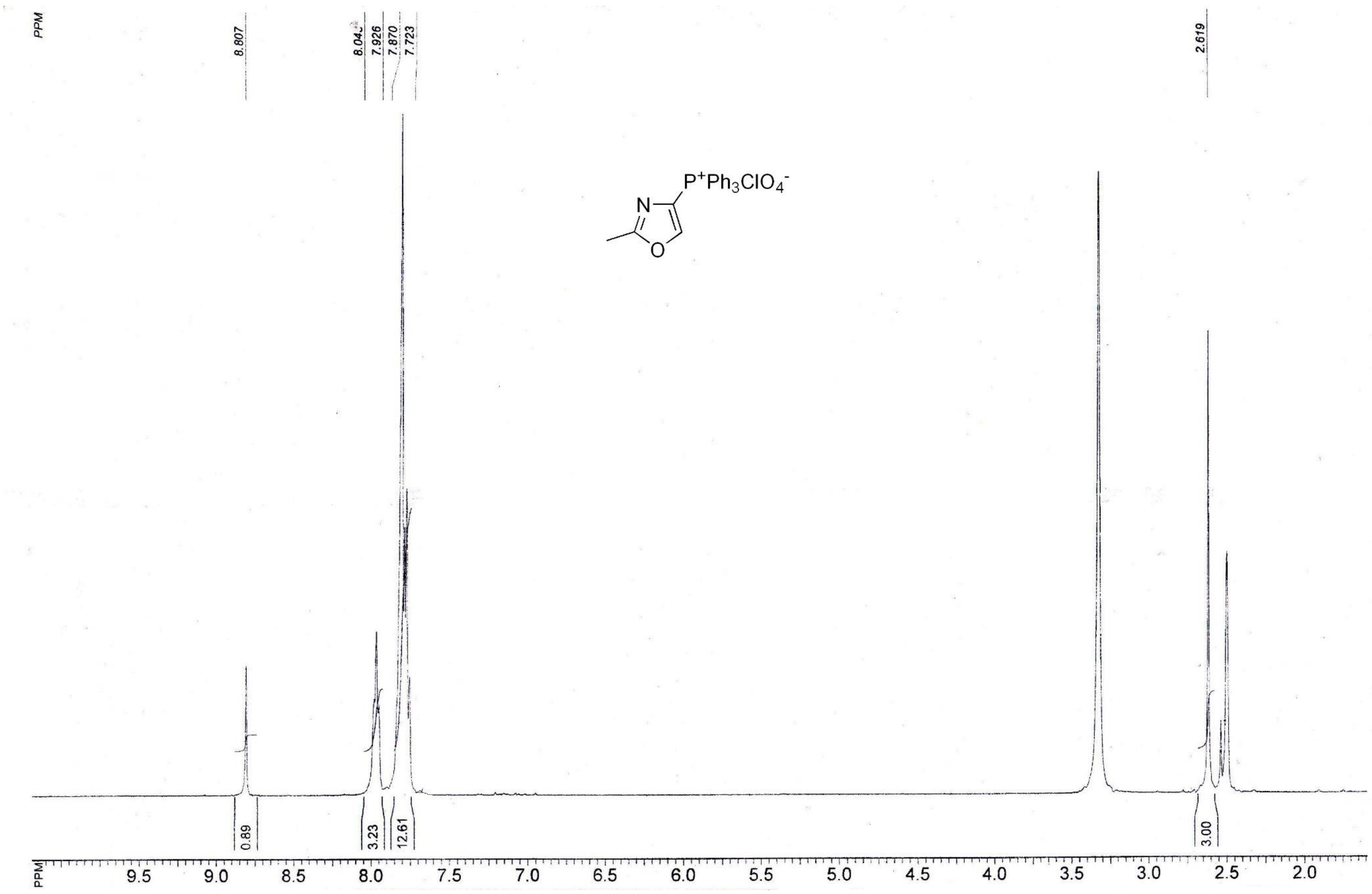

Figure S64. ^{1}H NMR (400 MHz, 298 K, DMSO-d_6) spectrum of compound (**14**).

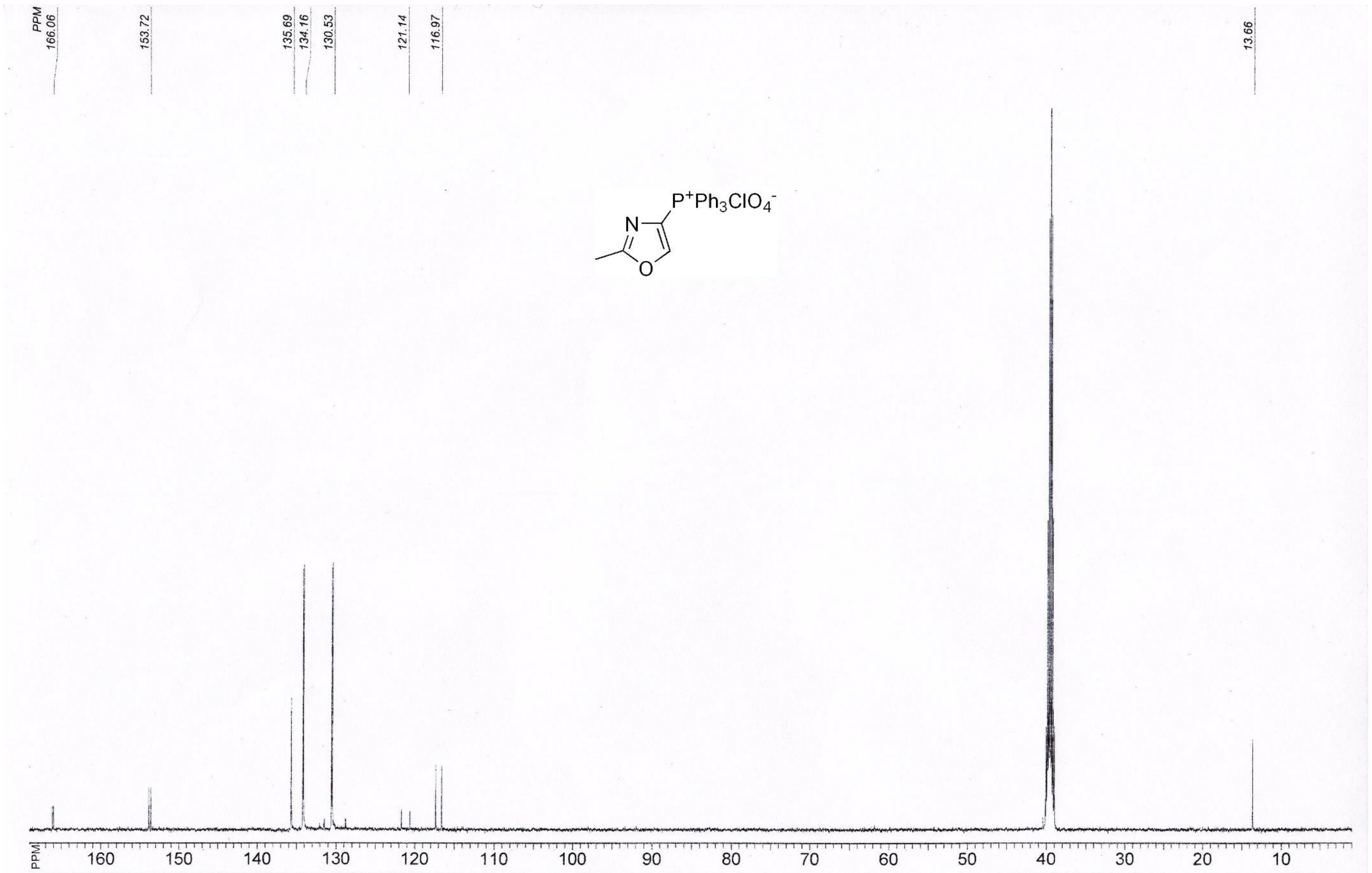

Figure S65. ^{13}C NMR (126 MHz, 683 K, DMSO-d_6) spectrum of compound (**14**).

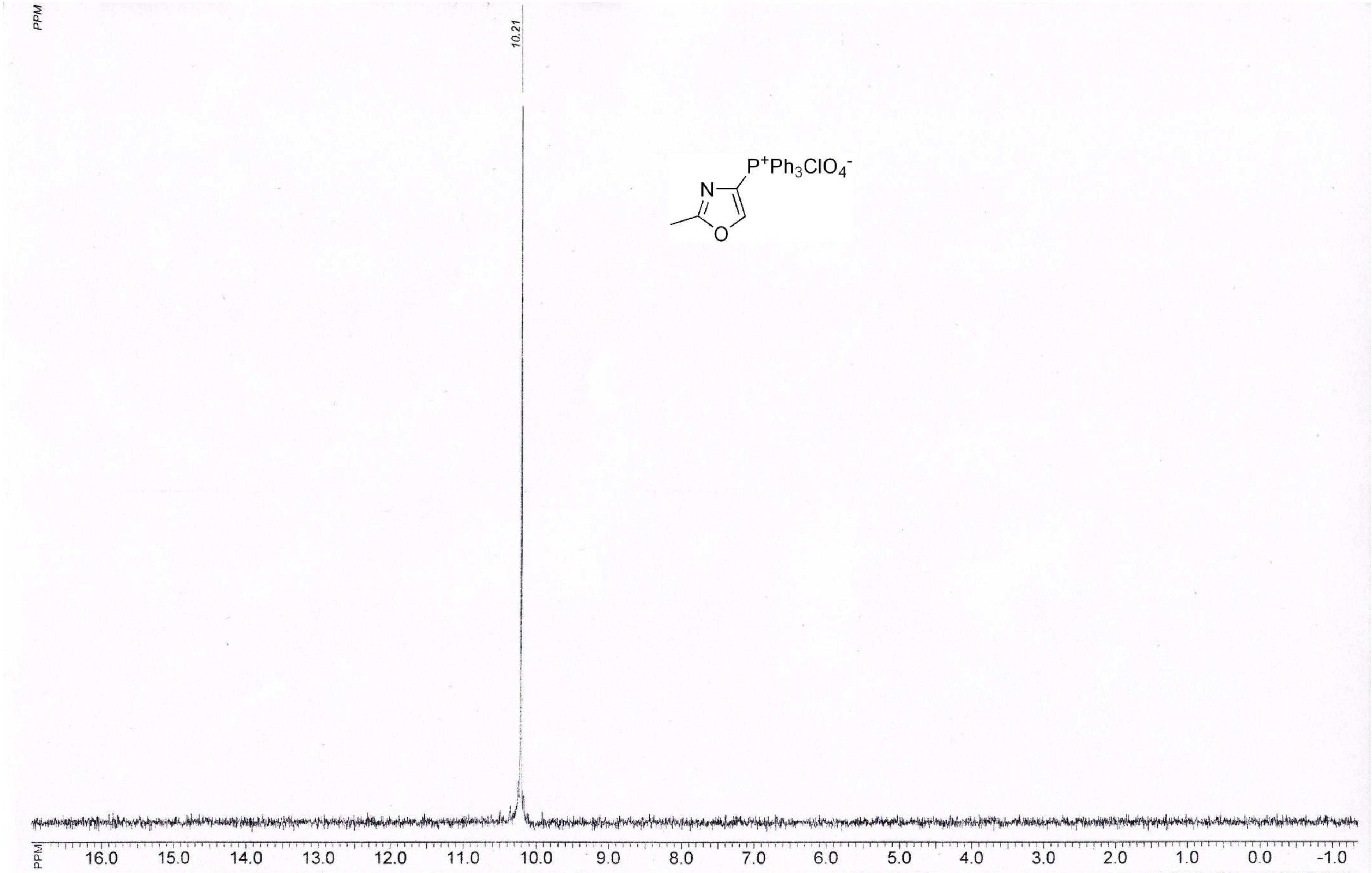

Figure S66. ^{31}P NMR (162 MHz, 294 K, DMSO-d_6) spectrum of compound (**14**).

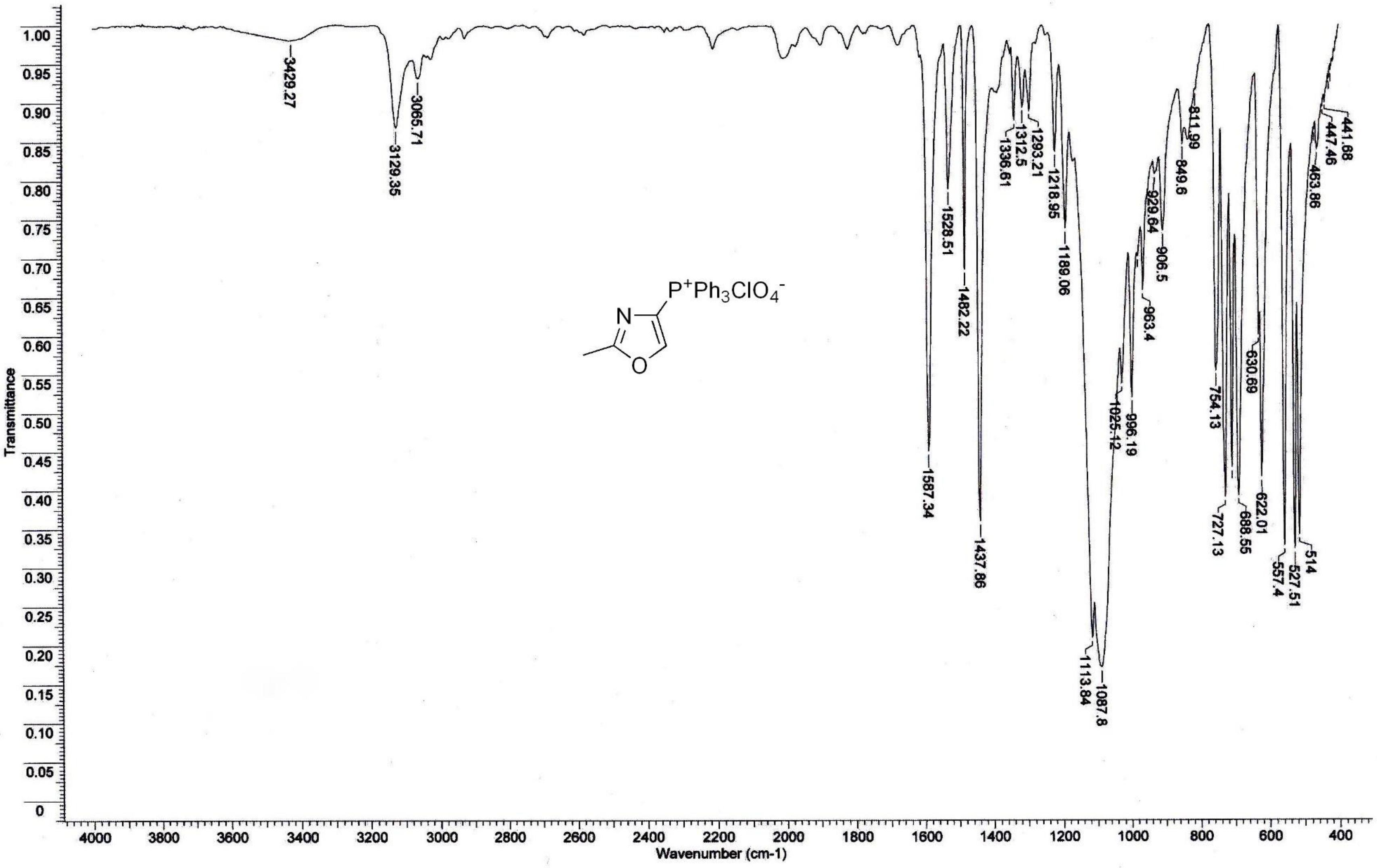

Figure S67. IR spectrum of compound (**14**).

MaxPeak: 93.44%
Ret_Time: 1.052 min

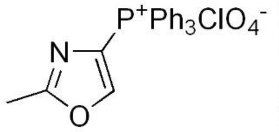

Mol Wt 0
Exact Mass

#	Time	Area%
1	0.937	1.24
2	1.024	2.44
3	1.052	93.44
4	1.279	2.88

RT 0.947

RT 1.071

RT 1.291

RT 0.186

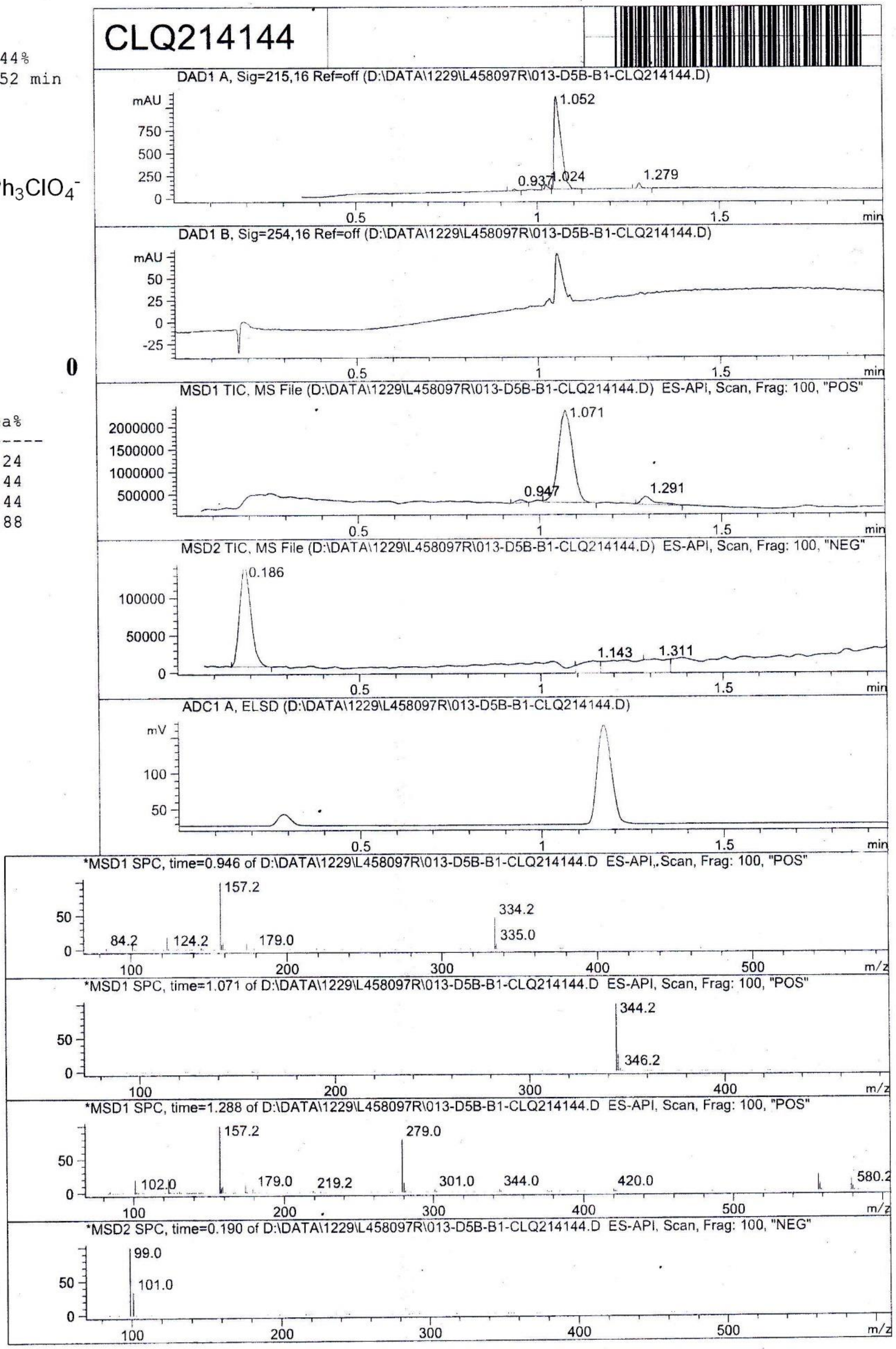

Figure S68. LCMS spectrum of compound (**14**).

Table S1. Data from an one-dose (10^{-5} µM) antitumor screening of synthesized compounds against human tumor cell lines NCI-60, whose growth was inhibited by more than 50%.

**Compound**	**Leukemia**	**Non-Small Cell Lung cancer**	**Colon cancer**	**CNS cancer**	**Melanoma**	**Ovarian cancer**	**Renal cancer**	**Prostate Cancer**	**Breast cancer**
**1**	CCRF-CEM (94)<br>HL-60(TB) (133)<br>K-562 (99)<br>MOLT-4 (102)<br>RPMI-8226 (72)<br>SR (91)	A549/ATCC (125)<br>EKVX (131)<br>HOP-62 (151)<br>HOP-92 (145)<br>NCI-H226 (138)<br>NCI-H23 (152)<br>NCI-H322M (122)<br>NCI-H460 (182)<br>NCI-H522 (157)	COLO 205 (135)<br>HCC-2998 (189)<br>HCT-116 (166)<br>HCT-15 (134)<br>HT29 (128)<br>KM12 (151)<br>SW-620 (148)	SF-268 (89)<br>SF-295 (185)<br>SF-539 (161)<br>SNB-19 (100)<br>SNB-75 (102)<br>U251 (158)	LOX IMVI (200)<br>MALME-3M (162)<br>M14 (176)<br>MDA-MB-435 (163)<br>SK-MEL-2 (169)<br>SK-MEL-28 (99)<br>SK-MEL-5 (200)<br>UACC-257 (143)<br>UACC-62 (135)	IGROV1 (112)<br>OVCAR-3 (131)<br>OVCAR-4 (93)<br>OVCAR-5 (89)<br>OVCAR-8 (127)<br>NCI/ADR-RES (152)<br>SK-OV-3 (133)	786-0 (177)<br>A498 (152)<br>ACHN (97)<br>CAKI-1 (130)<br>RXF 393 (169)<br>SN12C (92)<br>TK-10 (132)<br>UO-31 (173)	PC-3 (121)<br>DU-145 (119)	MCF7 (120)<br>MDA-MB-231/ATCC (183)<br>HS 578T (139)<br>BT-549 (121)<br>T-47D (129)<br>MDA-MB-468 (177)
**2**	CCRF-CEM (87)<br>HL-60(TB) (112)<br>K-562 (90)<br>MOLT-4 (92)<br>RPMI-8226 (129)	A549/ATCC (85)<br>EKVX (85)<br>HOP-62 (103)<br>HOP-92 (121)<br>NCI-H226 (65)<br>NCI-H23 (97)<br>NCI-H322M (79)<br>NCI-H460 (90)<br>NCI-H522 (103)	COLO 205 (165)<br>HCC-2998 (73)<br>HCT-116 (98)<br>HT29 (100)<br>KM12 (102)<br>SW-620 (86)	SF-268 (82)<br>SF-295 (100)<br>SF-539 (93)<br>SNB-19 (91)<br>SNB-75 (99)<br>U251 (90)	LOX IMVI (95)<br>MALME-3M (95)<br>M14 (95)<br>MDA-MB-435 (101)<br>SK-MEL-2 (125)<br>SK-MEL-28 (78)<br>SK-MEL-5 (193)<br>UACC-257 (109)<br>UACC-62 (137)	IGROV1 (83)<br>OVCAR-3 (89)<br>OVCAR-4 (89)<br>OVCAR-5 (89)<br>OVCAR-8 (98)<br>SK-OV-3 (102)	786-0 (73)<br>A498 (88)<br>ACHN (87)<br>RXF 393 (105)<br>SN12C (99)<br>TK-10 (70)	PC-3 (94)<br>DU-145 (81)	MCF7 (97)<br>MDA-MB-231/ATCC (115)<br>HS 578T (75)<br>BT-549 (105)<br>T-47D (103)<br>MDA-MB-468 (123)
**4**	CCRF-CEM (100)<br>HL-60(TB) (148)<br>K-562 (99)<br>MOLT-4 (109)<br>RPMI-8226 (150)	A549/ATCC (95)<br>EKVX (123)<br>HOP-62 (116)<br>HOP-92 (114)<br>NCI-H226 (80)	COLO 205 (162)<br>HCC-2998 (151)<br>HCT-116 (122)<br>HCT-15 (98)	SF-268 (89)<br>SF-295 (121)<br>SF-539 (177)<br>SNB-19 (98)	LOX IMVI (108)<br>MALME-3M (144)<br>M14 (140)<br>MDA-MB-435 (133)<br>SK-MEL-2 (159)	IGROV1 (85)<br>OVCAR-3 (112)<br>OVCAR-4 (105)<br>OVCAR-5 (91)	786-0 (125)<br>A498 (138)<br>ACHN (112)<br>CAKI-1 (128)	PC-3 (96)<br>DU-145 (87)	MCF7 (100)<br>MDA-MB-231/ATCC (137)<br>HS 578T (131)<br>BT-549 (98)<br>T-47D (92)

	SR (127)	NCI-H23 (116)<br>NCI-H322M (96)<br>NCI-H460 (145)<br>NCI-H522 (133)	HT29 (102)<br>KM12 (135)<br>SW-620 (130)	SNB-75 (132)<br>U251 (116)	SK-MEL-28 (142)<br>SK-MEL-5 (187)<br>UACC-257 (113)<br>UACC-62 (126)	OVCAR-8 (121)<br>NCI/ADR-RES (103)<br>SK-OV-3 (109)	RXF 393 (132)<br>SN12C (97)<br>TK-10 (92)<br>UO-31 (123)		MDA-MB-468 (142)
**5**	CCRF-CEM (92)<br>HL-60(TB) (128)<br>K-562 (95)<br>MOLT-4(108)<br>RPMI-8226 (1244)<br>SR (94)	A549/ATCC (90)<br>EKVX (82)<br>HOP-62 (142)<br>HOP-92 (129)<br>NCI-H226 (103)<br>NCI-H23 (110)<br>NCI-H322M (92)<br>NCI-H460 (93)<br>NCI-H522 (100)	COLO 205 (170)<br>HCC-2998 (86)<br>HCT-116 (98)<br>HT29 (101)<br>KM12 (122)<br>SW-620 (90)	SF-268 (87)<br>SF-295 (110)<br>SF-539 (81)<br>SNB-19 (89)<br>SNB-75 (100)<br>U251 (88)	MALME-3M (119)<br>M14 (100)<br>MDA-MB-435 (107)<br>SK-MEL-2 (119)<br>SK-MEL-28 (70)<br>SK-MEL-5 (195)<br>UACC-257 (105)<br>UACC-62 (129)	IGROV1 (99)<br>OVCAR-3 (100)<br>OVCAR-4 (88)<br>OVCAR-5 (80)<br>OVCAR-8 (96)<br>SK-OV-3 (144)	786-0 (84)<br>ACHN (92)<br>CAKI-1 (87)<br>RXF 393 (101)<br>SN12C (99)<br>TK-10 (79)<br>UO-31 (88)	PC-3 (119)<br>DU-145 (87)	MCF7 (97)<br>MDA-MB-231/ATCC (101)<br>HS 578T (83)<br>BT-549 (113)<br>T-47D (120)<br>MDA-MB-468 (128)
**6**	CCRF-CEM (79)<br>HL-60(TB) (103)<br>K-562 (84)<br>MOLT-4 (71)<br>RPMI-8226 (113)<br>SR (80)	A549/ATCC (77)<br>HOP-62 (77)<br>HOP-92 (73)<br>NCI-H23 (83)<br>NCI-H460 (83)<br>NCI-H522 (71)	COLO 205 (145)<br>HCC-2998 (68)<br>HCT-116 (89)<br>HT29 (86)<br>KM12 (90)<br>SW-620 (63)	SF-268 (74)<br>SF-295 (67)<br>SF-539 (54)<br>SNB-19 (81)<br>SNB-75 (62)<br>U251 (83)	LOX IMVI (75)<br>M14 (63)<br>MDA-MB-435 (79)<br>SK-MEL-2 (59)<br>SK-MEL-28 (66)<br>SK-MEL-5 (132)<br>UACC-257 (92)<br>UACC-62 (75)	IGROV1 (67)<br>OVCAR-3 (81)<br>OVCAR-4 (80)<br>OVCAR-8 (80)<br>SK-OV-3 (79)	A498 (65)<br>SN12C (65)	PC-3 (85)<br>DU-145 (70)	MCF7 (89)<br>MDA-MB-231/ATCC (57)<br>HS 578T (72)<br>BT-549 (80)<br>T-47D (100)<br>MDA-MB-468 (100)
**8**	CCRF-CEM (89)<br>HL-60(TB) (135)<br>K-562 (93)<br>MOLT-4 (99)<br>RPMI-8226 (119)	A549/ATCC (91)<br>EKVX (89)<br>HOP-62 (100)<br>HOP-92 (128)<br>NCI-H226 (103)	COLO 205 (145)<br>HCC-2998 (85)<br>HCT-116 (98)<br>HCT-15 (94)<br>HT29 (99)	SF-268 (86)<br>SF-295 (104)<br>SF-539 (85)<br>SNB-19 (88)	LOX IMVI (185)<br>MALME-3M (125)<br>M14 (92)<br>MDA-MB-435 (97)<br>SK-MEL-2 (110)	IGROV1 (95)<br>OVCAR-3 (97)<br>OVCAR-4 (87)<br>OVCAR-5 (72)	786-0 (87)<br>A498 (133)<br>ACHN (105)<br>CAKI-1 (99)<br>RXF 393 (100)	PC-3 (102)<br>DU-145 (89)	MCF7 (95)<br>MDA-MB-231/ATCC (113)<br>HS 578T (82)<br>BT-549 (111)<br>T-47D (98)

	SR (99)	NCI-H23 (112)<br>NCI-H322M (106)<br>NCI-H460 (99)<br>NCI-H522 (120)	KM12 (116)<br>SW-620 (89)	SNB-75 (93)<br>U251 (85)	SK-MEL-28 (70)<br>SK-MEL-5 (192)<br>UACC-257 (92)<br>UACC-62 (100)	OVCAR-8 (92)<br>SK-OV-3 (116)	SN12C (97)<br>TK-10 (76)<br>UO-31 (131)		MDA-MB-468 (138)
**9**	CCRF-CEM (102)<br>HL-60(TB) (145)<br>K-562 (95)<br>MOLT-4 (126)<br>RPMI-8226 (138)<br>SR (118)	A549/ATCC (92)<br>EKVX (93)<br>HOP-62 (90)<br>HOP-92 (109)<br>NCI-H226 (84)<br>NCI-H23 (117)<br>NCI-H322M (90)<br>NCI-H460 (140)<br>NCI-H522 (139)	COLO 205 (141)<br>HCC-2998 (98)<br>HCT-116 (98)<br>HCT-15 (99)<br>HT29 (99)<br>KM12 (153)<br>SW-620 (105)	SF-268 (99)<br>SF-295 (90)<br>SF-539 (105)<br>SNB-19 (86)<br>SNB-75 (124)<br>U251 (87)	LOX IMVI (169)<br>MALME-3M (141)<br>M14 (93)<br>MDA-MB-435 (130)<br>SK-MEL-2 (136)<br>SK-MEL-28 (83)<br>SK-MEL-5 (198)<br>UACC-257 (99)<br>UACC-62 (138)	IGROV1 (174)<br>OVCAR-3 (103)<br>OVCAR-4 (103)<br>OVCAR-5 (92)<br>OVCAR-8 (95)<br>NCI/ADR-RES (98)<br>SK-OV-3 (103)	786-0 (96)<br>A498 (141)<br>ACHN - 6.75<br>CAKI-1 (123)<br>RXF 393 (119)<br>SN12C (96)<br>TK-10 (77)<br>UO-31 (122)	PC-3 (96)<br>DU-145 (88)	MCF7 (124)<br>MDA-MB-231/ATCC (113)<br>HS 578T (108)<br>BT-549 (131)<br>T-47D 1.57<br>MDA-MB-468 (137)
**10**	HL-60(TB) (53)<br>K-562 (69)<br>RPMI-8226 (94)<br>SR (69)	NCI-H23 (70)<br>NCI-H460 (63)	COLO 205 (93)<br>HCC-2998 (57)<br>HCT-116 (67)<br>KM12 (77)	SNB-19 (55)<br>U251 (58)	LOX IMVI (71)<br>MDA-MB-435 (59)<br>SK-MEL-5 (109)<br>UACC-257 (79)<br>UACC-62 (53)	OVCAR-3 (63)<br>OVCAR-4 (65)	–	–	MCF7 (77)<br>BT-549 (57)<br>T-47D (91)<br>MDA-MB-468 (104)
**11**	CCRF-CEM (107)<br>HL-60(TB) (114)<br>K-562 (96)<br>MOLT-4 (103)<br>RPMI-8226 (128)<br>SR (111)	A549/ATCC (73)<br>EKVX (77)<br>HOP-62 (82)<br>HOP-92 (108)<br>NCI-H226 (53)<br>NCI-H23 (85)<br>NCI-H460 (95)	COLO 205 (138)<br>HCC-2998 (62)<br>HCT-116 (169)<br>HCT-15 (96)<br>HT29 (100)<br>KM12 (81)<br>SW-620 (170)	SF-268 (55)<br>SF-539 (166)<br>SNB-75 (111)<br>U251 (97)	MALME-3M (101)<br>M14 (81)<br>MDA-MB-435 (103)<br>SK-MEL-2 (78)<br>SK-MEL-28 (75)<br>SK-MEL-5 (51)<br>UACC-257 (96)	IGROV1 (74)<br>OVCAR-3 (100)<br>OVCAR-4 (76)<br>OVCAR-5 (75)<br>OVCAR-8 (92)	786-0 (119)<br>A498 (101)<br>ACHN (113)<br>CAKI-1 (94)<br>RXF 393 (129)<br>SN12C (96)<br>TK-10 (97)	PC-3 (89)<br>DU-145 (75)	MCF7 (87)<br>MDA-MB-231/ATCC (97)<br>HS 578T (110)<br>BT-549 (91)<br>T-47D (81)<br>MDA-MB-468 (121)

		NCI-H522 (96)			UACC-62 (74)		UO-31 (85)		
**12**	CCRF-CEM (62)<br>HL-60(TB) (93)<br>K-562 (80)<br>MOLT-4 (62)<br>RPMI-8226 (95)<br>SR (64)	A549/ATCC (77)<br>EKVX (74)<br>HOP-62 (79)<br>HOP-92 (75)<br>NCI-H23 (79)<br>NCI-H460 (83)<br>NCI-H522 (75)	COLO 205 (107)<br>HCC-2998 (71)<br>HCT-116 (86)<br>HT29 (83)<br>KM12 (92)<br>SW-620 (66)	SF-268 (75)<br>SF-295 (65)<br>SNB-19 (81)<br>SNB-75 (65)<br>U251 (80)	LOX IMVI (74)<br>MALME-3M (69)<br>M14 (67)<br>MDA-MB-435 (75)<br>SK-MEL-2 (50)<br>SK-MEL-5 (98)<br>UACC-257 (88)<br>UACC-62 (71)	IGROV1 30.31<br>OVCAR-3 15.81<br>OVCAR-4 38.24<br>OVCAR-5 69.23<br>OVCAR-8 21.47<br>NCI/ADR-RES 97.80<br>SK-OV-3 28.04	A498 (81)<br>SN12C (72)<br>TK-10 (52)	–	MCF7 (86)<br>MDA-MB-231/ATCC (51)<br>BT-549 (88)<br>T-47D (85)<br>MDA-MB-468 (120)

Table S2. Values of the anticancer activity parameters (GI_{50}, TGI and LC_{50}, μM) for the most active compounds against the NCI 60 human cancer cell lines (five-dose assay).

Cell Line	Compound																				
	**1**			**2**			**4**			**5**			**8**			**9**			**11**		
	**GI_{50}**	**TGI**	**LC_{50}**	**GI_{50}**	**TGI**	**LC_{50}**	**GI50**	**TGI**	**LC_{50}**	**GI_{50}**	**TGI**	**LC_{50}**	**GI_{50}**	**TGI**	**LC_{50}**	**GI_{50}**	**TGI**	**LC_{50}**	**GI_{50}**	**TGI**	**LC_{50}**
Leukemia																					
CCRF-CEM	0.40	2.24	22.9	0.99	85.4	>100	0.43	2.17	8.74	0.48	9.49	>100	0.37	2.88	>100	0.30	1.61	>100	0.61	6.94	>100
HL-60(TB)	0.25	0.61	4.39	0.34	1.50	>100	0.25	0.73	6.39	0.25	0.70	10.9	0.23	0.55	nd	0.22	0.54	36.8	1.67	4.38	41.2
K-562	0.29	1.18	6.63	0.42	>100	>100	0.31	1.15	4.74	0.32	3.58	>100	0.22	2.88	>100	0.29	1.52	>100	0.31	3.22	>100
MOLT-4	0.41	1.83	7.95	2.17	22.0	>100	0.50	2.03	6.94	0.49	3.13	37.9	0.40	1.76	nd	0.33	1.18	9.35	1.06	4.63	41.4
RPMI-8226nd	0.21	0.52	3.36	0.27	1.43	>100	0.18	0.55	8.65	0.20	0.69	77.7	0.17	0.55	>100	nd	nd	nd	nd	nd	nd
SR	0.39	1.80	>100	0.60	26.8	>100	0.36	1.71	13.6	0.41	5.64	>100	0.38	24.2	>100	nd	nd	nd	nd	nd	nd
Non-Small Cell Lung Cancer																					
A549/ATCC	0.61	2.12	5.50	2.49	46.4	>100	1.31	3.13	7.49	0.43	11.8	61.0	0.31	10.8	52.6	0.31	1.24	4.06	2.00	5.14	17.3
EKVX	0.37	1.69	5.18	1.84	34.9	>100	0.94	2.37	5.72	0.97	8.50	47.6	0.35	4.37	30.9	0.27	1.20	4.47	1.21	2.98	7.32
HOP-62	1.26	3.05	7.40	1.82	14.8	>100	1.35	3.45	nd	0.59	5.61	41.0	0.30	2.52	55.2	0.44	1.79	nd	1.47	4.56	28.1
HOP-92	0.18	0.81	3.09	0.38	3.08	35.0	0.12	0.92	3.73	0.32	2.24	24.2	0.13	0.52	10.9	0.14	0.61	3.85	1.43	3.13	6.81
NCI-H226	nd	nd	nd	nd	nd	nd	1.33	3.22	nd	nd	nd	nd	nd	nd	nd	0.41	1.90	nd	5.05	24.6	>100
NCI-H23	0.39.	1.53	5.41	1.91	6.54	94.9	0.36	1.46	5.00	0.45	5.80	37.5	0.29	1.37	35.6	0.31	1.10	6.25	1.68	3.50	7.31
NCI-H322M	1.13	2.41	5.14	2.86	13.1	>100	1.01	2.18	4.74	1.12	7.87	31.6	0.32	2.72	23.5	0.43	1.70	4.68	3.18	12.6	35.4
NCI-H460	0.46	1.62	4.38	2.02	11.1	85.3	0.47	1.69	4.68	0.38	10.2	37.5	0.29	1.05	8.69	0.31	1.11	4.30	1.64	3.43	7.20
NCI-H522	0.31	1.35	5.01	0.47	3.93	82.1	0.24	1.23	6.61	0.37	4.94	49.2	0.27	1.29	8.68	0.15	0.38	0.97	1.60	3.67	8.39
Colon Cancer																					
COLO 205	nd	nd	nd	nd	nd	nd	0.74	2.74	nd	nd	nd	nd	0.39	2.72	21.7	0.24	0.69	nd	1.71	4.28	>100
HCC-2998	0.76	2.03	4.63	0.68	10.9	35.6	0.59	1.97	4.91	0.42	4.07	26.2	0.31	10.1	40.0	0.40	1.64	4.84	1.45	3.01	6.24
HCT-116	0.35	1.20	3.52	0.82	8.40	8.22	0.35	1.38	nd	0.35	10.7	40.8	1.12	10.4	40.0	0.32	1.21	nd.	1.37	2.75	5.51
HCT-15	0.37	1.45	4.64	7.85	6.74	>100	0.50	1.87	4.97	2.81	11.0	39.6	0.29	3.78	50.8	0.29	1.22	4.74	1.44	3.23	7.25
HT29	0.31	1.14	6.17	0.53	11.1	>100	0.36	1.64	>100	0.35	10.7	86.8	0.29	1.08	3.79	0.29	1.15	5.90	1.69	4.17	11.9

KM12	0.50	1.77	4.64	0.38	1.83	15.0	0.53	1.87	4.70	0.37	1.69	12.6	0.31	10.5	39.2	0.34	1.34	5.00	1.75	3.83	8.37
SW-620	0.35	1.37	4.12	0.68	22.9	>100	0.52	1.94	5.03	0.43	11.2	75.3	nd	nd	nd	0.33	1.36	4.48	0.93	2.47	6.28
CNS Cancer																					
SF-268	1.13	2.53	5.69	0.59	11.4	52.2	1.25	2.58	5.31	0.41	11.4	46.4	0.36	11.5	51.7	0.41	1.68	5.42	1.55	2.97	5.67
SF-295	1.15	2.41	5.06	1.98	5.46	41.8	1.04	2.24	4.80	1.10	3.73	14.8	0.26	0.77	20.2	0.29	1.03	3.43	2.91	10.7	32.7
SF-539	1.55	2.93	5.52	1.53	10.1	37.6	1.54	2.90	5.47	0.50	7.72	31.2	0.33	3.61	29.0	0.38	1.49	3.88	1.58	2.92	5.41
SNB-19	0.66	1.95	4.64	0.42	14.8	>100	1.10	2.32	4.87	0.30	2.01	26.8	0.31	10.1	36.5	0.39	1.50	4.22	1.64	3.30	6.64
SNB-75	1.18	2.46	5.10	0.28	1.81	20.8	1.11	2.40	5.21	0.19	0.80	10.0	0.17	0.65	14.1	0.15	0.85	3.27	1.21	4.41	18.8
U251	0.23	1.08	3.42	0.49	11.6	80.3	0.66	2.37	6.66	0.29	11.4	42.2	0.22	11.0	43.9	0.32	1.40	4.47	1.14	2.43	5.18
Melanoma																					
LOX IMVI	0.61	1.89	4.44	1.13	2.74	6.63	0.42	1.75	4.59	0.49	2.31	9.22	0.21	0.53	1.98	0.16	0.32	0.63	0.33	1.30	4.14
MALME-3M	0.28	0.99	3.81	0.44	4.11	29.6	0.28	1.17	3.70	0.31	1.78	20.9	0.27	1.23	4.10	0.26	0.92	3.11	1.02	2.19	4.74
M14	0.41	1.59	4.20	1.27	10.1	47.7	nd	nd	nd	0.50	10.4	59.6	0.35	2.40	32.6	0.39	1.49	4.49	1.51	3.27	7.08
MDA-MB-435	0.41	1.48	3.89	0.40	3.38	31.9	0.52	1.81	4.38	0.30	1.05	8.55	0.28	1.01	8.27	0.33	1.20	3.79	1.56	3.04	5.91
SK-MEL-2	0.33	1.24	4.58	0.89	3.83	25.5	1.04	2.78	7.43	0.58	2.66	12.2	0.27	0.88	7.87	1.12	2.63	6.13	2.17	4.41	8.99
SK-MEL-28	1.01	2.25	4.99	0.57	12.4	41.4	1.35	2.70	5.39	0.47	7.51	30.8	0.49	2.83	15.1	0.65	1.93	4.46	1.70	3.09	5.63
SK-MEL-5	0.28	0.93	3.15	0.53	1.78	4.45	0.43	1.55	4.03	0.30	0.88	3.07	0.17	0.33	6.44	0.43	1.54	4.00	1.81	3.40	6.40
UACC-257	0.41	1.64	4.63	0.37	2.64	>100	0.94	3.25	12.90	0.32	1.66	36.6	0.29	1.04	36.2	0.30	1.21	5.91	1.70	3.70	8.08
UACC-62	0.46	1.69	4.43	0.48	2.93	22.4	0.66	1.94	4.45	0.31	1.24	4.48	0.32	1.48	5.92	0.41	1.55	4.03	1.53	2.89	5.47
Ovarian Cancer																					
IGROV1	0.69	2.07	5.09	0.44	5.45	41.1	0.73	2.07	4.80	0.37	2.00	19.2	0.31	1.58	8.10	0.32	1.18	nd	1.61	3.15	6.19
OVCAR-3	0.29	0.87	3.14	0.45	3.50	>100	0.24	0.69	2.59	0.37	3.75	31.7	0.29	2.45	29.9	0.39	1.46	nd.	0.75	2.07	4.79
OVCAR-4	0.34	1.24	4.21	0.39	8.09	>100	0.31	1.35	4.21	0.28	5.55	37.3	0.24	2.83	37.7	0.28	1.43	4.04	1.40	2.83	5.75
OVCAR-5	1.36	2.96	6.42	2.14	10.6	>100	1.40	2.75	5.38	0.56	11.0	40.9	0.45	11.6	37.9	0.44	1.58	4.05	1.58	3.18	6.39
OVCAR-8	0.75	2.26	5.86	8.04	8.37	>100	1.05	3.27	>100	0.42	6.89	74.4	0.31	13.3	>100	0.35	1.65	>100	1.32	4.17	29.9
NCI/ADR-RES	1.29	2.72	5.73	58.4	>100	>100	1.16	2.64	5.99	18.0	>100	>100	2.88	16.7	>100	0.66	3.17	>100	2.94	18.0	>100
SK-OV-3	nd	nd	nd	nd	nd	nd	1.47	3.11	6.59	nd	nd	nd	nd	nd	nd	0.32	1.10	4.22	3.74	12.5	35.4
Renal Cancer																					

786-0	0.79	2.05	4.55	3.26	21.6	>100	1.24	2.62	5.53	1.87	12.5	40.7	0.37	2.80	23.1	0.39	1.69	nd	1.46	2.85	5.57
A498	0.63	2.10	4.77	nd	nd	nd	1.17	2.55	5.53	1.16	4.72	21.7	0.13	0.70	17.2	0.19	1.09	3.37	1.61	4.77	17.4
ACHN	0.69	1.93	4.40	3.06	13.6	>100	1.05	2.23	4.72	2.12	7.06	26.0	0.35	1.37	4.67	0.31	1.13	3.53	1.46	2.78	5.27
CAKI-1	1.10	2.39	5.21	2.20	6.28	>100	1.06	2.35	5.19	1.50	48.4	>100	2.46	9.36	31.2	0.27	1.08	3.44	1.36	2.71	5.41
RXF 393	0.65	2.14	5.41	1.16	5.42	27.5	0.81	2.22	5.41	0.60	3.21	17.5	0.31	2.38	19.3	0.35	1.45	4.63	1.40	2.79	5.58
SN12C	0.67	2.05	5.06	0.69	8.05	36.6	0.45	1.67	4.14	0.33	1.68	27.5	0.29	1.27	31.1	0.33	1.24	3.73	1.06	2.34	5.17
TK-10	1.10.	2.50	5.64	2.81	15.5	69.7	1.15	2.63	6.06	1.46	10.0	40.0	0.31	3.94	31.9	0.42	1.85	6.18	1.73	3.27	6.19
UO-31	0.32	1.40	3.95	3.13	14.4	66.1	0.45	1.77	4.21	1.82	5.06	18.2	0.31	1.47	4.98	0.27	1.21	3.50	1.32	2.60	5.10
Prostate Cancer																					
PC-3	0,41	1,58	5,14	0,45	6,43	>100	0,49	2,01	5,77	0,33	3,55	37,6	0,27	1,29	34,8	0,30	1,37	6,46	1,43	3,28	7,53
DU-145	1,03	2,41	5,64	0,79	11,8	55,5	1,28	2,63	5,40	0,47	10,4	37,3	0,35	3,57	26,7	0,43	1,53	4,26	1,27	2,52	5,02
Breast Cancer																					
MCF7	0.30	1.15	4.22	0.33	3.62	>100	0.34	1.50	5.02	0.31	1.32	33.7	0.25	1.39	41.6	0.29	1.11	5.23	1.52	3.83	9.68
MDA-MB-231/ATCC	0.53	1.82	4.61	0.71	6.16	67.6	0.67	2.04	4.74	0.37	2.40	27.1	0.29	1.20	5.78	0.31	1.25	4.54	1.52	2.93	5.64
HS 578T	1.14	3.49	13.40	1.09	8.27	>100	1.43	3.58	8.95	0.46	5.94	>100	0.27	2.03	>100	0.31	1.63	>100	1.70	5.32	>100
BT-549	1.15	2.38	4.93	0.35	3.85	31.2	1.61	3.11	5.99	0.36	4.11	32.3	0.27	1.88	29.9	0,35	1.32	4.22	1.61	3.10	5.98
T-47D	0.27	1.38	8.36	0.37	15.8	>100	0.44	2.40	nd	0.20	1.47	96.8	0.12	1.08	>100	0.26	1.22	nd	1.99	5.23	>100
MDA-MB-468	0.26	0.77	3.80	0.25	0.87	24.2	0.22	0.70	3.41	0.25	0.85	20.1	0.21	0.82	8.82	0.23	0.77	4.14	1.03	2.61	6.61
**Average values**	**0.62**	**1.80**	**5.38**	**1.29**	**9.55**	**63.10**	**0.77**	**2.12**	**5.68**	**0.60**	**6.31**	**38.02**	**0.31**	**2.29**	**22.91**	**0.34**	**1.34**	**4.90**	**1.48**	**3.72**	**11.48**

Table S3. Selectivity indices of compounds **1, 4** and **9**.

**Subpanel**	**Compound**								
	1			4			9		
	GI_{50}	TGI	LC_{50}	GI_{50}	TGI	LC_{50}	GI_{50}	TGI	LC_{50}
Leukemia	1,9	1.3	0.6[1]	1.0	1.0	1.4	1.2	1.1	NC
NSLC	1.1	1.0	1.1	1.0	1.0	1.1[2]	1.1	1.1	1.2[3]
Colon Cancer	1.4	1.2	1.2	1.5	1.1	1.2[4]	1.1	1.1	1.0[5]
CNS Cancer	0.6	0.8	1.1	0.7	0.9	1.1	1.1	1.0	1.2
Melanoma	1.3	1.2	1.3	1.1	1.0	1.1[6]	0.6	0.9	1.2
Ovarian Cancer	0.8	0.9	1.1	1.0	1.0	1.2[+]	0.9	0.8	2.4[8]
Renal Cancer	0.8	0.9	1.1	0.8	0.9	1.1	1.1	1.0	1.4[9]
Prostate Cancer	0.9	0.9	1.0	0.9	0.9	1.0	0.9	0.9	0.9
Breast Cancer	1.0	1.0	0.8	1.0	1.0	0.9[10]	1.2	1.1	1.1[11]

NC – not calculated (not enough data);
[1] Except for SR line ($LC_{50} > 100$);
[2] Except for HOP-62 and NCI-H226 lines (NT);
[3] Except for lines HOP-62 and NCI-H226 (NT);
[4] Except for lines COLO 205, HCT-116 (NT) and HT29 ($LC_{50} > 100$);
[5] Except for lines COLO 205 and HCT-116 (NT);
[6] Except for M14 line (NT);
[7] Except for OVCAR-8 line ($LC_{50} > 100$);
[8] Except for lines IGROV1 and OVCAR-3 (NT);
[9] Except for 786-0 line (NT);
[10] Except for T-47D line (NT);
[11] Except for lines HS 578T ($LC_{50} > 100$) and T-47D (NT);
NT - not tested.
